# Cysteine-Rich Peptide Fingerprinting as a General Method for Herbal Analysis to Differentiate Radix Astragali and Radix Hedysarum

**DOI:** 10.3389/fpls.2019.00973

**Published:** 2019-07-31

**Authors:** Jiayi Huang, Ka H. Wong, Stephanie V. Tay, Adrian How, James P. Tam

**Affiliations:** School of Biological Sciences, Nanyang Technological University, Singapore, Singapore

**Keywords:** herbal medicine, Radix Astragali, Radix Hedysarum, fingerprinting, cysteine-rich peptides, multivariate analysis, MALDI-TOF MS

## Abstract

Species misidentification and adulteration are major concerns in authenticating herbal medicines. Radix Astragali (RA), the roots of *Astragalus membranaceus*, is a traditional herbal medicine used for treating diabetes. However, it is often substituted by Radix Hedysarum (RH), the roots of *Hedysarum polybotrys* from the same plant family Fabaceae, which possesses different bioactivities. Current authentication methods, focusing on the chemical composition differences of herbal medicines based on small molecules, have limitations when these chemical markers are found in many species. Herein, we describe a rapid and general method using matrix-assisted laser desorption/ionization time-of-flight mass spectrometry (MALDI-TOF MS), coupled with multivariate analyses to differentiate herbal medicines. We used cysteine-rich peptide (CRP) fingerprinting, a method that exploits an underexplored chemical space between 2 to 6 kDa and which is populated by highly stable CRPs. To show the generality of the method, we screened 100 medicinal plant extracts and showed that CRP fingerprints are unique chemical markers. In addition, CRP fingerprinting was many-fold faster than the conventional authentication method using ultra-performance liquid chromatography (UPLC). Multivariate analyses showed that it has comparable classification accuracy as UPLC fingerprinting. Together, our findings revealed that CRP fingerprinting coupled with multivariate analyses is a rapid and general method for authentication and quality control for natural products in medicinal plants.

## Introduction

Misidentification of plant species is a major concern in the quality control of herbal medicines (Ekor, 2014). The confusion in the identity of the herbs may caused by several reasons: similar morphology, similar name, multiple sources, the presence of counterfeit and adulterants. A traditional way to authenticate herbal products is to quantify the major or most abundant compounds using chromatographic methods. Often, a single chemical marker is used as an indicator for quality assessment (Gad et al., 2013). However, this approach does not reflect the complexity of herbal products, and the chemical marker might not be unique to one herb. Another method employed for authentication is DNA barcoding, which is based on variations in the sequence of short standard DNA region(s). But the application of DNA barcoding has limitations because the DNA region in one plant is identical across many species, making it not a unique pattern (Pradhan et al., 2015). To overcome these limitations, fingerprint analysis, which reflects the unique pattern of chemical compositions in an herb, was adopted by global regulatory authorities such as the United States Food and Drug Administration (FDA), State Food and Drug Administration of China (SFDA) and the European Medicines Agency (EMEA) (Agency, 2001; Food and Drug Administration, 2004; Chinese Pharmacopoeia Commission, 2015). These chemical fingerprints can be obtained by spectroscopic or chromatographic techniques, such as high-performance liquid chromatography (HPLC), thin-layer chromatography (TLC), gas chromatography, capillary electrophoresis, and Raman spectroscopy (Liang et al., 2010; Tam et al., 2015). Among these techniques, chromatographic fingerprints obtained from HPLC are widely used due to its precision, sensitivity, and reproducibility. However, laborious sample preparation, relatively long analytical run-times and the large volume of organic solvents consumption in HPLC hinder its application as a high-throughput screening technique (Wong et al., 2014).

Mass spectrometry is an analytical technique used to detect the mass-to-charge ratio (*m/z*) of ions derived from analytes molecules, which can provide both qualitative and quantitative information about samples (Ho et al., 2003). The ability of mass spectrometry for analyzing non-volatile, thermally labile, intact and large biomolecules is due to the development of soft ionization techniques such as Matrix-Assisted Laser Desorption/Ionization (MALDI) and Electrospray Ionization (ESI) techniques (Jackson et al., 2000). Both ionization techniques provide a simple and efficient way for the routine mass spectroscopic analysis of peptides and proteins. However, direct infusion of ESI-MS is manual and need to inject one by one, which is time-consuming whereas MALDI is automatic. In addition, MALDI usually produces singly charges ions showing lower spectral complexity than ESI and is more robust in terms of their higher tolerance for salts than ESI (El-Aneed et al., 2009). Coupled with time-of-flight mass spectrometry (TOF MS), MALDI-TOF MS has been widely applied in various fields especially in the identification of large molecular compounds without prior chromatographic separation (Cai and Liu, 2014). Fingerprint analysis using MALDI-TOF MS has been employed to identify fungi species such as *Neoscytalidium* and *Penicillium* (Packeu et al., 2014). Furthermore, it has been applied to the quality control of food products such as Brazil grape species (Fraige et al., 2014) and *Campania* white wines. Peptidomic profiles derived from wine protein tryptic digests showed the unique fingerprinting of the samples (Chambery et al., 2009). Compared to HPLC, in addition to being faster and simpler, MALDI-TOF MS also has a larger detection range, has a higher tolerance to salts and buffers and requires minimal amounts of analytes (Cai and Liu, 2014).

The roots of *Astragalus membranaceus* Radix Astragali (RA), known as Huang Qi in Chinese, are popular herbal medicines used in traditional Chinese medicine (TCM) to increase overall vitality, treat diabetes and metabolic diseases (Cho and Leung, 2007). However, these roots are often misidentified or substituted by the roots of *Hedysarum polybotrys* (Radix Hedysarum, RH), a closely related species to *A. membranaceus*, which has similar morphology and Chinese name (Hong Qi). In addition, both species belong to the Fabaceae family, making it hard to distinguish RA from RH. However, the chemical constituents present in both species are different (Liu et al., 2012). It is reported that RH has been shown to possess a weaker antidiabetic activity *in vivo* compared to RA (Liu et al., 2010). In clinical practice, RH is employed to disperse swelling by external use, and incorrect use of RH in patients with diabetes may lead to fatal outcomes (Song et al., 2000).

Irrespective of the authentication methods, the major chemical markers are small-molecule secondary metabolites, generally with molecular weight < 1 kDa. These practices are well-documented in the Pharmacopoeia of the People’s Republic of China (PPRC) (Chinese Pharmacopoeia Commission, 2015) and Hong Kong Chinese Materia Medica Standards (Phase III) (HKCMMS Volume I and VIII, Hong Kong), the identification of medicinal herbs is based on quantification of their standard compounds using HPLC. The standard chemical markers of RH are ononin and formononetin while calycosin-7-*O* -β-D-glucoside is the standard compound to authenticate RA. Previously multiple methods were applied to detect the different chemical composition of RA and RH. A capillary HPLC (cHPLC) coupled with diode array detection (DAD) and MS method showed that ononin, calycosin, and formononetin are present in both species but with a significantly different amount (Zhao et al., 2008). In addition, the presence of secondary metabolites such as flavonoids and saponins in RA and RH have been comprehensively analyzed by HPLC–UV and HPLC–ELSD, which confirmed that saponins such as formononetin, calycosin, ononin are found in both species while calycosin-7-*O* -β-D-glucoside is present only in RA (Liu et al., 2012). Another study performed by HPLC showed that ononin, isomucronulatol 7-*O* -glucoside, calycosin and formononetin are found in RA and RH while medicarpin is the unique compound present in RH samples (Lee et al., 2012). Moreover, DNA barcoding based on internal transcribed spacers (Lee et al., 2012) and 5S-rRNA spacer domains (Ma et al., 2000), have been used for identifying RA and RH.

Cysteine-rich peptides (CRPs) are generally hyperstable. They have well-defined structures stabilized by three or more cross-linking disulfide bridges that render them resistant to thermal, chemical and enzymatic degradation (Tan et al., 2017). However, the chemical spaces based on the molecular mass of CRPs of the plant-derived natural products have not been seriously used as authentication standards (Wong et al., 2016). The hyper-stability of CRPs is essential as putative compounds in herbal medicine because they generally require decoction or other processing steps., Our laboratory are particularly interested in CRPs with molecular weights ranging from 2 to 6 kDa, and which are readily detected by MALDI-TOF MS, a space which is uncluttered by small-molecule metabolites. Another advantage of CRPs is that they are well-annotated because they are grouped into families, such as thionins, defensins, hevein-like and knottin-type peptides based on their cysteine motifs and disulfide connectivity (Wong et al., 2017b). In addition, our studies on CRPs showed that they are widely distributed *in planta* and could used for authentication (Kini et al., 2015, 2017; Kumari et al., 2015; Nguyen et al., 2015a,b; Wong et al., 2016, 2017a,b; Tan et al., 2017; Tam et al., 2018; Shen et al., 2019). In our previous study, we characterized a group of CRPs from RA (Huang et al., 2019). We hypothesized that the unique chemical space of CRPs is suitable for discriminating different plant species, and thus can authenticate RA and differentiate it from its closely related species RH.

In addition to the instrumental analyses, multivariate data analysis techniques were introduced for the quality control because of the complexity of herbal medicines to detect minor differences between closely related species. Instead of relying on the comparison to a reference compound or on quantifying a particular chemical marker, multivariate analyses usually combine mathematical and statistical techniques to increase the understanding of chemical data and also to correlate the quality parameters of physical properties of the analytical instrument data (Biancolillo and Marini, 2018). The pattern recognition models in multivariate analyses can improve the overall classification efficiency based on the chromatographic or spectroscopic fingerprint obtained.

Here, we describe a CRP fingerprinting method to differentiate RA and RH. To show the generality of our method, we screened 100 herbs and herbal products and showed that the CRP fingerprinting produces consistent results. In a case of RA and RH, we used MATLAB and classification built-in tools to extract and analyze the spectra from MALDI-TOF MS and chromatograms from UPLC. Our results suggest that this combination can provide a powerful tool for differentiating closely related plant species and herbal products.

## Materials and Methods

### Solvent and Chemicals

Medicarpin (>98%), formononetin (>98%), calycosin-7-*O* -β-D-glucoside (>98%), and calycosin (>98%) were purchased from Chengdu Biopurify Phytochemicals, Ltd. (Chengdu, China). Ononin (>98%) was purchased from Sigma-Aldrich (St. Louis, MO, United States). HPLC-grade acetonitrile and trifluoroacetic acid were obtained from Thermo Fisher Scientific (Singapore). Milli-Q water was purified by a Milli-Q water purification system from Millipore (MA, United States).

### Plant Materials

Hundred plants and herbal medicines were collected and purchased from various region of China and Singapore (Supplementary Table S1). 40 RH and 51 RA samples were collected from herbal pharmacies in various regions of China and Singapore (Supplementary Table S2). The taxonomic identification was carried out macroscopically and microscopically according to the descriptions mentioned in the PPRC (Chinese Pharmacopoeia Commission, 2015). The samples were authenticated by an experienced registered TCM physician from Nanyang Technological University, Singapore, and voucher specimens were deposited at the Nanyang herbarium, School of Biological Sciences, Nanyang Technological University, Singapore.

### CRP Fingerprinting

Each dried sample was ground using a pulverizer and passed through a No. 180 (177 μm) sieve. Then, 150 mg of each sample from the two species was accurately weighed and extracted with 1.5 mL of Milli-Q water or 50% ethanol. The mixture was vortexed at room temperature for 1 h before being centrifuged at 10,000 × *g* for 15 min. The supernatant was filtered through Whatman No. 1 filter paper under vacuum. A Strata-X Polymeric Reversed Phase micro elution 96-well plate (Phenomenex, CA, United States) was used for sample preparation of the crude extracts for mass spectrometry analysis. Each well was percolated with water and the filtrate of the samples was loaded onto different wells under vacuum. The desired peptides were eluted with 80% (v/v) acetonitrile. Prior to MALDI TOF-MS analysis, 0.5 μL of matrix containing a saturated solution of α-cyano-4-hydroxycinnamic acid in 80/20 (v/v) acetonitrile/water was mixed with each sample (0.5 μL). The mixture was then spotted onto a MALDI plate and dried at room temperature. Mass spectra of the samples were obtained with an ABI 4800 MALDI-TOF MS mass spectrometer (Applied Biosystem, MA, United States). The MALDI-TOF MS was operated in positive ion reflector mode, acquiring 2000 shots (20 positions per spot; 100 shots per position) with a laser intensity at 5500. The accelerating and grid voltages were set at 20 and 16 kV, respectively. The extraction and MS scan were performed in triplicate for each sample.

### Isolation and Characterization of CRPs From RH and RA

Dried RA (1 kg) was homogenized in 10 L of Milli-Q water and stirred for 2 h at room temperature. The homogenate was then centrifuged at 8,000 × *g* for 15 min and the supernatant was loaded onto a C_18_ reversed-phase (RP) flash column. An increasing concentration of ethanol (20-80%) was used for sample elution. Fractions with the desired peptides were pooled and purified by multiple runs of preparative RP-HPLC using a C_18_ column (particle size, 5 μm, 250 × 21 mm; Phenomenex, CA, United States) on a Shimadzu HPLC system (Shimadzu, Kyoto, Japan). A linear gradient from buffer A (Milli-Q water with 0.1% trifluoroacetic acid) to buffer B (acetonitrile with 0.1% trifluoroacetic acid) was applied.

The primary sequences of CRPs obtained from RA were determined as described previously (Huang et al., 2019). Briefly, Astratides (10 μg) were reduced by incubating with 50 mM dithiothreitol in 20 mM ammonium bicarbonate buffer (pH 7) at 37°C for 1 h. The reduced peptide was then alkylated with 100 mM iodoacetamide at 37°C for 1 h. Subsequently, the sample was desalted using a C18 Zip-tip and lyophilized. The peptide was redissolved in 0.1% formic acid and analyzed by a Dionex UltiMate 3000 UPLC system (Thermo Fisher Scientific, Bremen, Germany) coupled with an Orbitrap Elite mass spectrometer. Peptide separation was performed with a 60 min gradient using buffer A (0.1% formic acid) and buffer B (90% acetonitrile/0.1% formic acid). LTQ Tune Plus software (Thermo Fisher Scientific, Bremen, Germany) was set to a positive mode for data acquisition. A Michrom’s Thermo CaptiveSpray nanoelectrospray ion source (Bruker-Michrom, Auburn, CA, United States) was used to generate the spray. The data were acquired by alternating the Full FT-MS/MS as previously described (Wong et al., 2016). PEAKS studio version 7.5 (Bioinformatics Solutions, Waterloo, ON, Canada) was used to process the data acquired from the LC/MS-MS analysis. A Parent error tolerance of 10 ppm and a fragment error tolerance of 0.05 Da were applied.

The isolation of CRPs from RH was performed in the same manner as performed for RA. The primary sequence of hedytides isolated from RH was determined by MALDI-TOF MS/MS. Peptides (10 μg) were reduced with 50 mM dithiothreitol in 20 mM ammonium bicarbonate buffer (pH 7) at 37°C for 1 h. Subsequently, hedytides were digested with trypsin or chymotrypsin (Roche, Basel, Switzerland) at a ratio of 1:5 (enzyme: hedytide) in 10 mM hydrochloric acid at 37°C for 30 min. The peptide fragments obtained were then subjected to MALDI TOF MS/MS analysis. The primary sequence was determined by interpreting the b- and y-series ions formed during the MS/MS fragmentation.

### Sample Preparation for UPLC Analysis

The method used was modified from the Hong Kong Chinese Materia Medica Standards (Phase III) (HKCMMS Volume I and VIII, Hong Kong) and previous studies on the chromatographic analyses of RA and RH. Each sample (50 mg) of RA and RH was weighed and extracted with 1 mL of 80% methanol. The mixture was sonicated for 1 h before being centrifuged at 3000 × *g* for 5 min, and the supernatant was filtered through a 0.45 μm PTFE filter. All sample preparation was performed in triplicate. All of the extracts were evaporated to dryness for approximately 3 h in an Eppendorf Concentrator Plus^TM^ (Eppendorf, Hamburg, Germany). The dried residues were redissolved in 100 μL of 80% methanol in a sonication bath and were centrifuged at 8000 rpm for 10 min. Then, the supernatant was stored in a glass scintillation vial at −20°C prior to chromatographic analysis.

### UPLC Analysis

The analysis was carried out using the Nexera X2 UPLC system (Shimadzu, Kyoto, Japan) coupled with an Aeries ^TM^ PEPTIDE XB-C_18_ column (3.6 μm, 100 mm × 2.1 mm, Phenomenex, CA, United States). A binary gradient elution method at a flow rate of 0.3 mL min^–1^ was employed using 0.1% trifluoroacetic acid in Milli-Q water as buffer A and 0.1% trifluoroacetic acid in acetonitrile as buffer B, as follows: 10% B at 0.00–3.00 min, 10–30% B at 3.00–20.00 min, 30–38% B at 20.00–42.00 min, 38–80% B at 42.00–42.01 min, 80% B at 42.01–44.00 min, 80–10% B at 44.00–44.01 min, and 10 − 10% B at 44.01–46.00 min. The detection wavelength was set to 230 nm. The chromatograms were documented and analyzed by Shimadzu LabSolutions Data software.

### UPLC Validation

The validation of the UPLC method such as linearity, range, accuracy, and precision was performed according to the guideline of the International Conference on Harmonization of Technical Requirements for Registration of Pharmaceuticals for Human Use (ICH) (International Council for Harmonisation of Technical Requirements for Pharmaceuticals for Human Use Guideline, 2005). To evaluate the linearity and range of the method, serial dilutions of standard compounds including medicarpin, formononetin, calycosin-7-*O* -β-D-glucoside, calycosin and ononin with 80% methanol were used to generate calibration curves. Each calibration curve was established by running the authentic standard compound at more than 10 concentrations (0.02–3000 μg mL^–1^) in triplicate. The calibration curve was obtained by plotting the average peak area versus the concentration of each standard compound. According to the ICH guideline, the limit of detection (LOD) was calculated using the formula 3.3^*^σ/slope and the limit of quantification (LOQ) was calculated as 3.3^*^σ/slope while σ was defined as the standard deviation.

The accuracy and precision were measured by analyzing in triplicate the quality control (QC) samples at low QC (LQC), medium QC (MQC), and high QC (HQC) concentrations (calycosin-7-*O* -β-D-glucoside: 250, 500, and 1000 μg mL^–1^; formononetin: 50, 100, and 200 μg mL^–1^; calycosin: 160, 320, and 640 μg mL^–1^; medicarpin: 200, 400, and 800 μg mL^–1^; and ononin 375, 750, and 1500 μg mL^–1^). The intraday precision and accuracy of each standard were determined by injecting standards at different QC concentrations six times within 1 day. By analyzing the QC samples on three consecutive days, in which the standards were injected six times daily, the interday precision and accuracy were determined. The relative standard deviation [RSD (%)] was used to show the precision, whereas accuracy was expressed as the relative error [RE (%)]. The formulas were determined as:

$$\mathrm{Relative}\,\mathrm{standard}\,\mathrm{deviation}\,\left(\mathrm{RSD}\right)\%=\frac{\mathrm{standard}\,\mathrm{deviation}\,\left(\mathrm{SD}\right)}{\mathrm{mean}}\times 100$$

$$\mathrm{Accuarcy}\%=\frac{\mathrm{mean}\,\mathrm{observed}\,\mathrm{concentration}-\mathrm{spiked}\,\mathrm{concentration}}{\mathrm{spiked}\,\mathrm{concentration}}\times 100$$

### Data Preprocessing

#### MALDI-TOF MS Data Matrix

The spectra obtained from the MALDI-TOF MS were converted to data points in ASCII format using Data Explorer V4.9 (Applied Biosystem, MA, United States). The number of data points per sample was set as 30935. The mean of the three spectra obtained from each sample was used as the final data set and combined to form a MALDI-TOF MS data matrix consisting of 91 rows (number of samples) and 30935 columns (number of data points per sample). Several preprocessing techniques were applied to the raw MALDI-TOF MS data matrix. To smooth the matrix, a peak alignment procedure called correlation optimized warping (COW) was applied. An algorithm for choosing the ideal reference spectrum and the best segment length and slack number proposed by Skov et al. (2006) was applied to optimize the procedure. Other preprocessing programs such as standard normal variate were used to remove slope variation among spectra. Mean cantering was also used to remove the column mean from each variable of the corresponding column.

The data matrix was divided into a calibration set and validation set based on the Kennard-Stone algorithm (Daszykowski et al., 2002), which was applied to the RH and RA samples separately. Generally, 60% of the samples with the greatest deviations were selected as the calibration set while the remaining 40% samples were used as the validation set. Additionally, mean-centering, standard normal variate, and normalization were applied onto the data matrices to enhance the signal-to-noise ratio and the interpretability of the models. The effects of various preprocessing steps on partial least square discriminant analysis (PLS-DA) models were evaluated and compared. The model showing the best classification ability was selected for further analysis.

#### UPLC Data Matrix

In a chromatographic analysis, a retention time shift may occur due to the changes in mobile phase composition, operator handling and instrumental instability (Wong et al., 2013). Thus, similar to the MALDI-TOF MS data matrix preprocessing, COW was applied to align the peaks in the UPLC data matrix while the baseline elevation was eliminated by subtracting a blank chromatogram. Standard normal variate and mean centering were performed before classification analysis. The Kennard-Stone algorithm was used to separate the data matrix into calibration and validation sets as in the same manner performed on the MALDI-TOF MS data set.

### Multivariate Analyses

#### Unsupervised Multivariate Analyses

##### Principal component analysis (PCA)

Principal component analysis is an unsupervised multivariate analysis used for separating a pool of variables into different clusters in predictive models and for exploratory data analysis. Generally, PCA reduces large data sets by projecting them onto lower dimensions called principal components, aiming to determine the best trend of the data using a limited number of principal components (Varmuza and Filzmoser, 2016). In this study, prior to model calibration, PCA was performed on the MALDI-TOF MS and UPLC data matrices for outliner determination and sample classification. The optimal principal components for the MALDI-TOF MS and UPLC matrices were both determined to be 3.

##### Hierarchical cluster analysis (HCA)

Hierarchical cluster analysis is an unsupervised multivariate method used for natural grouping among samples characterized by their features. Strategies for HCA can be classified into two main categories: agglomerative and partitional. Agglomerative methods usually start with each object being its own cluster and pairs of clusters are merged hierarchically into larger ones, while partitional method begins with a single cluster containing all objects and splits existing clusters into smaller ones (Rokach and Maimon, 2005). Usually, agglomerative methods are more commonly used in chemometric studies. There are six agglomerative methods which include the nearest neighbor, furthest neighbor, pair-group average, centroid, median, and Ward’s method, based on their inter-cluster distance and linkage rules. In this study, Ward’s method and squared Euclidean distance were used, which minimized the numbers of clusters and the deviation of any two clusters for each step (Chlebda et al., 2016).

#### Supervised Multivariate Analyses

##### Partial least square-discriminant analysis (PLS-DA)

In contrast to PCA, PLS-DA is a supervised analysis method that maximizes the separation between predefined classes rather than explaining the variation with each class. In PLS-DA, the data matrices were projected onto latent variables to maximize the covariance between the original matrix and the predefined response classes (Rosipal and Krämer, 2005). The predictions from a PLS-DA model are qualitative and normally coded in vectors (Wong et al., 2013). The UPLC and MALDI-TOF MS data matrices were divided into two subgroups, RH and RA, based on their botanical characteristics. Consequently, RH and RA were represented by vector numbers 0 and 1 and a *y* predicted response value of each unknown sample was calculated. A predicted value close to 1 indicated that the corresponding sample belonged to the considered class, while a value close to 0 means that the sample was rejected as a member of the class. The optimal latent variables were determined as 2 and 1 in the MALDI-TOF MS and UPLC data matrix, respectively.

##### K-nearest neighbors (KNN)

K-nearest neighbors is an instance-based algorithm that utilizes the distance between samples in the p-space as its primary criterion. The classification was performed based on the Euclidean distance between samples. Unknown samples were classified based on their distance from other data points nearest to them and the majority vote of the neighbors. *K* -value, the optimal number of the nearest neighbor, was determined by leave-one-out cross-validation (Zadeh, 1965). The optimal *K* -values for the UPLC and MALDI-TOF MS data matrices were both determined as 3.

##### Classification and regression tree (CART)

Decision trees are used in creating a model that predicts the value of a target based on the values of independent variables. CART is a non-parametric decision tree that produces a classification of regression trees depending on whether the variables are categorical or continuous, respectively (Frank and Lanteri, 1989). Since there were only two classes involved, the optimal tree size was not determined.

##### Soft independent modeling of class analogy (SIMCA)

Soft independent modeling of class analogy is a supervised classification method that minimizes the assumptions about the linearity of relationships between samples and predefined classes (Wong et al., 2014). To build the model, each class (RH and RA) needs to be analyzed using PCA separately. Hence, a principal component model was used to account for most of the variation within each class. Because the number of principal components retained for each class is usually different, the cross-validation set was used to select the optimal numbers of principal components. To classify an unknown sample, its matrix was projected to each established PCA model and the residual distance was calculated. By comparing the residual variance of the unknown sample to the average residual variance of the PCA model from each class, the unknown sample was able to be categorized (Frank and Lanteri, 1989). The optimal principal components for the RA and RH PCA model were determined to be 5 and 1 in the MALDI-TOF MS and UPLC data matrix, respectively.

##### Support vector machine-discriminant analysis (SVM-DA)

Support vector machine-discriminant analysis is a supervised classification method that is commonly used for binary classification. In SVM-DA, samples are represented by points in two classes. Based on this, a hyperplane boundary that separates all points to place the majority in the same class was calculated (Wong et al., 2014). The technique aims to determine the optimal hyperplane that can maximize the distance between the two separated classes (Yan et al., 2016). In this study, the X-block compression was set at “none” and the probability estimation was set at “on.”

### Classification Model Performance Evaluation

All models were cross-validated with Venetian blind and split into ten blocks (Varmuza and Filzmoser, 2016). Confusion matrices were used to evaluate and compare the performances of the classification models. In a confusion matrix, the error rate (ER), non-error rate (NER), sensitivity, and specificity were calculated using the following equations:

$$\mathrm{sensitivity}=\frac{\mathrm{Number}\,\mathrm{of}\,\mathrm{True}\,\mathrm{Positive}}{\mathrm{Number}\,\mathrm{of}\,\mathrm{True}\,\mathrm{Positives}+\mathrm{Number}\,\mathrm{of}\,\mathrm{False}\,\mathrm{Negative}}$$

$$\mathrm{Specificity}=\frac{\mathrm{Number}\,\mathrm{of}\,\mathrm{True}\,\mathrm{Negative}}{\mathrm{Number}\,\mathrm{of}\,\mathrm{True}\,\mathrm{Negative}+\mathrm{Number}\,\mathrm{of}\,\mathrm{False}\,\mathrm{Positive}}$$

$$\mathrm{NER}=\frac{\mathrm{Sensitivity}+\mathrm{Specificity}}{\mathrm{Number}\,\mathrm{of}\,\mathrm{class}}$$

ER=1-NER

True Positive, RA samples correctly classified as RA; True Negative, RH samples correctly classified as RH; False Negative, RA samples wrongly classified as RH; False Negative, RH samples wrongly classified as RA.

### Software

Data processing was performed on MATLAB R2017b (The MathWorks, MA, United States). Classification modeling was analyzed on PLS toolbox version 8.5 (Eigenvector Research, WA, United States) and classification toolbox version 5.1. The Kennard-Stone and COW algorithms were developed by Daszykowski et al. (2002) and Skov et al. (2006), respectively.

## Results and Discussion

### CRP Fingerprinting of 100 Herbs and Herbal Products

To show the generality of CRP fingerprinting and that CRPs are widely present in plant species, we used MALDI-TOF MS analysis for screening putative CRPs in 100 herbs and herbal products. They include important herbs such as *Panax ginseng*, *Panax notoginseng*, and *Panax quinquefolius*. In addition, our screening included herbs in dried, fresh and processed granule forms. It is noteworthy to point out that DNA barcoding is not applicable for herbs in processed granule form. Our results showed that clusters of peptides within the mass range from 2 to 6 kDa are present in all 100 herbs and herbal products (Supplementary Figure S1). To show that they are CRPs, we treated the samples with a disulfide-reducing agent followed by an *S* -alkylating agent, a procedure commonly used in our laboratory. A mass shift before and after *S* -reduction of the disulfides with dithiothreitol and *S* -alkylation of the free thiols with iodoacetamide, results in a mass increment of 58 Da for each cysteine, and which confirmes that they are CRPs (Kini et al., 2015, 2017; Kumari et al., 2015; Nguyen et al., 2015a,b; Wong et al., 2016, 2017a,b; Tan et al., 2017; Tam et al., 2018; Shen et al., 2019). All 100 mass spectra showed all these peaks in the region between 2 to 6 kDa are CRPs. Thus, our results suggest that CRPs are useful chemical markers with different molecular weight and amino acid composition that are widely distributed *in planta*.

There are three major characteristics to access whether the fingerprints are well suited for use in the quality control of herbs: Distinctness, uniformity and stability. Our screening result of 100 herbs showed the distinctness of the CRP fingerprints which are unique and clearly distinguishable from the others in each plants. To confirm the uniformity of CRP fingerprints, we compared the CRP profiles of *Viola yedoensis* in different forms (Supplementary Figure S2). When viewed as a whole fingerprint, the CRP profiles of *V. yedoensis* remain relatively consistent amongst the various forms of the herb. Furthermore, to be useful in the quality control of plants and herbal products, the biological fingerprints must be able to survive the harsh decoction process. To show the stability of CRPs, the aqueous extracts of *Triticum aestivum* were placed in a water bath (90°C) for 1 h. The CRP fingerprints of *T. aestivum* were shown to remain consistent despite the harsh conditions (Supplementary Figure S3). The distinctness, uniformity and stability of CRPs underline the usefulness of utilizing CRP fingerprinting for the quality control of herbs.

**FIGURE 1:**
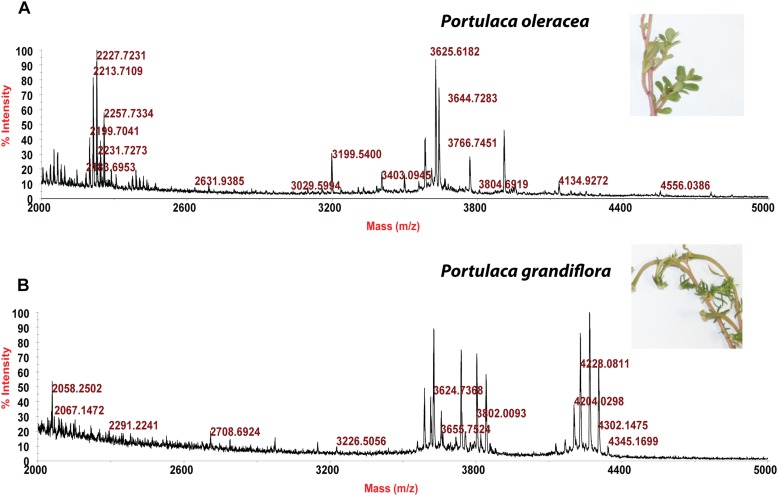
MALDI-TOF mass spectra of two plants with similar morphology: **(A)** *Portulaca oleracea* and **(B)** *Portulaca grandiflora.*

**FIGURE 2:**
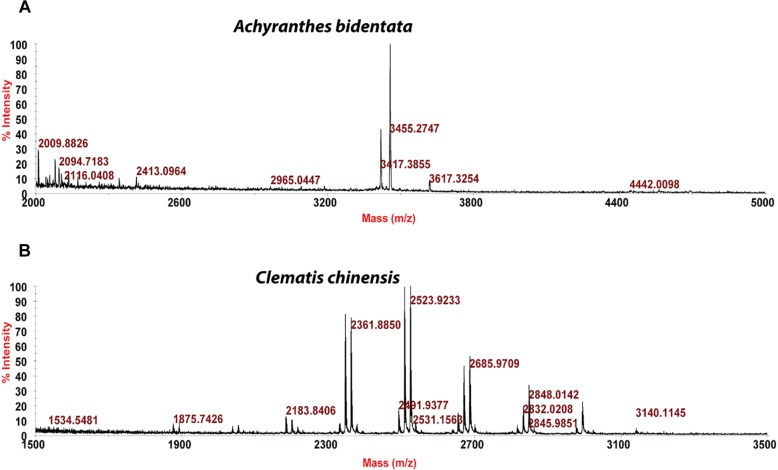
MALDI-TOF mass spectra of two plants with similar chemical composition: **(A)** *Achyranthes bidentata* and **(B)** *Clematis chinensis*.

**FIGURE 3:**
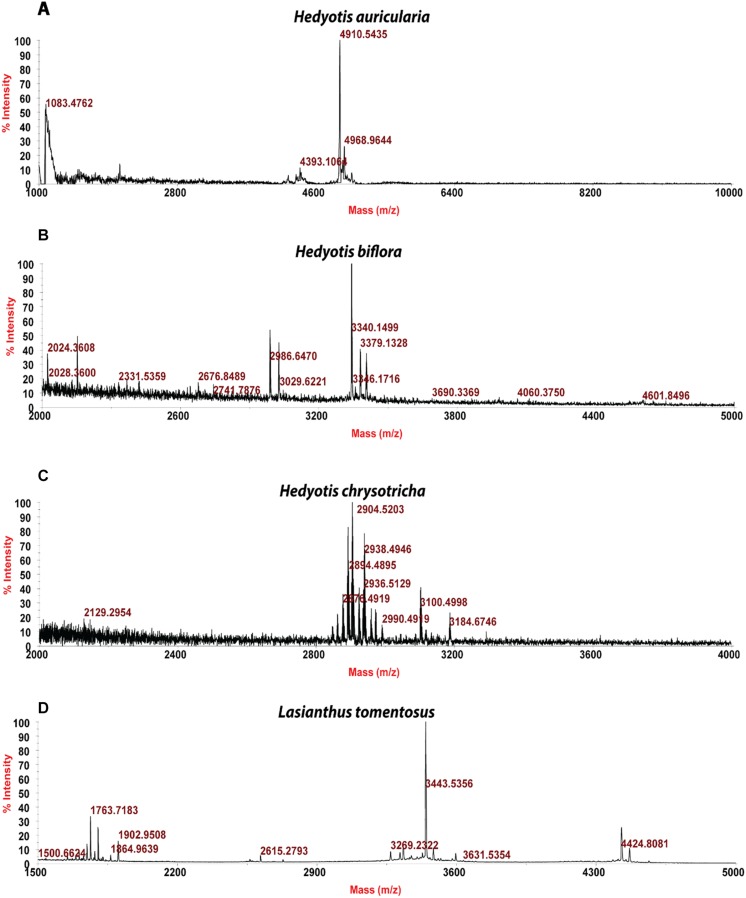
MALDI-TOF mass spectra of four plants from Rubiaceae family: **(A)** *Hedyotis auricularia*, **(B)** *Hedyotis biflora*, **(C)** *Hedyotis chrysotricha*, and **(D)** *Lasianthus tomentosus.*

Usuallly, most identification of plants are based on visual analysis of their morphological features, which are subjective and not accurate when many plants share similar morphological characteristics. Our results showed that by employing CRP fingerprints as chemical markers, it can distinguish between plants with similar morphologies. For example, *Portulaca oleracea* and *Portulaca grandiflora* are two herbs with similar morphologies. Using our screening procedure, we obtained the unique CRP fingerprints of these two plants and the presence of these “marker” peaks allows us to establish the identity of each plant (Figure 1). Another common quality control method is employed based on the different chemical constituents of the herbs. However, sometimes these chemical markers are not unique and widely expressed in many species. In our study, we showed that by employing CRP fingerprinting, it was able to distinguish between plants with similar chemical composition. For example, oleanolic acid was expressed in two herbs, *Achyranthes bidentata* and *Clematis chinensis.* By employing oleanolic acid as a chemical marker according to the Chinese Pharmacopeia, it is difficult to differentiate these two species. In contrast, the CRP fingerprints presented in *A. bidentata* were distinguishable from the CRP fingerprints of *C. chinensis* (Figure 2). Similar results can be observed in plants from same plant families (Figure 3), which suggests that CRP fingerprinting can differentiate species regardless of their origins.

**FIGURE 4:**
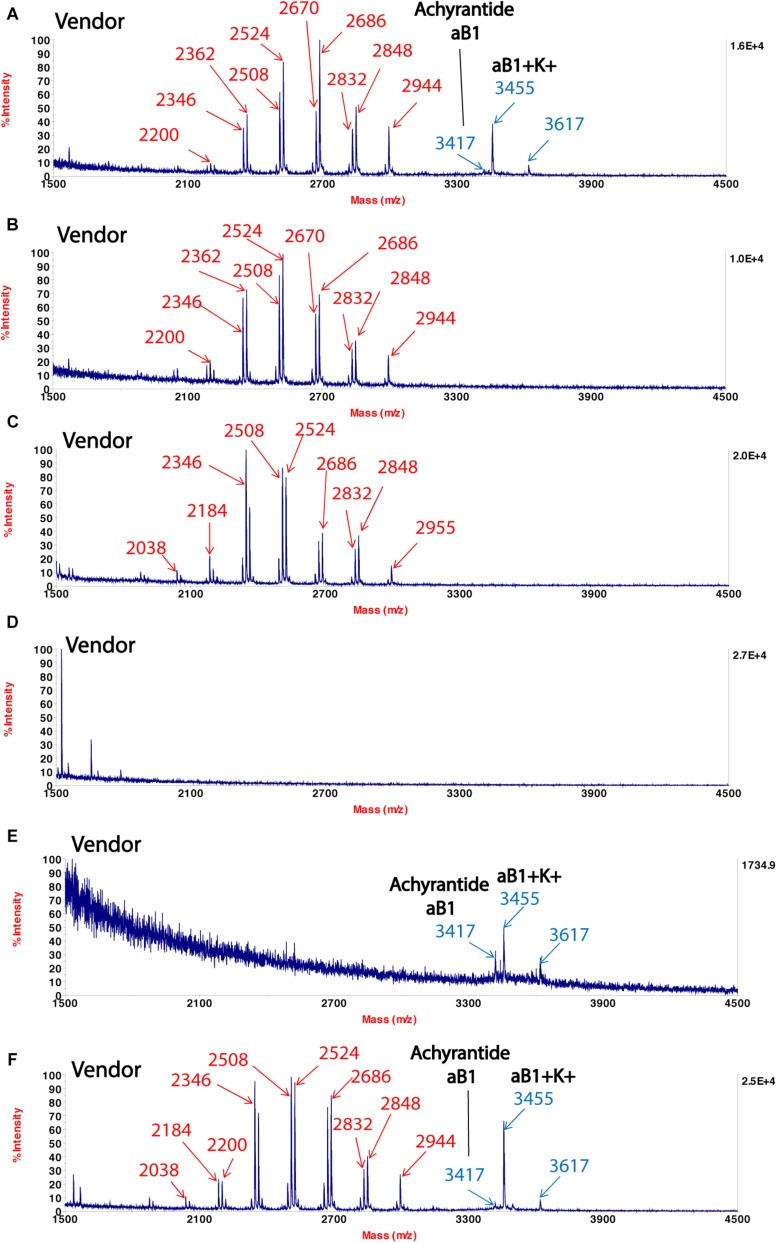
MALDI-TOF mass spectra of Shu-Jing-Huo-Xue-Tang obtained from different vendors **(A–F)**. Marker peaks corresponding to *Achyranthes bidentata* are shown in blue, while marker peaks corresponding to *Clematis chinensis* are shown in red.

To show that CRP fingerprinting can be used for quality control of complex TCM formulation even in granular forms, we used Shu-Jing-Huo-Xue-Tang 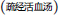 as an example. This formulation comprises of 17 herbs, of which two key herbs are *A. bidentata* and *C. chinensis. A. bidentata* 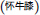 is often substituted by *Cyathula officinalis* 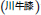 which bears the same Chinese name 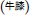. We showed CRP fingerprints of six Shu-Jing-Huo-Xue-Tang which were purchased form Singapore vendors (Figure 4). We identified the peaks in the six samples and found that the peaks < 3 kDa are saponins of *C. chinensis*. In contrast, *S-* reduction showed that peaks with molecular mass ranging from 3 to 4 kDa are CRPs from *A. bidentata* and designated as achyranthes (Supplementary Figure S4). *De novo* sequencing revealed that achyranthes aB1 is a novel CRP containing six cysteines with the full sequence of CLESGTSCIPGAPHDCCSGVCIPIVTVFYGKCY. Achyranthes aB1 belongs to the CRP family known as six-cysteine hevein-like peptides (Tam et al., 2015, 2018). Our results illustrates two important points of CRP fingerprinting in herbal authentication: (1) not all formulation contains the key ingredient, achyranthes, and (2) the concentrations of key ingredient varies from batch to batch. For example, *A. bidentata* was only found in samples obtained from venders A, E, and F. Our results demonstrate the usefulness of CRP fingerprinting in the quality control of herbal formulations.

### CRP Fingerprinting of RH and RA

**FIGURE 5:**
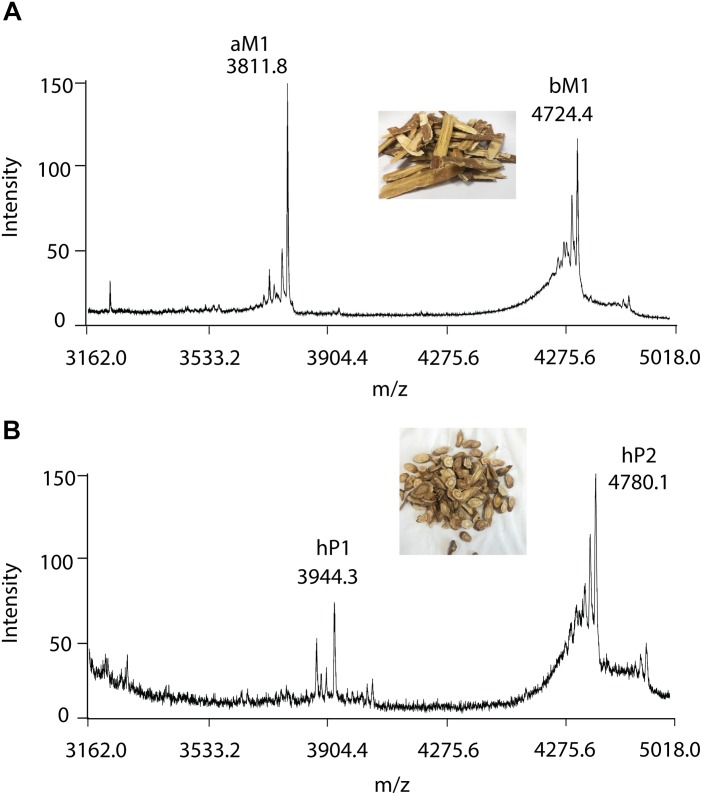
MALDI-TOF MS profile of **(A)** RA and **(B)** RH samples. Two major CRPs are designated as aM1 and bM1, with a molecular weight of 3811.8 and 4724.4 Da in RA samples, respectively. Similarly, with molecular weights of 3944.3 and 4780.1 Da, two major CRPs in RH samples were designated as hP1 and hP2, respectively.

To further validate the method, RH and RA were used as examples. A small-scale screening revealed clusters of putative CRPs with molecular mass ranging from 3 to 5 kDa in both RH and RA (Figure 5). In the RA samples, two major peaks with *m/z* of 3811.8 and 4724.4 Da were observed and designated as astratide aM1 and bM1, respectively. Previously, our laboratory has identified these two peaks with the aM1 sequence as VDCSGACSPFEVPPCGSRDCRCIPIGLVVGFCIYPTG and the bM1 sequence as CEKPSKFFSGPCIGSSGKTQCAYLCRR GEGLQDGNCKGLKCVCAC, respectively (Huang et al., 2019).

Similarly, in the RH samples, two major peptide peaks with *m/z* of 3944.3 and 4780.1 Da were observed and designated as hedytides hP1 and hP2. Since the sequences of hP1 and hP2 have not been reported, we used MALDI-TOF MS/MS to determine their sequences. After trypsin digestion, hP1 yielded two fragments with *m/z* of 3416 and 550 Da whereas hP2 yielded three fragments with *m/z* of 1433, 2272, and 1121 Da. The amino acid assignment of the digested fragments was performed based on the *b-* and *y-* ions detected during the tandem MS fragmentation. *De novo* sequencing of the digested fragments gave the full sequence of the 37-residue hP1 as QGCNGPCTPFEQPPCGIQSCRCFPEVLFFGRCSTPSG. The process was repeated to determine the full sequence of the 45-residue hP2 as CEKGSEFFVGACRYSEGTQQCATLCSRGEG LQGGKCKGVRCYCSC (Supplementary Figures S5, S6).

Plant CRPs are classified into different families such as defensins, knottins, hevein-like peptides, thionins, and α-hairpinins, based on their different sequences, cysteine spacing and disulfide connectivity. Our previous study showed that aM1 belongs to pea albumin 1 b-like peptides, whereas bM1 is a plant defensin (Huang et al., 2019). Both aM1 and hP1 are 37 amino acids in length and contain six cysteines. Sequence comparison revealed that they share a 65.7% sequence similarity and comprise the same cysteine motif of C-X_3_-C-X_7_-C-X_4_-C-X-C-X_9_-C. Similarly, both bM1 and hP2 are 45 amino acids in length with 62.2% sequence identity. They share similar cysteine motif of C-X_10_-C-X_8_-C-X_3_-C-X_10_-C-X_4_-C-X-C-X-C. Based on the cysteine motif and the sequence identity, we concluded that hP1 is a pea albumin 1 b-like peptide similar to aM1, and hP2 a plant defensin similar to bM1.

This intra-family sequence similarity and variability were frequently observed in the legume family. It was reported that PA1b-like peptides ranging from 3 to 4 kDa present in more than 18 species in the Fabaceae family, with sequence identity ranging from 61% (between soybean and *Alysicarpus ovalifolius*) to 86.1% (between aM1 and *Glycine max*) (Louis et al., 2007). In addition, defensins have been identified in >10 species from the Fabaceae family with 46.8–86.7% sequence identity (Huang et al., 2019). Although these CRPs are classified under the same CRP subfamily, their sequence variability could be used for distinguishing one plant from another. Furthermore, the ability to withstand harsh conditions during the processing stage of crude herbal medicine makes CRPs as suitable chemical markers for the differentiation of RA and RH.

### Chromatographic Method Validation

Limit of detection, LOQ, and calibration curve parameters of each standard compound were summarized in Supplementary Table S3. Low LOD and LOQ values of ≤0.15 and 0.43 μg mL^–1^, respectively, for all five standard compounds were observed. High correlation coefficients (*r* ^2^ ≥ 0.9990) and a wide linear range (0.02–3000 μg mL^–1^) indicated the highly correlated relationship between the reference compounds and the peak area. The intraday and interday precision and accuracy for standard compounds at low, medium, and high concentrations are shown in Table 1. The average RSDs of intraday LQC, MQC, and HQC were 0.66, 0.48, and 0.30%, whereas 1.42, 1.09, and 0.93% were the averages for interday, respectively. Moreover, the average of intraday accuracies at LQC, MQC, and HQC were 2.11, 2.90, and 2.09%, whereas the average interday accuracies were 1.73, 2.73, and 1.50%, respectively. The results showed that the developed chromatographic methods had good accuracy and repeatability.

**TABLE 1:** Validation of the intra- and inter-day accuracies of five standard compounds at low, medium, and high concentrations.

Compounds	Spiked concentration (μg mL^–1^)	**Intra-day (*n* = 6)**			**Inter-day (*n* = 18)**		
			
		Observed concentration (μg mL^–1^)^a^	Precision RSD (%)^b^	Accuracy (%)^c^	Observed concentration (μg mL^–1^)^a^	Precision RSD (%)^b^	Accuracy (%)^c^
calycosin-7-*O* -β-D-glucoside	250	252.995±0.914	0.361	1.197	257.999±3.793	1.470	3.199
	500	519.093±2.716	0.523	3.818	520.266±2.424	0.466	4.053
	1000	967.190±2.325	0.240	–3.281	975.803±7.011	0.718	–2.451
formononetin	50	50.963±0.876	1.719	1.927	50.081±1.116	2.227	0.162
	100	98.997±1.349	1.362	–1.002	101.801±2.857	2.806	1.801
	200	189.891±1.312	0.691	–5.084	193.855±4.552	2.348	–3.072
calycosin	160	161.065±0.898	0.558	0.666	160.424±0.999	0.623	0.265
	320	316.580±0.800	0.253	–1.069	317.512±1.646	0.518	–0.777
	640	637.987±2.264	0.355	–0.315	636.471±3.514	0.552	–0.551
medicarpin	200	209.377±0.941	0.449	4.688	204.941±4.969	2.425	2.470
	400	372.762±0.470	0.126	–6.810	376.282±3.831	1.018	–5.929
	800	789.892±1.051	0.133	–1.271	790.642±3.315	0.419	–1.169
ononin	375	382.684±0.762	0.199	2.049	384.5343±2.083	0.542	2.542
	750	763.594±0.921	0.121	1.812	758.200±4.755	0.627	1.093
	1500	1507.782±1.069	0.071	0.518	1496.51±9.334	0.623	–0.233

*^*a*^ Mean ± standard deviation (SD). ^*b*^ Relative standard deviation (RSD) % = (SD/mean) × 100. ^*C*^ Accuracy % = [(mean observed concentration − spiked concentration)/spiked concentration] × 100.*

### UPLC Fingerprinting

**FIGURE 6:**
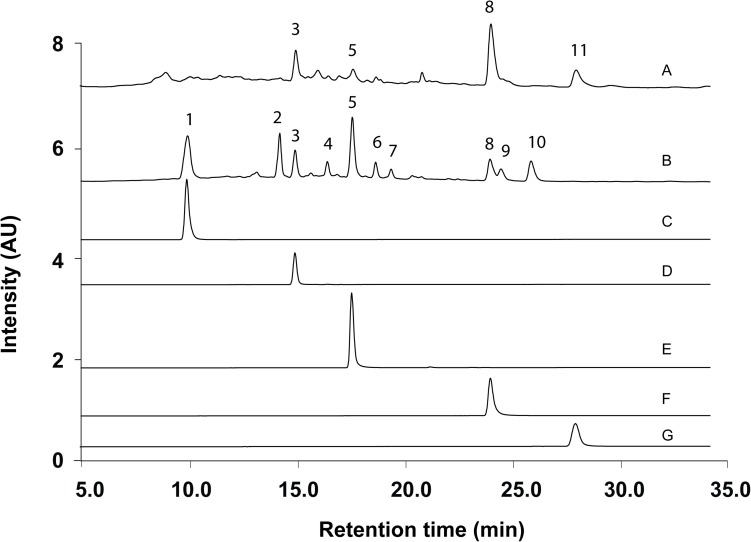
Representative UPLC Chromatograms of samples and standards. (A) Methanolic extract of RH. (B) Methanolic extract of RA. (C) Calycosin-7-*O* -β-D-glucoside. (D) Ononin. (E) Calycosin. (F) Formononetin. (G) Medicarpin.

According to the method and monographs recorded in PPRC (Chinese Pharmacopoeia Commission, 2015) and HKCMMS (Volume I and VIII, Hong Kong), the quality control of RH and RA samples were accessed based on the UPLC analysis of five standard compounds: medicarpin, formononetin, calycosin-7-*O* -β-D-glucoside, calycosin, and ononin. Methanolic extraction of RH and RA samples were injected into UPLC for fingerprinting whereas the same methods were applied for five standard compounds. Figure 6 shows the representative chromatographs of the two herbs and the corresponding peaks of the five standard compounds. The retention times of calycosin-7-*O* -β-D-glucoside, ononin, calycosin, formononetin, and medicarpin were 10.0, 15.0, 17.5, 23.5, and 27.5 min, respectively. The results showed that ononin, calycosin, and formononetin were the common constituents in both species. The major difference between the two species is that calycosin-7-*O* -β-D-glucoside only existed in RA, whereas medicarpin was only found in RH. This finding is consistent with a previous study that showed that formononetin, ononin, calycosin, formononetin-7-*O* -D-glucoside-6^″^-*O* -malonate and soyasaponin are the primary compounds in RH and RA, while medicarpin was unique in RH (Liu et al., 2012).

In the sample preparation stage, CRP fingerprinting requires samples to be extracted in water for 1 h and the crude extracts are subjected to a reverse-phase micro elution 96-well plate for further analysis. In contrast, UPLC analysis contained methanol extraction of samples, dryness, and re-dissolve of the residues which requires 1 day for preparation. Thus, the preparation time for MALDI-TOF MS analysis is 10-fold shorter than UPLC analysis. In addition, the analytical time of MALDI-TOF MS is approximately 5 s, and which is 500-fold faster than UPLC analysis with a running time of 46 min.

### Data Preprocessing

#### Peak Alignment

**FIGURE 7:**
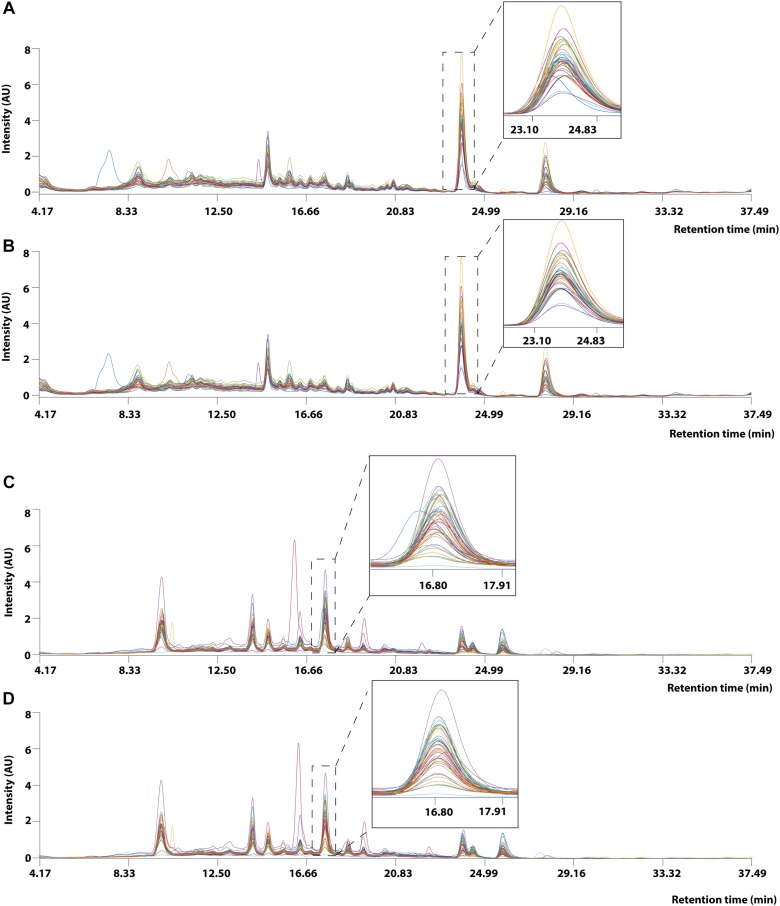
UPLC chromatogram of 40 RH and 51 RA methanolic extracts between retention time between 4.17 and 37.49 min. **(A)** Raw data and **(B)** after Correlation Optimized Warping (COW)-corrected UPLC chromatogram (segment = 148; slack = 1) of RH samples. **(C)** Raw data and **(D)** after Correlation Optimized Warping (COW)-corrected UPLC chromatogram (segment = 149; slack = 22) of RA samples. The chromatograms were zoomed in to show peak alignment after warping.

**FIGURE 8:**
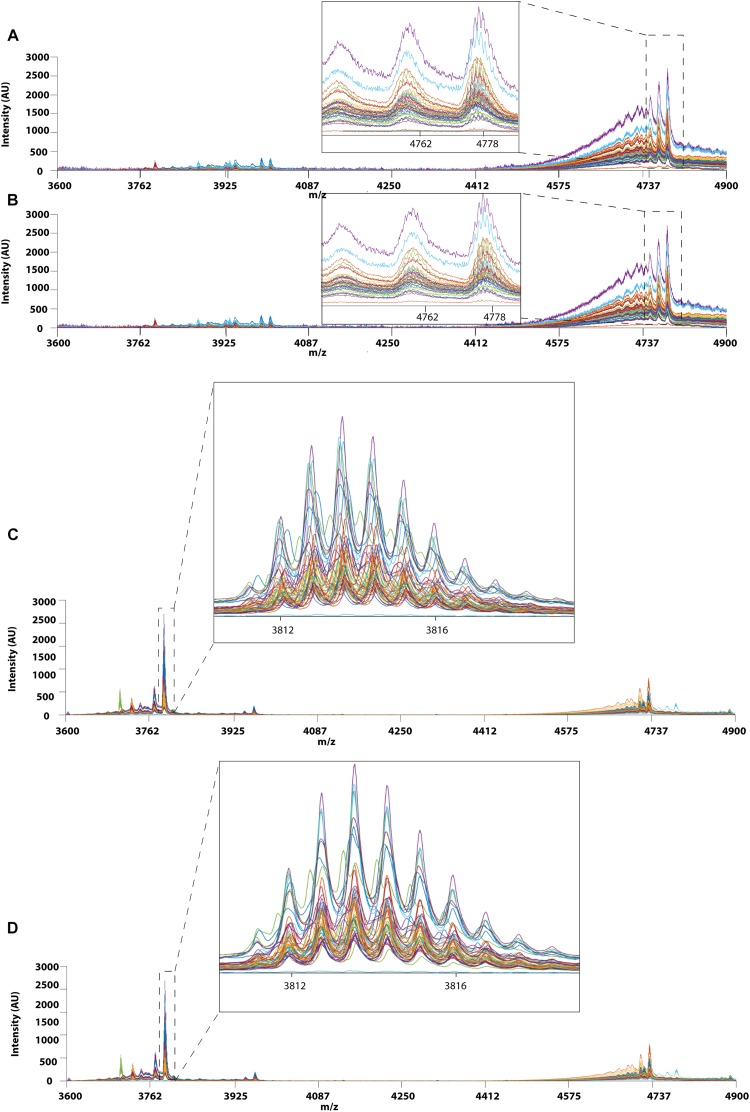
MALDI-TOF MS profiles of 40 RH and 51 RA between 3600 and 4900 Da. **(A)** Raw data and **(B)** after Correlation Optimized Warping (COW)-corrected MALDI-TOF MS spectrum (segment = 150; slack = 5) of RH samples. **(C)** Raw data and **(D)** after Correlation Optimized Warping (COW)-corrected MALDI-TOF MS spectrum (segment = 151; slack = 4) of RA samples. The chromatograms were zoomed in to show peak alignment after warping.

Prior to applying PCA for outlier detection, both MALDI-TOF MS and UPLC data matrices were preprocessed by COW to reduce the noise and inconsistency in the data. Figure 7 shows the chromatograms of forty RH samples and fifty-one RA samples before and after peak alignment whereas Figure 8 shows the MALDI-TOF MS spectra before and after peak alignment of both samples. The reference chromatogram, segment length, and slack numbers are summarized in Supplementary Table S4, which were optimized by the method proposed by Skov et al. (2006).

#### Outliners Detection and Unsupervised Multivariate Analyses

**FIGURE 9:**
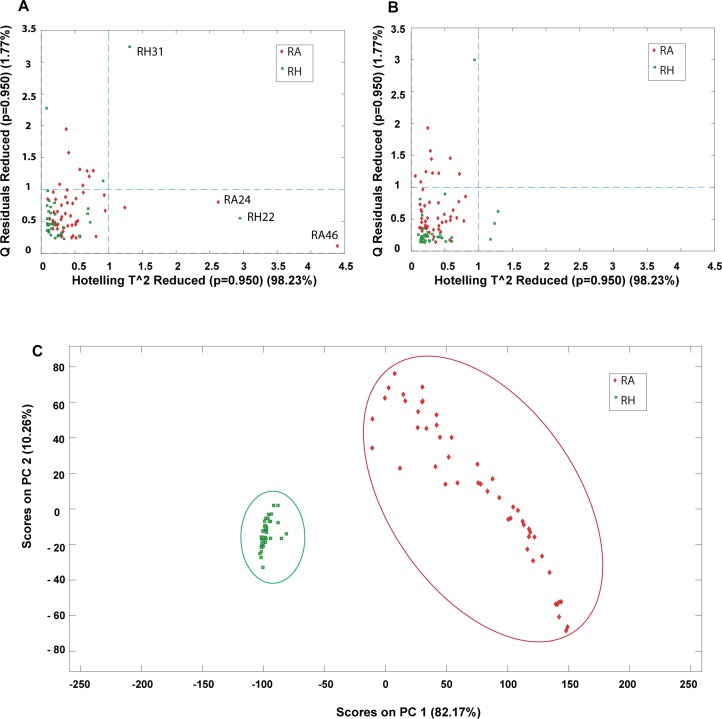
PCA plots obtained from preprocessed MALDI-TOF MS data matrix. Hotelling’s T^2 versus Q residuals plot of **(A)** raw data and **(B)** after removal of outliners. (—-) line represents 95% confidence interval; **(C)** PC1-PC2 scores plot after removing outliners.

Outlier detection is an important evaluation before constructing a classification model since possible anomalous samples in the data matrices could affect the quality of the model and therefore should be removed beforehand. In this study, PCA was applied to identify the presence of outliers and provide an overall idea about the sample distribution. Prior to PCA analysis, the samples were preprocessed by COW baseline removal, standard normal variate and mean centering. The determination of outliners was accessed by Hotelling’s T square verse Q residuals plot, where a sample with high Hotelling’s T square and Q residual value are considered as an outlier. The outlier could donate a greater influence on the model and has a larger variation compared to the projected data and thus should be eliminated before further multivariate analysis. In this study, the MALDI-TOF MS data matrix was used as the main data source for constructing the classification model and thus the outlier detection was applied mainly on the MALDI-TOF MS data. Four outliers (RH22, RH31, RA24, and RA46) were detected from the data matrix and removed for subsequent analysis. Figure 9 shows the Hotelling’s T square versus the Q residuals plot before and after removing the outliners. It can be observed that four outliners are far from the sample major cluster (Figure 9A). In contrast, no samples were detected to have both high Hotelling’s T square and Q residual values after the outliners were eliminated (Figure 9B). Hence, the dataset was reduced to 87 samples, including 38 samples of RH and 49 samples of RA. Figure 9C shows the PC1-PC2 score plots of the preprocessed data after removing the four outliners, showing that RH and RA were well separated into two distinct clusters. The results suggest that these two species have distinct spectrometric characteristics.

**FIGURE 10:**
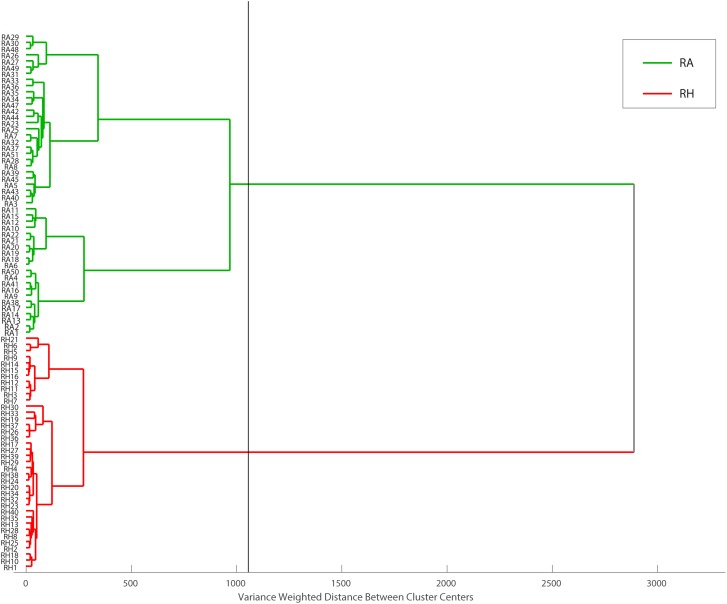
Dendrogram representation of HCA performed on MALDI-TOF data matrix using Euclidean distance and Ward’s method. Obtained data can be divided into two main groups, RH and RA samples at the distance of about 1200 (the black line represents cut-off level).

Hierarchical cluster analysis was performed as a continuation of PCA. With different classification algorithms, it is more promising to obtain sensitive sample classification (Viapiana et al., 2016). In this study, Euclidean distance was checked as a distance similarity measure and Ward’s method was applied. HCA draws a connection between RA and RH, producing a dendrogram (Figure 10) in which similar samples are grouped and this similarity is calculated based on the distance between the samples. The dendrogram showed that all RA samples and RH samples are well separated into two major clusters, highlighted in green and red, respectively. Taken together, our results showed that the clustering pattern obtained using HCA agreed with the classification results acquired from PCA, indicating that the MALDI-TOF MS-based CRP fingerprinting method can deliver a consistent classification result.

#### Optimization of Preprocessing Methods

To improve the data quality and reduce the noise in data matrices, multiple preprocessing algorithms were applied before constructing the multivariate analysis model. Data preprocessing can help to significantly improve the interpretability of the classification models. However, there is no well-established procedure of applying preprocessing algorithms, and as a result, optimization of the preprocessing techniques is needed.

In this study, the data matrices were divided into a calibration set and a validation set based on the Kennard-Stone algorithm. After eliminating the four outliners, 23 RH and 30 RA samples constituted the calibration set, whereas the remaining 15 RH and 19 RA samples were used to generate the validation set. A PLS-DA model was used to compare and evaluate the effect of different preprocessing methods and their respective order on the model’s performance. The data matrices were subjected to mean-centering, standard normal variate, and normalization after the preliminary preprocessing such as peak alignment and baseline correction. Mean-centering aimed at subtracting the column mean from each variable in the respective column. On the other hand, the objective of normalization was to divide each variable by the sum of the absolute value of all variables (Varmuza and Filzmoser, 2016). Furthermore, standard normal variate normalized each chromatogram or spectrum by removing slope variation (Barnes et al., 1989). The effect of different combinations of preprocessing techniques was evaluated by the root mean square error of calibration (RMSEC), root mean square error of cross-validation (RMSECV), root mean square error of prediction (RMSEP), and correlation coefficient from the leave-one-out cross-validation as shown in Table 2.

**TABLE 2:** Comparison of the statistical performance of PLS-DA model after applying various preprocessing methods on the MAIDI calibration and validation data set.

Preprocessing method(s)	**Latent variable (s)^a^**	**RMSEC^b^**	**RMSECV^c^**	**RMSEP^d^**	**Deviation between RMSEP and RMSEC (%)^e^**	**r^2^ cal^f^**	**r^2^ CV^g^**	**r^2^ val^h^**
None	3	0.2260	0.2481	0.1944	–16.2551	0.7966	0.7572	0.7474
standard normal variate + mean centering	2	0.0687	0.0749	0.0671	–2.3845	0.9808	0.9772	0.9837
Normalization + mean centering	2	0.1034	0.1114	0.0938	–10.2345	0.9565	0.9495	0.9654
Mean centering + normalization	2	0.1538	0.1738	0.1469	–4.6971	0.9046	0.8778	0.9206
Mean centering + standard normal variate	2	0.1324	0.1507	0.1518	12.7799	0.9288	0.9076	0.9139

*^*a*^ Optimal number of latent variables; ^*b*^ Root mean square error of calibration; ^*c*^ Root mean square error of cross-validation; ^*d*^ Root mean square error of prediction; ^*e*^ Deviation between RMSEC and RMSEP; ^*f*^ Correlation coefficient of calibration set; ^*g*^ Correlation coefficient of cross-validation set; ^*h*^ Correlation coefficient of validation set.*

The desired preprocessing method combination should have a low complexity (the number of latent variables), a low root mean error, and a high correlation coefficient. According to the results shown in Table 2, preprocessing with standard normal variate followed by mean centering and the PLS-DA model showed the lowest latent variables and the smallest root mean error between the calibration and validation data set. Thus, this combination of preprocessing methods was chosen to be applied to the MALDI-TOF MS and UPLC data matrices in the subsequent analysis.

#### Comparison of Various Classification Models

The classification of RH and RA were constructed using different models, including PLS-DA, KNN, CART, SIMCA, and SVM-DA based on the calibration and validation sets of the preprocessed MALDI-TOF MS and UPLC data matrices. The calibration set (53 samples with 23 RH and 30 RA) was selected by the Kennard-Stone algorithm and used to establish and train the classification model. On the other hand, the validation dataset (34 samples with 15 RH and 19 RA) was employed in the final step to evaluate the predictive ability of the calibrated model.

Tables 3A,B compare different classification models obtained from the preprocessed MALDI-TOF MS and UPLC data matrices. In the confusion matrices, the accuracy in the cross-validation set (CV) and the perdition set (Pred) indicate the interpretability and predictability of each model, respectively. Table 3A illustrates that when the classification model was constructed using the MALDI-TOF MS data matrix, KNN, PLS-DA, and SVM-DA showed the greatest interpretability (100.00%) and predictability (100.00%) for both the cross-validation and prediction data sets, whereas the SIMCA and CART models provided worse performance. SIMCA was shown to have higher interpretability (96.20%) with only one misidentification compared to CART (87.00%), in which 4 RH and 3 RA were misidentified. However, in the prediction set, with 4 (2 RH and 2 RA) samples not assigned, SIMCA showed lower predictability (88.90%) than CART (97.00%). Table 3B summarizes the different classification model results using UPLC data. KNN analysis provided the best interpretability (100.00%) and predictability (100.00%) in both the cross-validation and prediction set. PLS-DA and SVM-DA provided slightly lower interpretability (96.20%), both with one RH sample misidentified as the RA sample. Similar to the results obtained from the MALDI-TOF MS data, SIMCA (89.20%), and CART (96.00%) provided relatively low performance on the cross-validation set. With 5 (2 RH and 3 RA) samples not assigned, SIMCA delivered the worse interpretability. However, all models showed 100.00% predictability on the prediction set, which indicated the high prediction ability of the UPLC data matrix using different classification models.

**TABLE 3A:** The confusion matrices obtained from the prediction set of the preprocessed MALDI-TOF MS data.

	**CV^a^**						**Pred^b^**				
		
			**Predicted class**					**Predicted class**			
									
	**True class**	**N^c^**	**RH**	**RA**	**NA^d^**	**Accuracy (%)**	**N**	**RH**	**RA**	**NA**	**Accuracy (%)**
KNN	RH	23	23	−	−	100.00	15	15	−	−	100.00
	RA	30	−	30	−		19	−	19	−	
PLS-DA	RH	23	23	−	−	100.00	15	15	−	−	100.00
	RA	30	−	30	−		19	−	19	−	
SIMCA	RH	23	23	−	−	96.20	15	13	−	2	88.90
	RA	30	−	29	1		19	−	17	2	
SVM-DA	RH	23	23	−	−	100.00	15	15	−	−	100.00
	RA	30	−	30	−		19	−	19	−	
CART	RH	23	19	4	−	87.00	15	14	1	−	97.00
	RA	30	3	27	−		19	−	19	−	

*^*a*^ Venetian blind cross-validation set; ^*b*^ Prediction set; ^*c*^ Number of samples; ^*d*^ Not assigned.*

**TABLE 3B:** The confusion matrices obtained from the prediction set of the preprocessed UPLC data.

	**CV^a^**						**Pred^b^**					
			
			**Predicted class**						**Predicted class**			
								
	**True class**	**N^c^**	**RH**	**RA**	**NA^d^**	**Accuracy (%)**		**N**	**RH**	**RA**	**NA**	**Accuracy (%)**
KNN	RH	23	23	−	−	100.00		15	15	−	−	100.00
	RA	30	−	30	−			19	−	19	−	
PLS-DA	RH	23	22	1	−	96.20		15	15	−	−	100.00
	RA	30	−	30	−			19	−	19	−	
SIMCA	RH	23	21	−	2	89.20		15	15	−	−	100.00
	RA	30	−	27	3			19	−	19	−	
SVM-DA	RH	23	22	1	−	96.20		15	15	−	−	100.00
	RA	30	−	30	−			19	−	19	−	
CART	RH	23	22	1	−	96.00		15	15	−	−	100.00
	RA	30	1	29	−			19	−	19	−	

*^*a*^ Venetian blind cross-validation set; ^*b*^ Prediction set; ^*c*^ Number of samples; ^*d*^ Not assigned.*

Generally, HPLC or UPLC data will provide a relatively high prediction ability of classification performance compared with data obtained from other analytical instruments (Liu et al., 2010). Thus, it is not surprising that our study provided consistent results as illustrated in Table 3. UPLC generated a perfect performance on various classification models in that all RH and RA samples were correctly categorized in the prediction set. Interestingly, the classification models constructed using the MALDI-TOF MS data matrix showed comparable classification ability to the UPLC data matrix, with prediction accuracies more than 89.00% in all models. The results suggested that MALDI-TOF MS can also be applied as a reliable alternative analytical technique in differentiation samples.

For a better understanding of the classification ability of different models, sensitivity, specificity, the error rate (ER) and the non-error rate (NER) of each model on the prediction set was calculated and compared (Table 3C). Basically, the four parameters showed the ability to correctly classify samples belonging to a specific class, and the ability to reject the samples from all other classes of a classification model. Similar to the illustration in the confusion matrix, all models showed a perfect score for one for the UPLC data, suggesting that all models were able to correctly classify the samples based on UPLC data matrices. For the MALDI-TOF MS data, except for the SIMCA and CART model, all of the other models showed perfect classification abilities as well. The low sensitivity value in SIMCA (0.89) suggested that the model is more preferable in discriminating RH than RA. In contrast, the low specificity value in CART (0.93) indicated the greater ability of this model to differentiate RA than RH. However, the overall performance of all models based on the MALDI-TOF MS data was relatively high, suggesting that MALDI-TOF MS coupled with multivariate classification analyses is reliable for sample classification.

**TABLE 3C:** The classification parameters of the preprocessed UPLC and MALDI-TOF MS data obtained from the prediction set.

**MALDI-TOF MS**					**UPLC**			
	
			**RA**				**RA**	
						
	**ER^a^**	**NER^b^**	**Specificity**	**Sensitivity**	**ER^a^**	**NER^b^**	**Specificity**	**Sensitivity**
KNN	0	1	1	1	0	1	1	1
PLS-DA	0	1	1	1	0	1	1	1
SIMCA	0.06	0.94	1	0.89	0	1	1	1
SVM-DA	0	1	1	1	0	1	1	1
CART	0.03	0.97	0.93	1	0	1	1	1

*^*a*^ Error rate; ^*b*^ Non-error rate.*

Previous studies on evaluating the performance of different classification models also showed similar results. The differentiation of Puerariae Lobatae Radix and Puerariae Thomsonii Radix using HTPLC coupled with a seven classification model showed that the SIMCA model delivered the worst performance, with a 0.5 error rate and 60.00% accuracy in predicting the class of samples, compared to KNN, PLS-DA, and SVM-DA (Wong et al., 2014). Additionally, it also showed that CART performed less well than the other classification models with its low sensitivity value (0.38) and its low prediction rate (64.29%). Another study on the characterization of transgenic and non-transgenic soybean oil using NIR spectroscopy conducted by Luna et al. (2013) demonstrated similar findings. This study showed that the best classification results were provided by SVM-DA (CV: 100.00%, Pred: 95.00%) and PLS-DA (CV: 97.50%, Pred: 90.00%). In contrast, SIMCA provided lower results in class modeling. However, not all studies showed the same results. For example, a study conducted by Martins showed that SIMCA exhibited only a 12.00% correct rate when differentiating *Phyllanthus* species using HPLC. However, 100.00% of the samples were correctly classified while using a SIMCA model based on NIR data, whereas the PLS-DA and KNN model only showed an 80.00% accuracy (Martins et al., 2011). Overall, it can be revealed that the ability of a classification model might not be the same when applying different analytical methods.

Here, KNN showed the greatest classification performance and was the most preferable algorithm to differentiate RH and RA since it required minimal data handling procedures and the shortest running time. However, PLS-DA and SVM-DA are more suitable when the study is focused on the distribution of the classes and the relationships between the variables. Additionally, with slightly lower specificity and accuracy, CART is less favorable compared to KNN, PLS-DA, and SVM-DA. Among all the classification models, SIMCA is the least preferable method for distinguishing RH and RA because of its lowest accuracy in prediction and requirements in multiple steps for data optimization.

## Conclusion

Traditionally, small-molecule metabolites such as saponins and flavonoids quantified by chromatographic analysis is employed to differentiate herbs and herbal products. In this study, we report CRP fingerprinting as a general method for herbal authentication based on the hyperstable CRPs with molecular weights ranging from 2 and 6 kDa. The usefulness of CRP fingerprinting was validated in screening 100 herbs and herbal products. CRP fingerprinting produces consistent results regardless of the morphology, chemical composition, and origins of the herbs and herbal products. This method is also useful to authenticate key ingredients in complex formulation Shu-Jing-Huo-Xue-Tang, which contains 17 herbs. In particular, we identified the novel achyranthes aB1 as a useful CRP marker to authenticate this complex formulation. In addition, CRP fingerprinting coulpled with multivariate analyses was employed to differentiate RA from its closely related species RH. Using the MALDI-TOF MS technique, we showed that astratides aM1 and aM2 are the unique CRPs present in RA while hedytides hP1 and hP2 are novel CRPs that only found in RH species. *De novo* sequencing revealed that astratides and hedytides are different in amino acid composition. Compared to the conventional quality control method using chromatographic fingerprinting, CRP fingerprinting based on MALDI-TOF MS analysis is 500-fold faster. Unsupervised multivariate analyses such as PCA and HCA showed that RA and RH can be separated into two clusters based on their CRP fingerprints. In addition, the classification ability of CRP fingerprinting coupled with five supervised multivariate analyses had comparable classification accuracy to that of UPLC. In terms of the performance of classification models, KNN, PLS-DA, and SVM-DA from CRP fingerprinting showed a perfect correct classification rate (100.00%) while minor classification errors (3.00%) were found in the CART model. With 88.90% sensitivity and 94.00% correct rate of classification, SIMCA performed the worse and thus became the least preferable classification model. Overall, with simple handling procedure and accurate classification results, CRP fingerprinting can be used as a novel and general approach for quality control and authentication of herbal and herbal products.

## Data Availability

The MALDI-TOF MS data set analyzed for this study can be found in MassIVE repository (doi: 10.25345/C5WH14). The raw data supporting the conclusions of this manuscript will be made available by the authors, without undue reservation, to any qualified researcher.

## Author Contributions

JT, JH, and KW conceived and designed the experiments. JH, KW, ST, AH, and JT performed the experiments, analyzed the data, and wrote the manuscript. JT revised the manuscript. All authors read and approved the final version of the manuscript.

## Conflict of Interest Statement

The authors declare that the research was conducted in the absence of any commercial or financial relationships that could be construed as a potential conflict of interest.
