# Supplementary material for: Cysteine-Rich Peptide Fingerprinting as a General Method for Herbal Analysis to Differentiate Radix Astragali and Radix Hedysarum
Source: Front Plant Sci. 2019 Jul 31;10:973. doi: 10.3389/fpls.2019.00973 (PMC6684776; doi:10.3389/fpls.2019.00973)
Supplement: Supplementary file 1 [file Data_Sheet_1.docx]

Supplementary materials for

**Cysteine-rich peptide fingerprinting as a general method for herbal analysis to differentiate Radix Astragali and Radix Hedysarum**

Jiayi Huang, Ka H. Wong, Stephanie V. Tay, Adrian How and James P. Tam*

School of Biological Sciences, Nanyang Technological University, Singapore, Singapore

***Correspondence:**

Professor James P. Tam

[jptam@ntu.edu.sg]

**Supplementary Table S1.** List of plants and their extraction methods

| \| **Scientific name** \| **Form** \| **Extraction method** \| \| --- \| --- \| --- \| \| *Abrus precatorius* \| Dried herb \| 50% ethanol \| \| *Abutilon indicum* \| Fresh plant \| 50% ethanol \| \| *Achyranthes aspera* \| Fresh plant \| 50% ethanol \| \| *Achyranthes bidentate* \| Dried herb \| water \| \| *Alternanthera ficoidea* \| Fresh plant \| 50% ethanol \| \| *Alternanthera paronychiodes* \| Fresh plant \| 50% ethanol \| \| *Alternanthera sessilis* \| Fresh plant \| 50% ethanol \| \| *Angelica sinensis* \| Granules \| water \| \| *Armeniacae amarum* \| Granules \| water \| \| *Artemisia argyi* \| Fresh plant \| water \| \| *Brassica alboglabra* \| Fresh plant \| 50% ethanol \| \| *Brucea javanica* \| Dried herb \| water \| \| *Calandrinia umbellata* \| Fresh plant \| 50% ethanol \| \| *Camellia sinensis* \| Dried herb \| water \| \| *Cannabis sativa* \| Dried herb \| water \| \| *Cassia occidentalis* \| Fresh plant \| 50% ethanol \| \| *Celosia argentea* \| Dried herb \| 50% ethanol \| \| *Cerbera odollam* \| Fresh plant \| 50% ethanol \| \| *Citrus aurantium* \| Fresh plant \| water \| \| *Clematis chinensis* \| Dried herb \| 50% ethanol \| \| *Clitoria ternatea* \| Fresh plant \| water \| \| *Codonopsis pilosula* \| Dried herb \| 50% ethanol \| \| *Coix lacryma-jobi* \| Fresh plant \| water \| \| *Curcuma alba* \| Fresh plant \| water \| \| *Cuscuta chinensis* \| Granules \| water \| \| *Cyathula officinalis* \| Dried herb \| 50% ethanol \| \| *Dichondra repens* \| Fresh plant \| 50% ethanol \| \| *Dioscorea polystachya* \| Dried herb \| 50% ethanol \| \| *Dolichos lablab* \| Dried herb \| 50% ethanol \| \| *Drynaria fortunei* \| Granules \| 50% ethanol \| \| *Eleutherococcus senticousus* \| Dried Herb \| Water \| \| *Ephedra sinica* \| Dried herb \| water \| \| *Euchresta japonica* \| Dried herb \| 50% ethanol \| \| *Eucommia ulmoides* \| Granules \| water \| \| *Foeniculum vulgare* \| Fresh plant \| water \| \| *Folium ginseng* \| Dried herb \| 50% ethanol \| \| *Ganoderma lucidum* \| Dried herb \| water \| \| *Ginkgo biloba* \| Dried herb \| 50% ethanol \| \| *Glycine max* \| Fresh plant \| 50% ethanol \| \| *Gomphrena globose* \| Fresh plant \| 50% ethanol \| \| *Gynostemma pentaphyllum* \| Dried herb \| 50% ethanol \| \| *Heduotis chrysotricha* \| Fresh plant \| 50% ethanol \| \| *Hedyotis auricularia* \| Fresh plant \| 50% ethanol \| \| *Hedyotis biflora* \| Fresh plant \| 50% ethanol \| \| *Hedyotis congesta* \| Fresh plant \| 50% ethanol \| \| *Hedyotis corymbosa* \| Fresh plant \| 50% ethanol \| \| *Hedyotis dichotoma* \| Fresh plant \| 50% ethanol \| \| *Hedyotis microphylla (Ariel)* \| Fresh plant \| 50% ethanol \| \| *Hedyotis microphylla (Roots)* \| Fresh plant \| 50% ethanol \| \| *Hibiscus sabdariffa* \| Fresh plant \| 50% ethanol \| \| *Hordei germinates* \| Granules \| water \| \| *Impatiens balsamina* \| Dried herb \| 50% ethanol \| \| *Jasminum grandiflorum* \| Dried herb \| 50% ethanol \| \| *Lasianthus tomentosus* \| Fresh plant \| water \| \| *Ligusticum wallichii* \| Granules \| water \| \| *Lilium brownii* \| Granules \| water \| \| *Liquidambar formosana* \| Dried herb \| water \| \| *Lycium barbarum* \| Dried herb \| 50% ethanol \| \| *Lycium chinense* \| Dried herb \| 50% ethanol \| \| *Millettia speciosa* \| Dried herb \| water \| \| *Momordica charantia* \| Fresh plant \| water \| \| *Morinda officinalis* \| Granules \| water \| \| *Morus alba* \| Dried herb \| 50% ethanol \| \| *Nelumbo nucifera* \| Dried herb \| 50% ethanol \| \| *Oldenlandia affinis* \| Fresh plant \| water \| \| *Oryza sativa* \| Dried herb \| 50% ethanol \| \| *Oxalis corniculata* \| Fresh plant \| 50% ethanol \| \| *Panax ginseng* \| Dried herb \| 50% ethanol \| \| *Panax notoginseng* \| Dried herb \| 50% ethanol \| \| *Panax quinquefolius* \| Dried herb \| 50% ethanol \| \| *Pereskia bleo* \| Fresh plant \| 50% ethanol \| \| *Perilla frutescens* \| Dried herb \| 50% ethanol \| \| *Phaseolus vulgaris* \| Fresh plant \| 50% ethanol \| \| *Phragmite communis* \| Dried herb \| 50% ethanol \| \| *Plantago asiatica* \| Granules \| 50% ethanol \| \| *Platycladus orientalis* \| Dried herb \| 50% ethanol \| \| *Polygonum cuspidatum* \| Fresh plant \| 50% ethanol \| \| *Polygonum multiflorum* \| Dried herb \| 50% ethanol \| \| *Portulaca grandiflora* \| Fresh plant \| 50% ethanol \| \| *Portulaca oleracea* \| Fresh plant \| 50% ethanol \| \| *Portulaca pilosa* \| Fresh plant \| 50% ethanol \| \| *Prinsepia uniflora* \| Dried herb \| water \| \| *Prunella vulgaris* \| Fresh plant \| 50% ethanol \| \| *Pruni Semen* \| Dried herb \| water \| \| *Prunus persica* \| Dried herb \| 50% ethanol \| \| *Pseudostellaria heterophylla* \| Dried herb \| water \| \| *Psoralea corylifolia* \| Granules \| water \| \| *Setaria italic* \| Dried herb \| 50% ethanol \| \| *Smilax glabra* \| Granules \| water \| \| *Talinum triangulare* \| Fresh plant \| 50% ethanol \| \| *Taraxacum mongolicum* \| Granules \| 50% ethanol \| \| *Trichosanthes kirilowii* \| Dried herb \| 50% ethanol \| \| *Tripterygium wilfordii* \| Dried herb \| water \| \| *Typhonium divaricatum* \| Fresh plant \| 50% ethanol \| \| *Vernonia cinerea* \| Fresh plant \| 50% ethanol \| \| *Vigna umbellate* \| Dried herb \| 50% ethanol \| \| *Viola yedoensis* \| Granules \| water \| \| *Zea mays* \| Dried herb \| 50% ethanol \| \| *Zingiber officinale* \| Fresh plant \| 50% ethanol \| \| *Ziziphus jujuba* \| Dried herb \| 50% ethanol \| |
| --- | --- | --- | --- | --- | --- | --- | --- | --- | --- | --- | --- | --- | --- | --- | --- | --- | --- | --- | --- | --- | --- | --- | --- | --- | --- | --- | --- | --- | --- | --- | --- | --- | --- | --- | --- | --- | --- | --- | --- | --- | --- | --- | --- | --- | --- | --- | --- | --- | --- | --- | --- | --- | --- | --- | --- | --- | --- | --- | --- | --- | --- | --- | --- | --- | --- | --- | --- | --- | --- | --- | --- | --- | --- | --- | --- | --- | --- | --- | --- | --- | --- | --- | --- | --- | --- | --- | --- | --- | --- | --- | --- | --- | --- | --- | --- | --- | --- | --- | --- | --- | --- | --- | --- | --- | --- | --- | --- | --- | --- | --- | --- | --- | --- | --- | --- | --- | --- | --- | --- | --- | --- | --- | --- | --- | --- | --- | --- | --- | --- | --- | --- | --- | --- | --- | --- | --- | --- | --- | --- | --- | --- | --- | --- | --- | --- | --- | --- | --- | --- | --- | --- | --- | --- | --- | --- | --- | --- | --- | --- | --- | --- | --- | --- | --- | --- | --- | --- | --- | --- | --- | --- | --- | --- | --- | --- | --- | --- | --- | --- | --- | --- | --- | --- | --- | --- | --- | --- | --- | --- | --- | --- | --- | --- | --- | --- | --- | --- | --- | --- | --- | --- | --- | --- | --- | --- | --- | --- | --- | --- | --- | --- | --- | --- | --- | --- | --- | --- | --- | --- | --- | --- | --- | --- | --- | --- | --- | --- | --- | --- | --- | --- | --- | --- | --- | --- | --- | --- | --- | --- | --- | --- | --- | --- | --- | --- | --- | --- | --- | --- | --- | --- | --- | --- | --- | --- | --- | --- | --- | --- | --- | --- | --- | --- | --- | --- | --- | --- | --- | --- | --- | --- | --- | --- | --- | --- | --- | --- | --- | --- | --- | --- | --- | --- | --- | --- | --- | --- | --- | --- | --- | --- | --- | --- | --- | --- | --- | --- | --- | --- | --- | --- | --- | --- |
|  |
|  |
|  |
|  |
|  |
|  |
|  |

**Supplementary Table S2.** The sample code and collection location of the RH and RA samples.

| *H. polybotrys* |  |  | *A. membranaceus* |  |
| --- | --- | --- | --- | --- |
| Sample code | Collection location |  | Sample code | Collection location |
| RH1 | Gansu, China |  | RA1 | Anhui, China |
| RH2 | Gansu, China |  | RA2 | Anhui, China |
| RH3 | Gansu, China |  | RA3 | Anhui, China |
| RH4 | Gansu, China |  | RA4 | Anhui, China |
| RH5 | Guangdong, China |  | RA5 | Anhui, China |
| RH6 | Gansu, China |  | RA6 | Anhui, China |
| RH7 | Gansu, China |  | RA7 | Jiangsu, China |
| RH8 | Anhui, China |  | RA8 | Anhui, China |
| RH9 | Gansu, China |  | RA9 | Anhui, China |
| RH10 | Jiangsu, China |  | RA10 | Anhui, China |
| RH11 | Guangdong, China |  | RA11 | Anhui, China |
| RH12 | Gansu, China |  | RA12 | Anhui, China |
| RH13 | Anhui, China |  | RA13 | Anhui, China |
| RH14 | Gansu, China |  | RA14 | Jiangsu, China |
| RH15 | Guangdong, China |  | RA15 | Gansu, China |
| RH16 | Anhui, China |  | RA16 | Gansu, China |
| RH17 | Guangdong, China |  | RA17 | Gansu, China |
| RH18 | Gansu, China |  | RA18 | Gansu, China |
| RH19 | Gansu, China |  | RA19 | Jiangsu, China |
| RH20 | Gansu, China |  | RA20 | Inner Mongolia, China |
| RH21 | Inner Mongolia, China |  | RA21 | Guangdong, China |
| RH22 | Gansu, China |  | RA22 | Hong Kong, China |
| RH23 | Gansu, China |  | RA23 | Gansu, China |
| RH24 | Anhui, China |  | RA24 | Guangdong, China |
| RH25 | Jiangsu, China |  | RA25 | Guangdong, China |
| RH26 | Shanxi, China |  | RA26 | Shanxi, China |
| RH27 | Guangdong, China |  | RA27 | Heilongjiang. China |
| RH28 | Guangdong, China |  | RA28 | Inner Mongolia, China |
| RH29 | Sichuan, China |  | RA29 | Ningxia, China |
| RH30 | Sichuan, China |  | RA30 | Beijing, China |
| RH31 | Beijing, China |  | RA31 | Yunnan, China |
| RH32 | Jiangxi, China, |  | RA32 | Yunnan, China |
| RH33 | Zhejiang, China |  | RA33 | Shangdong, China |
| RH34 | Hong Kong, China |  | RA34 | Qinghai, China |
| RH35 | Hebei, China |  | RA35 | Shanxi, China |
| RH36 | Hebei, China |  | RA36 | Shanxi, China |
| RH37 | Hubei, China |  | RA37 | Shanxi, China |
| RH38 | Hong Kong, China |  | RA38 | Jilin, China |
| RH39 | Shanghai, China |  | RA39 | Jilin, China |
| RH40 | Gansu, China |  | RA40 | Jilin, China |
|  |  |  | RA41 | Jilin, China |
|  |  |  | RA42 | Fujian, China |
|  |  |  | RA43 | Sichuan, China |
|  |  |  | RA44 | Sichuan, China |
|  |  |  | RA45 | Hebei, china |
|  |  |  | RA46 | Xinjiang, China |
|  |  |  | RA47 | Xinjiang, China |
|  |  |  | RA48 | Xinjiang, China |
|  |  |  | RA49 | Liaoning, China |
|  |  |  | RA50 | Liaoning, China |
|  |  |  | RA51 | Singapore |

**Supplementary Table S3.** The calibration curve parameters, LOD, LOQ of five standard compounds.

|  | Linear range (µg/mL) | Correlation  Coefficient (r^2^) | Slope | y-intercept | LOD (µg/mL) | LOQ (µg/mL) |
| --- | --- | --- | --- | --- | --- | --- |
| calycosin | 0.02-800 | 0.9990 | 51896 | 165802 | 0.01 | 0.04 |
| calycosin-7-O-beta-D-glucoside | 0.24-1200 | 0.9990 | 39371 | -18677 | 0.11 | 0.32 |
| medicarpin | 0.49-1000 | 0.9990 | 28886 | 8705 | 0.14 | 0.43 |
| ononin | 0.24-3000 | 0.9993 | 23079 | 389359 | 0.10 | 0.31 |
| formononetin | 0.06-250 | 0.9991 | 45118 | 10206 | 0.02 | 0.07 |

**Supplementary Table S4.** COW pre-processing parameters and reference chromatograms or mass spectrum for UPLC and MALDI-TOF MS data matrices.

| Data matrices | Sample | Segment length | Slack number | Reference chromatograms or mass spectrums |
| --- | --- | --- | --- | --- |
| UPLC | *H. polybotrys* | 148 | 1 | RH15 |
| UPLC | *A. membranaceus* | 149 | 22 | RA3 |
| MALDI-TOF MS | *H. polybotrys* | 150 | 5 | RH28 |
| MALDI-TOF MS | *A. membranaceus* | 151 | 4 | RA40 |

**
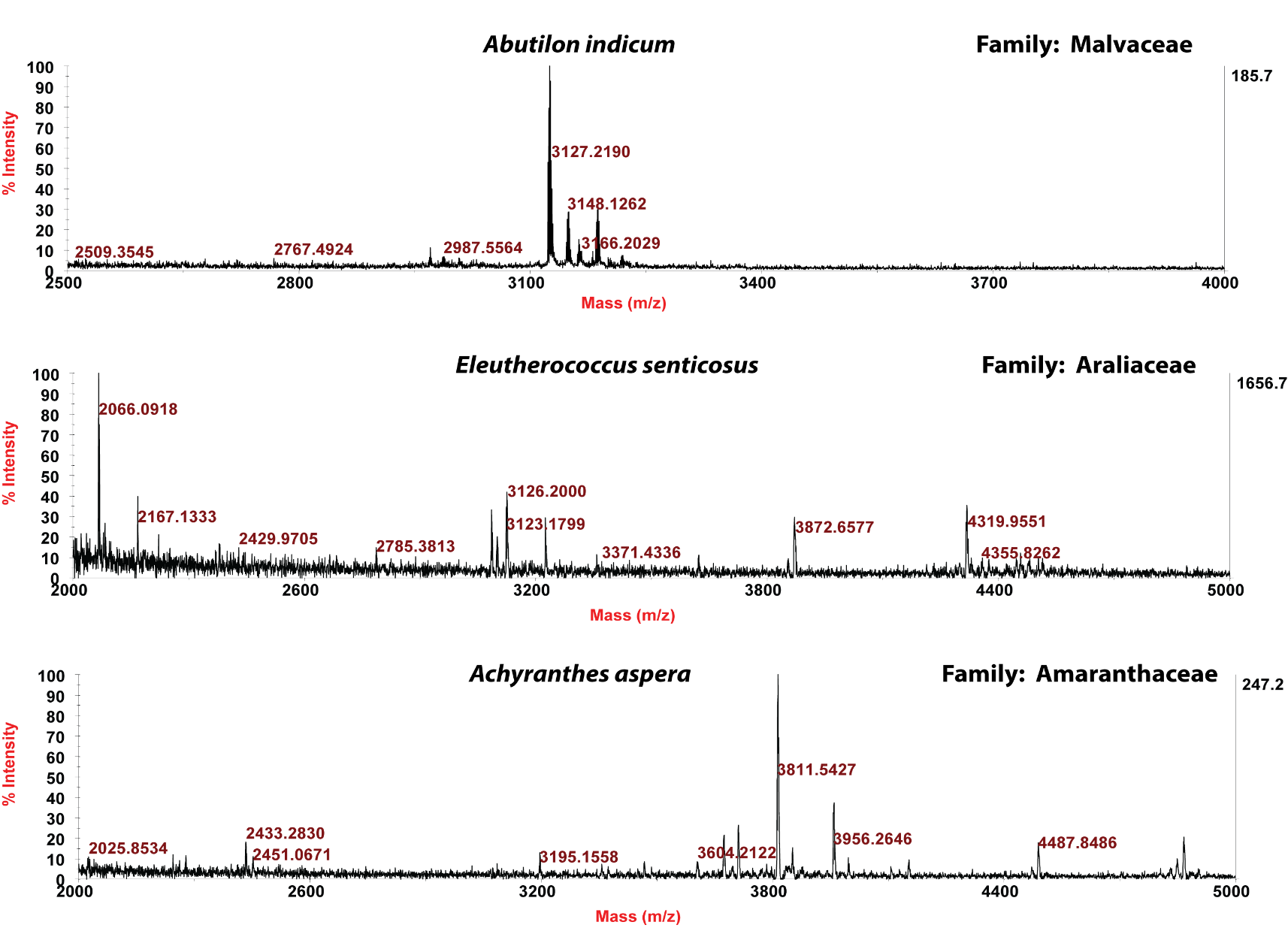

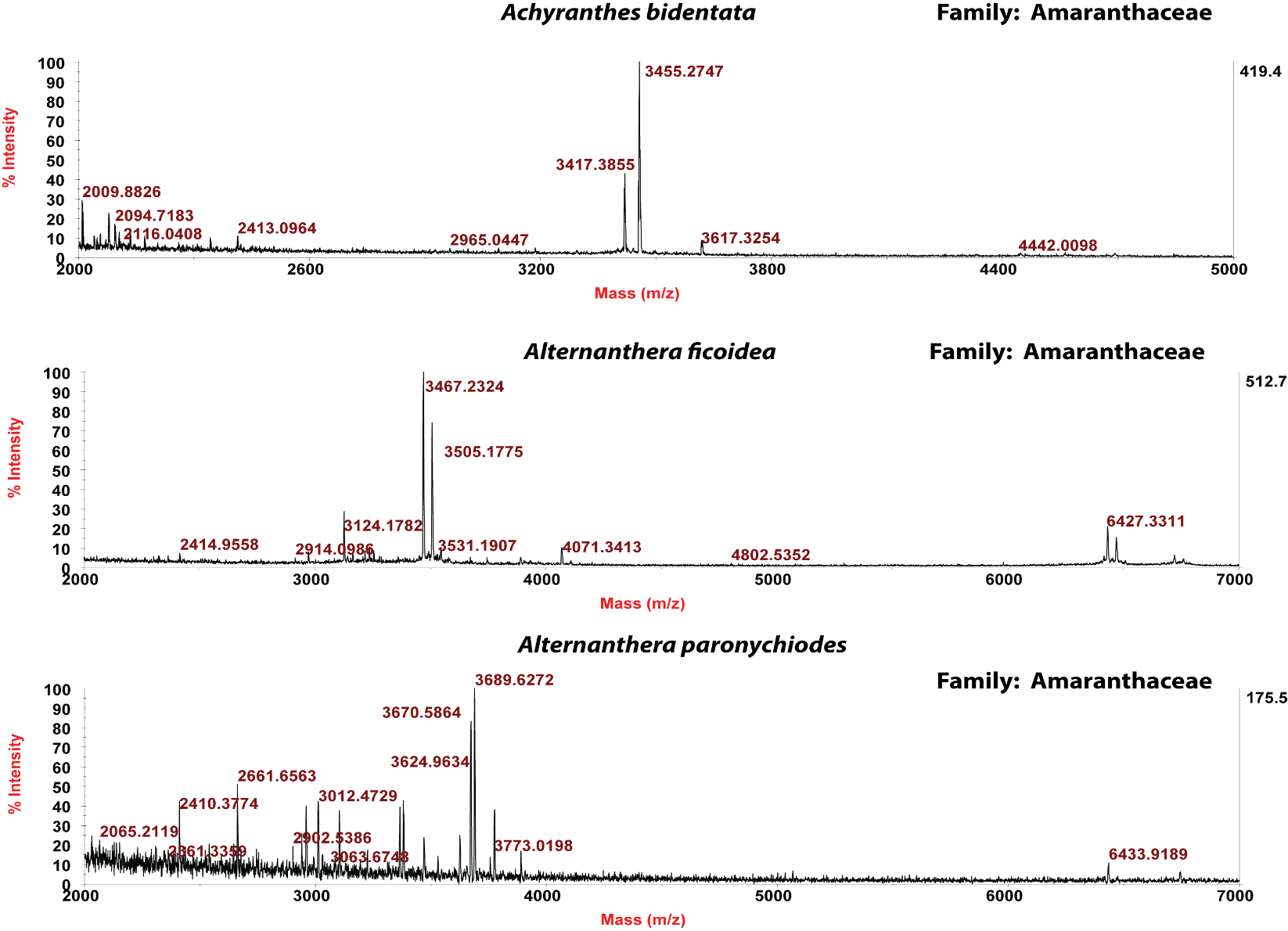

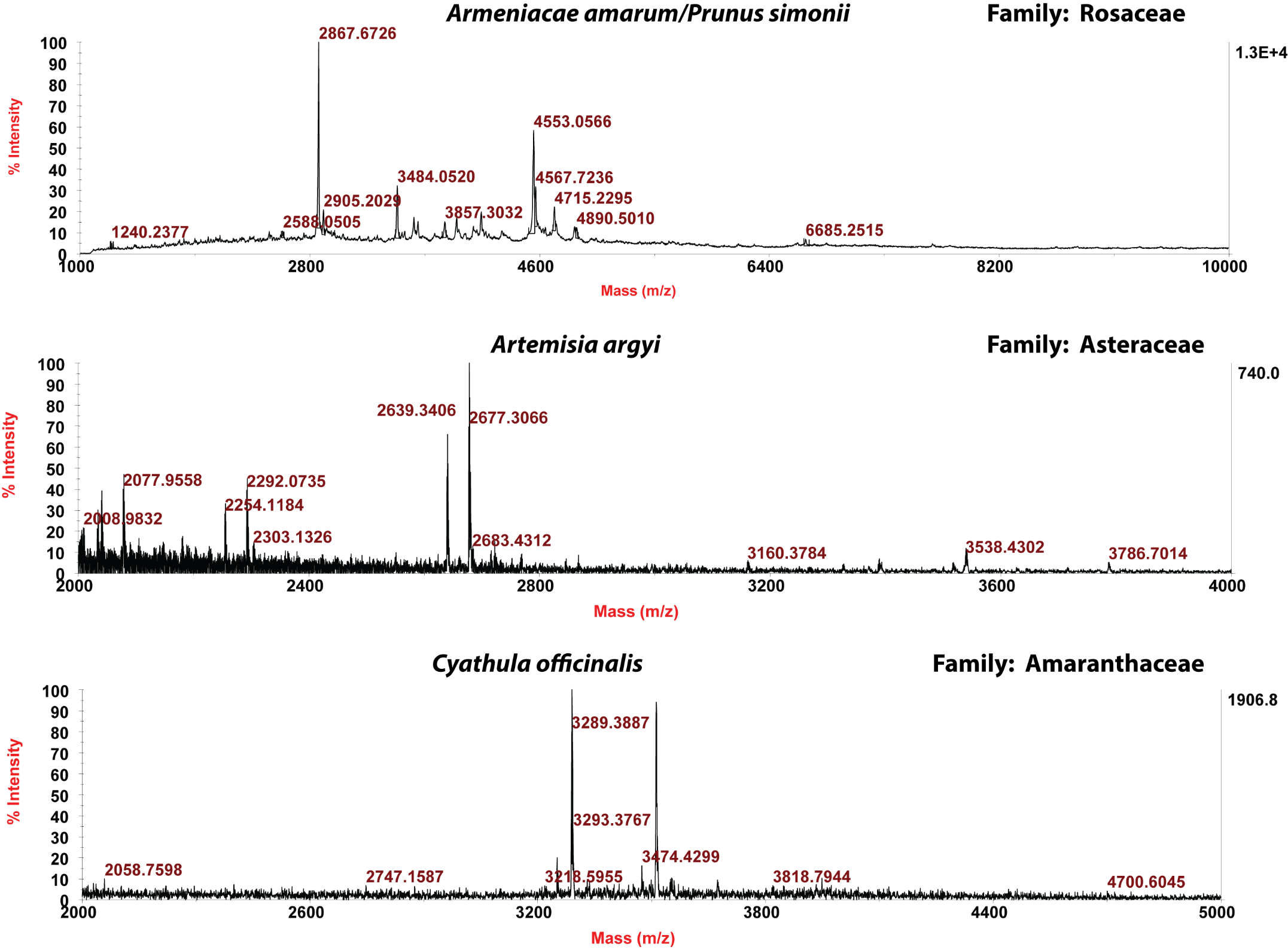

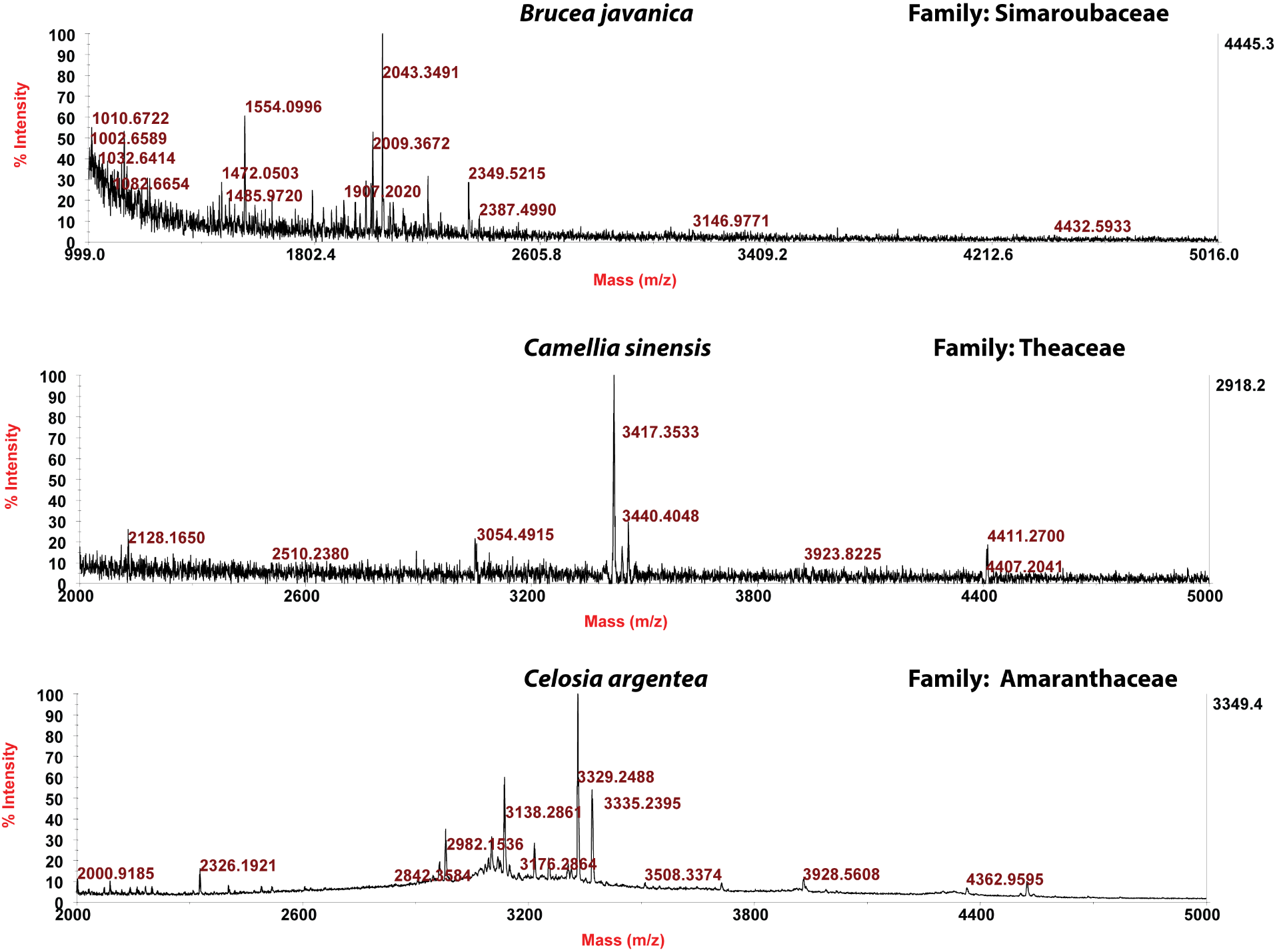

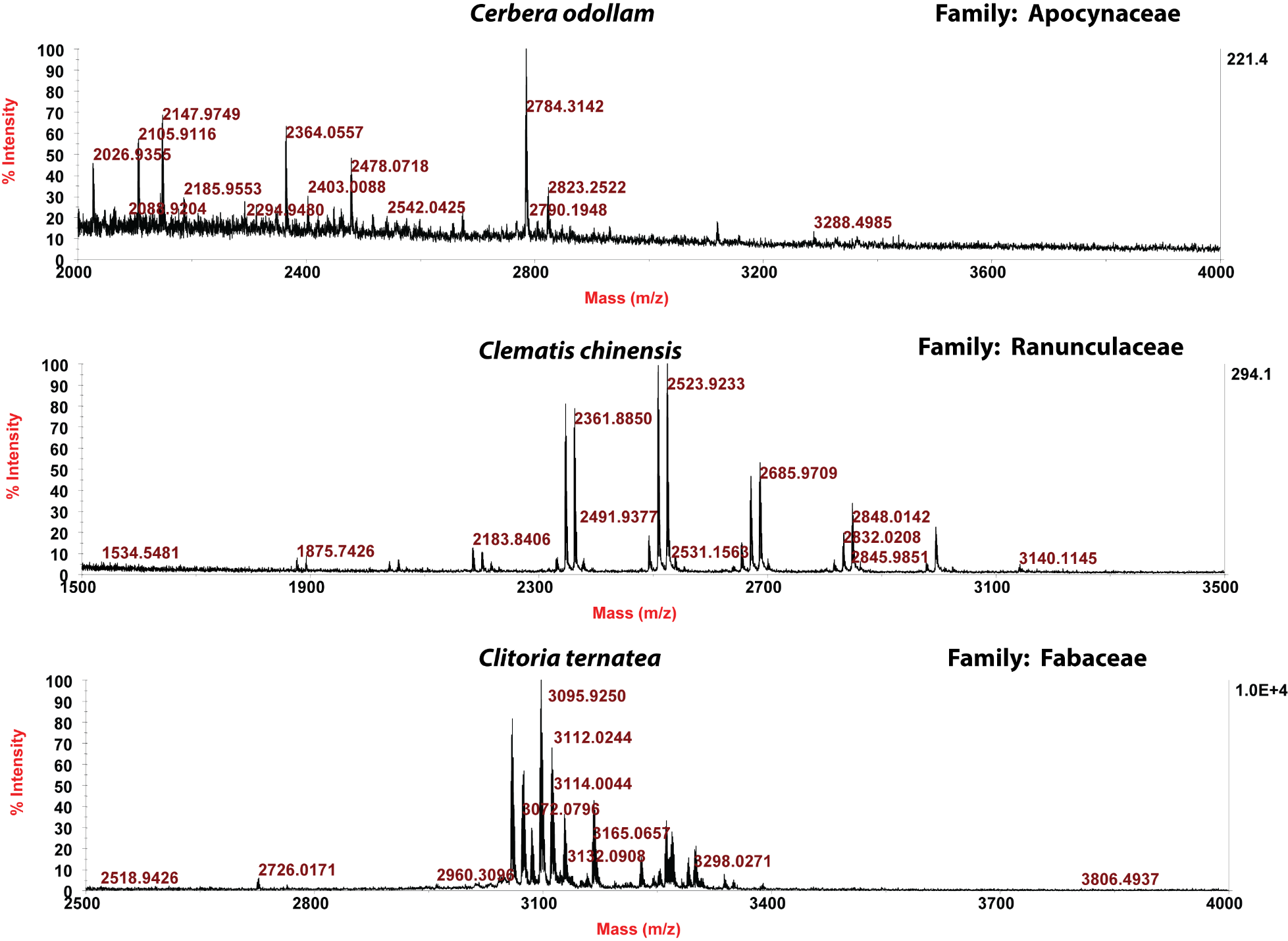

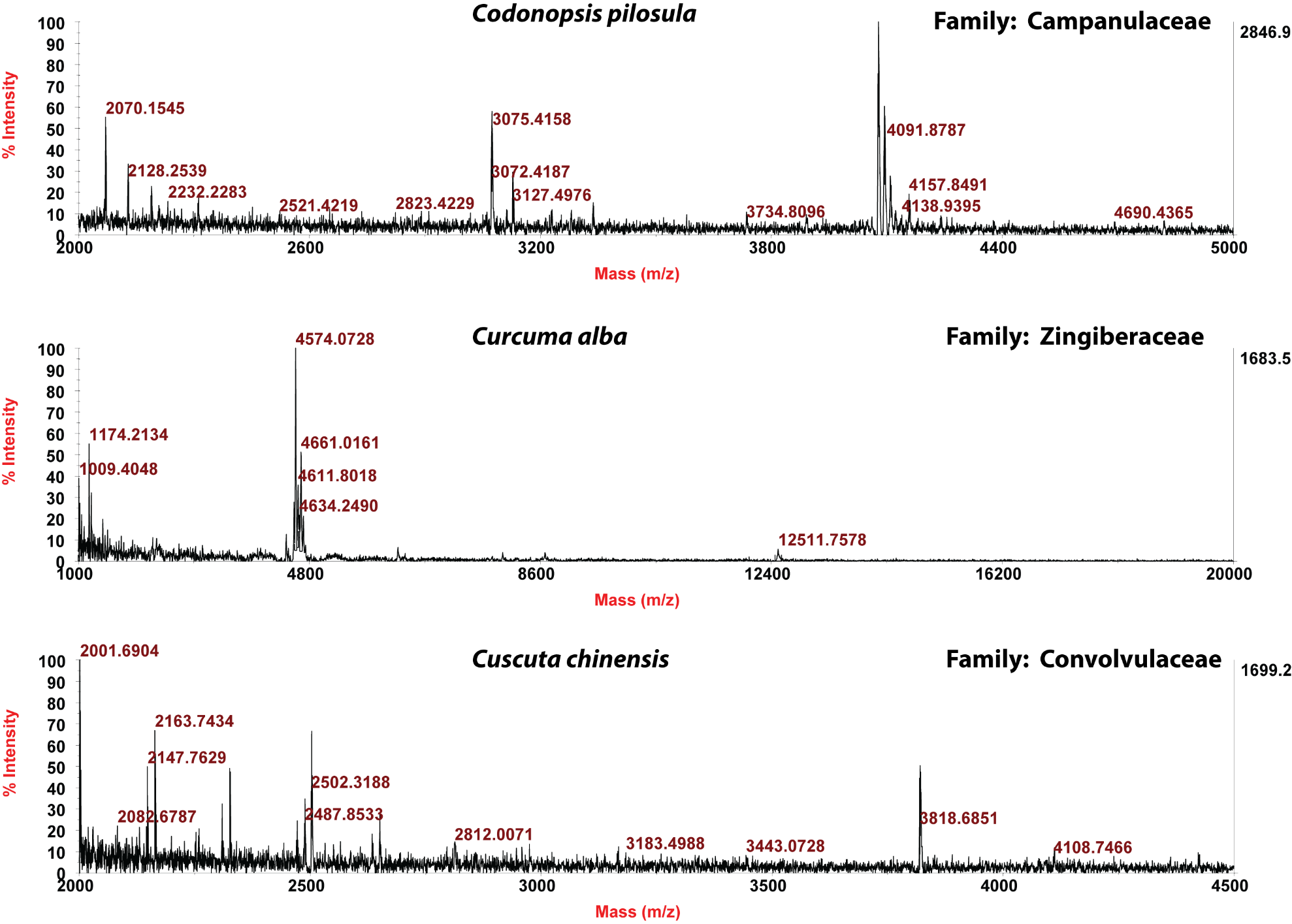

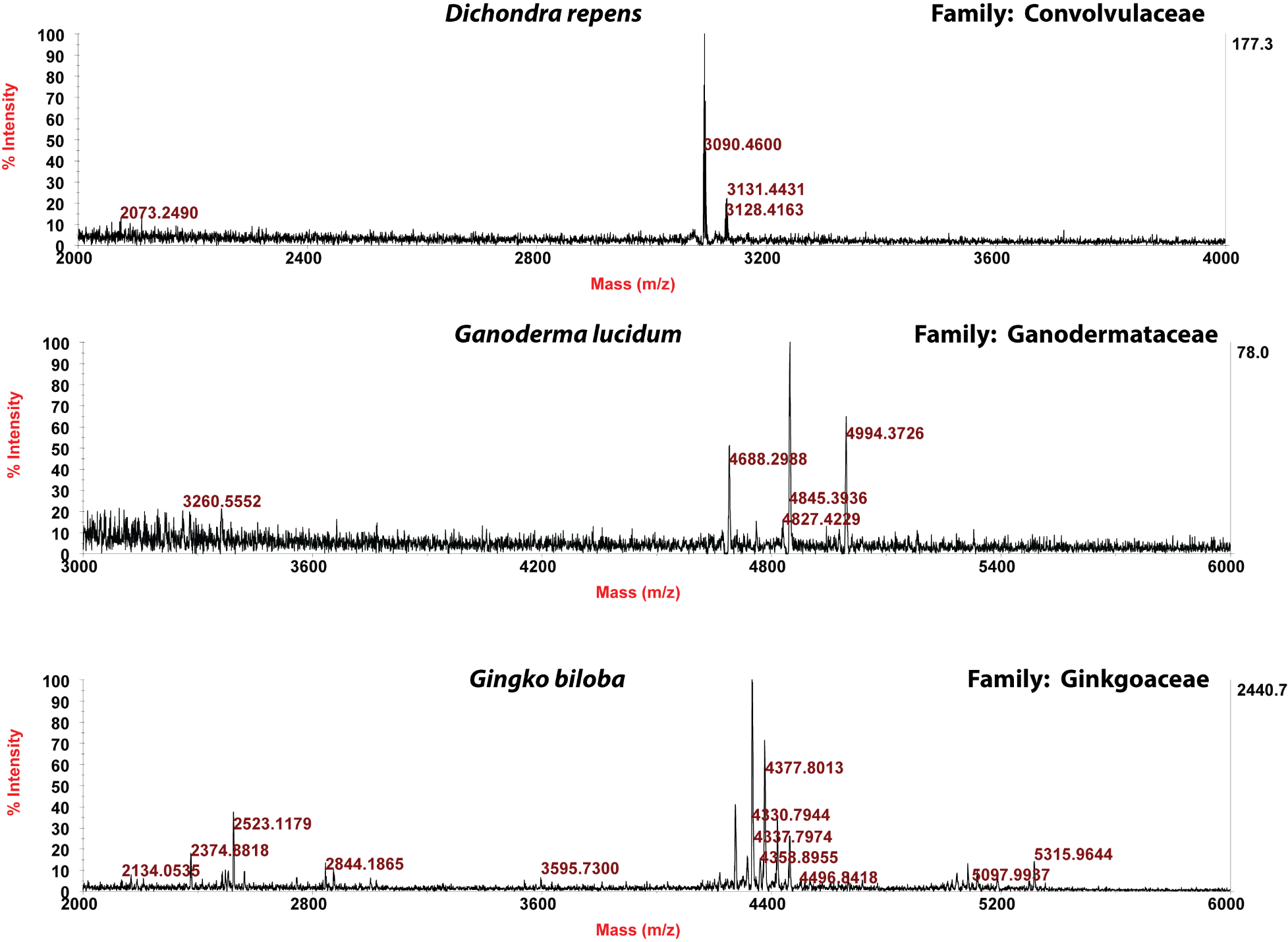

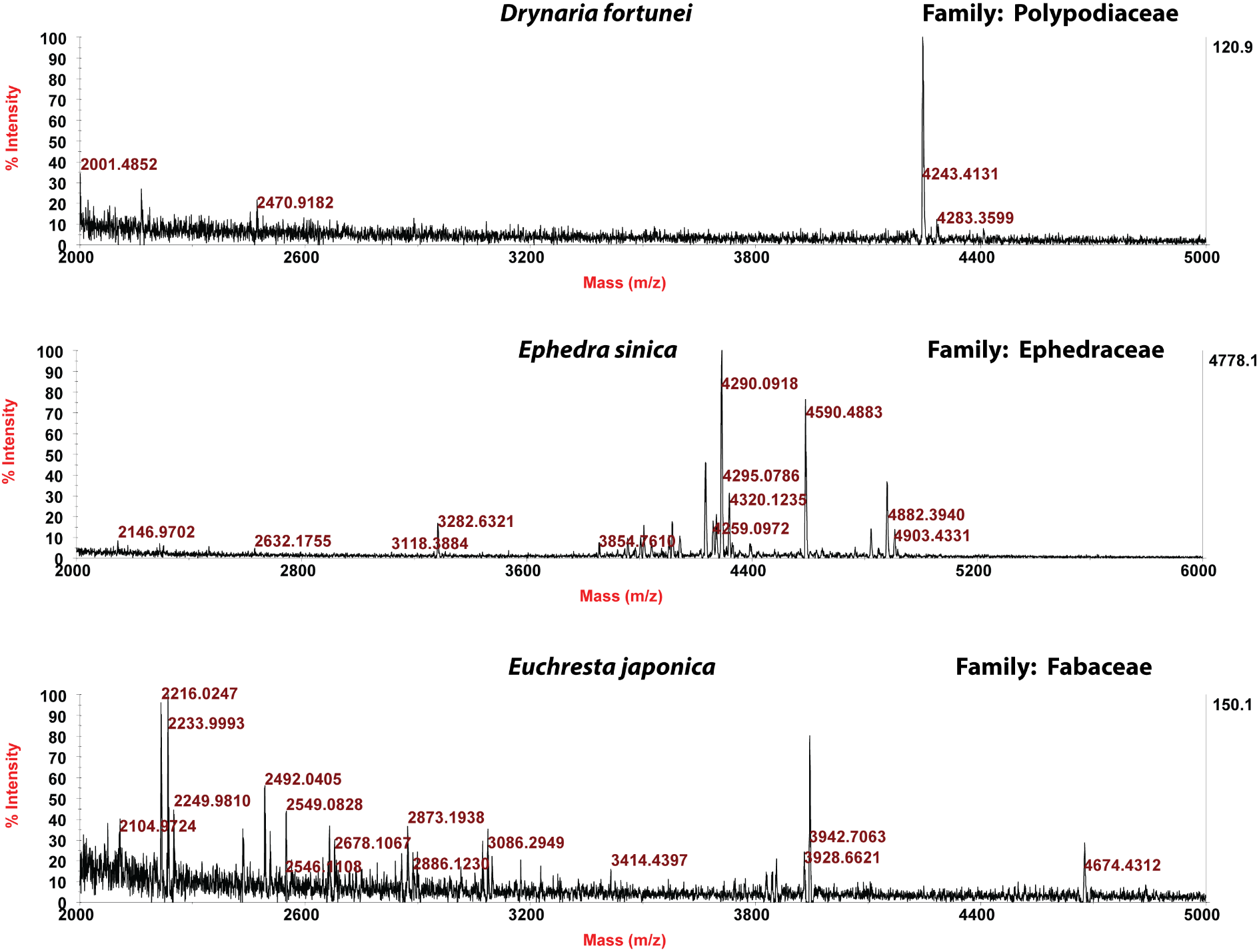

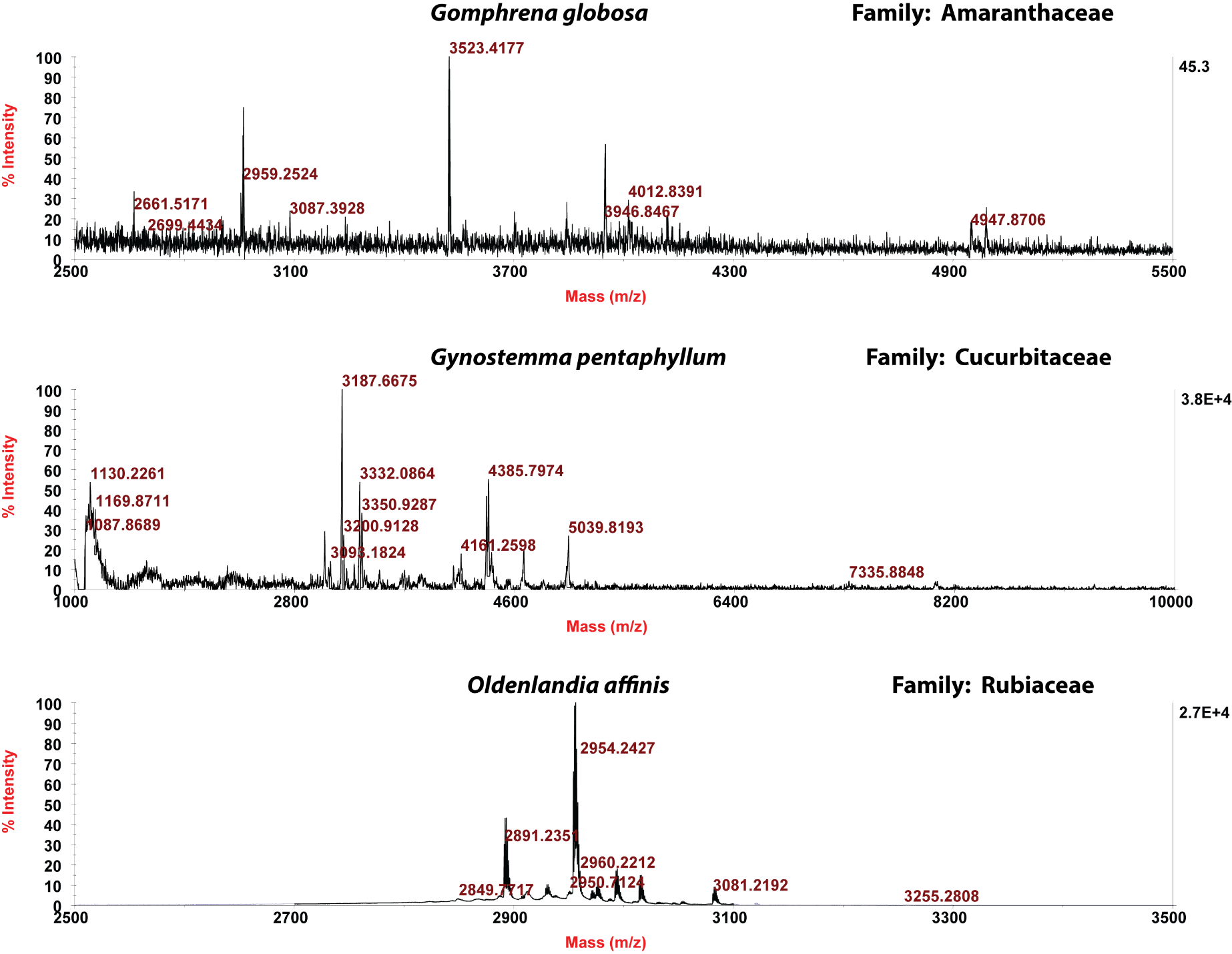

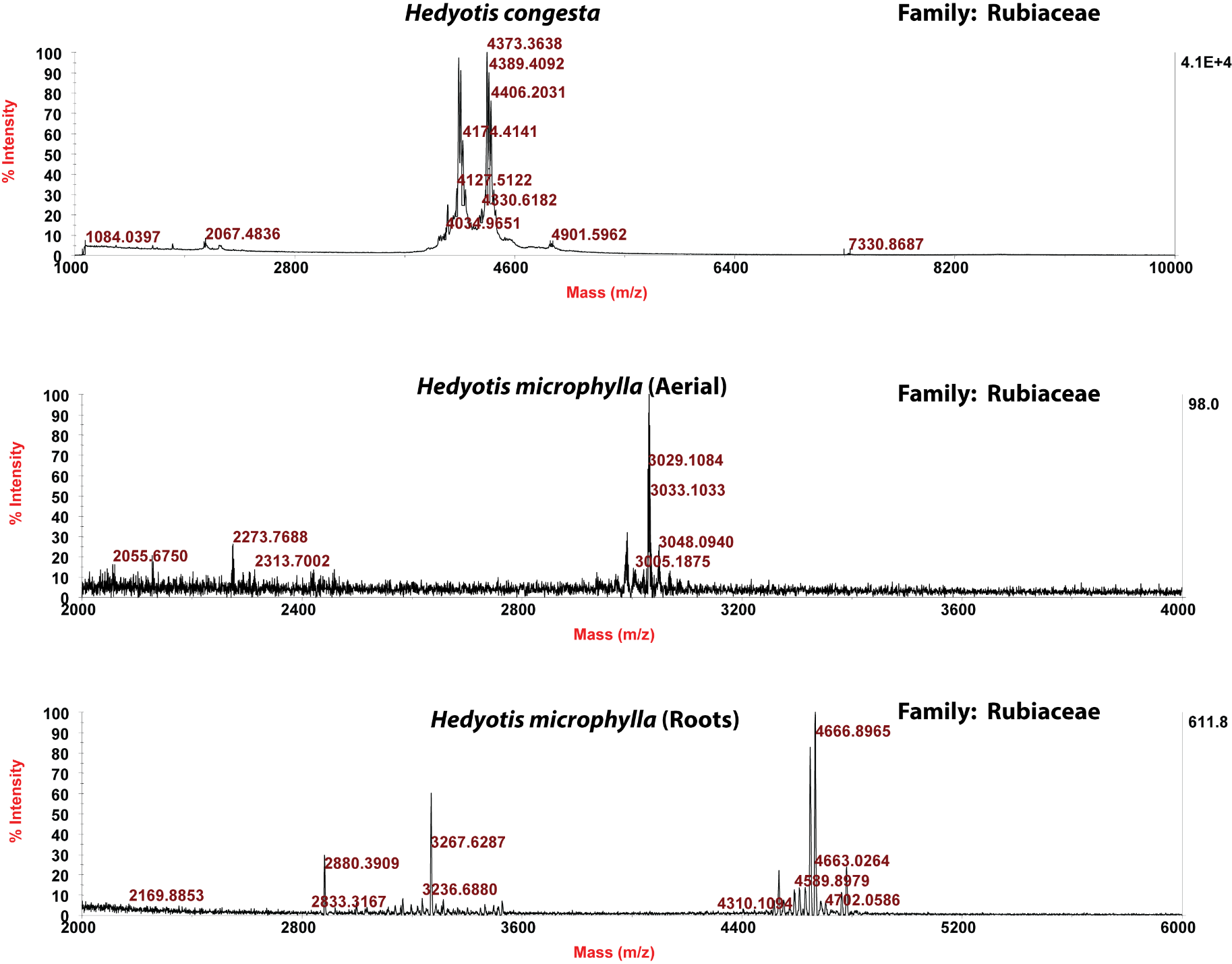

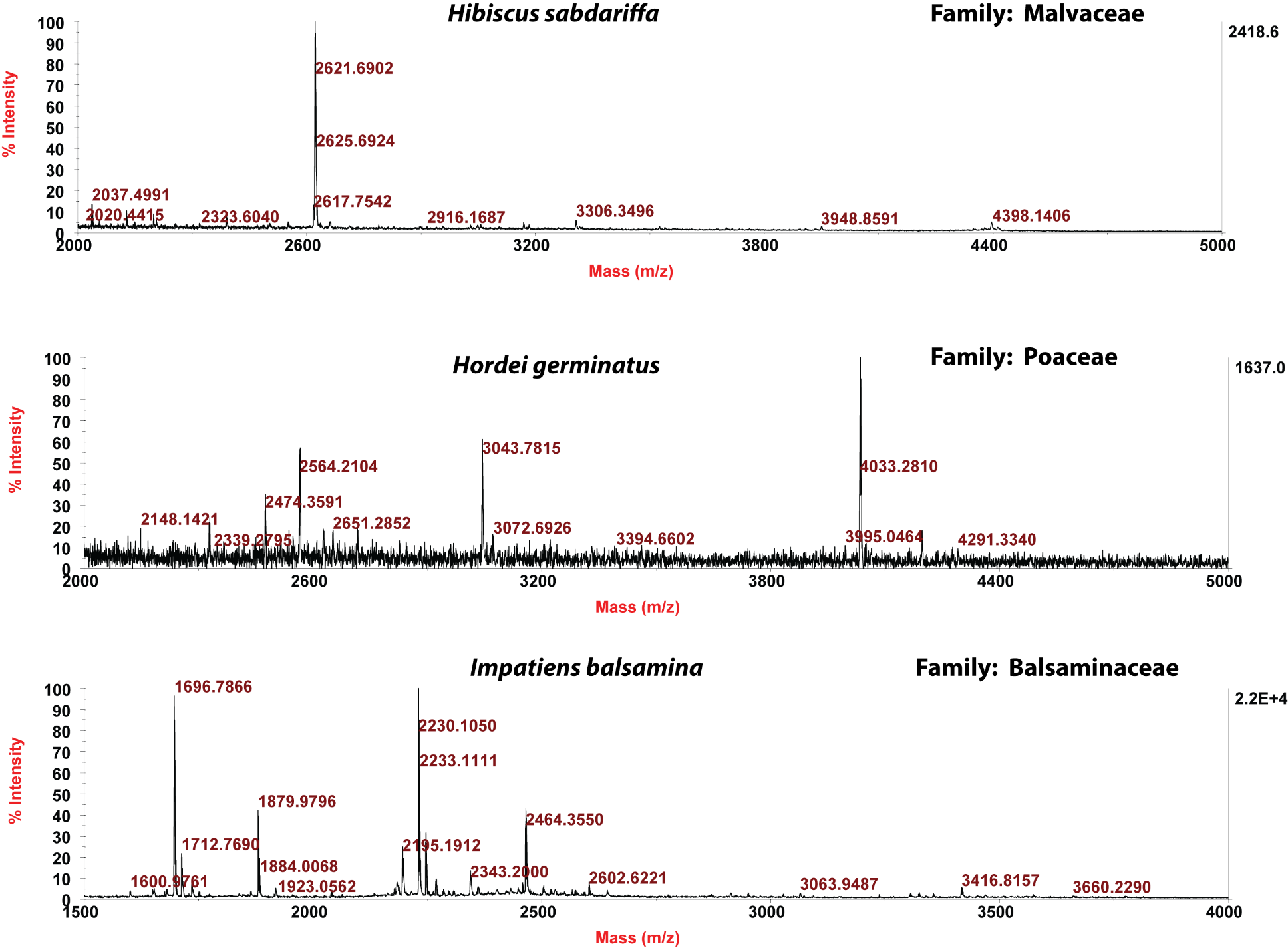

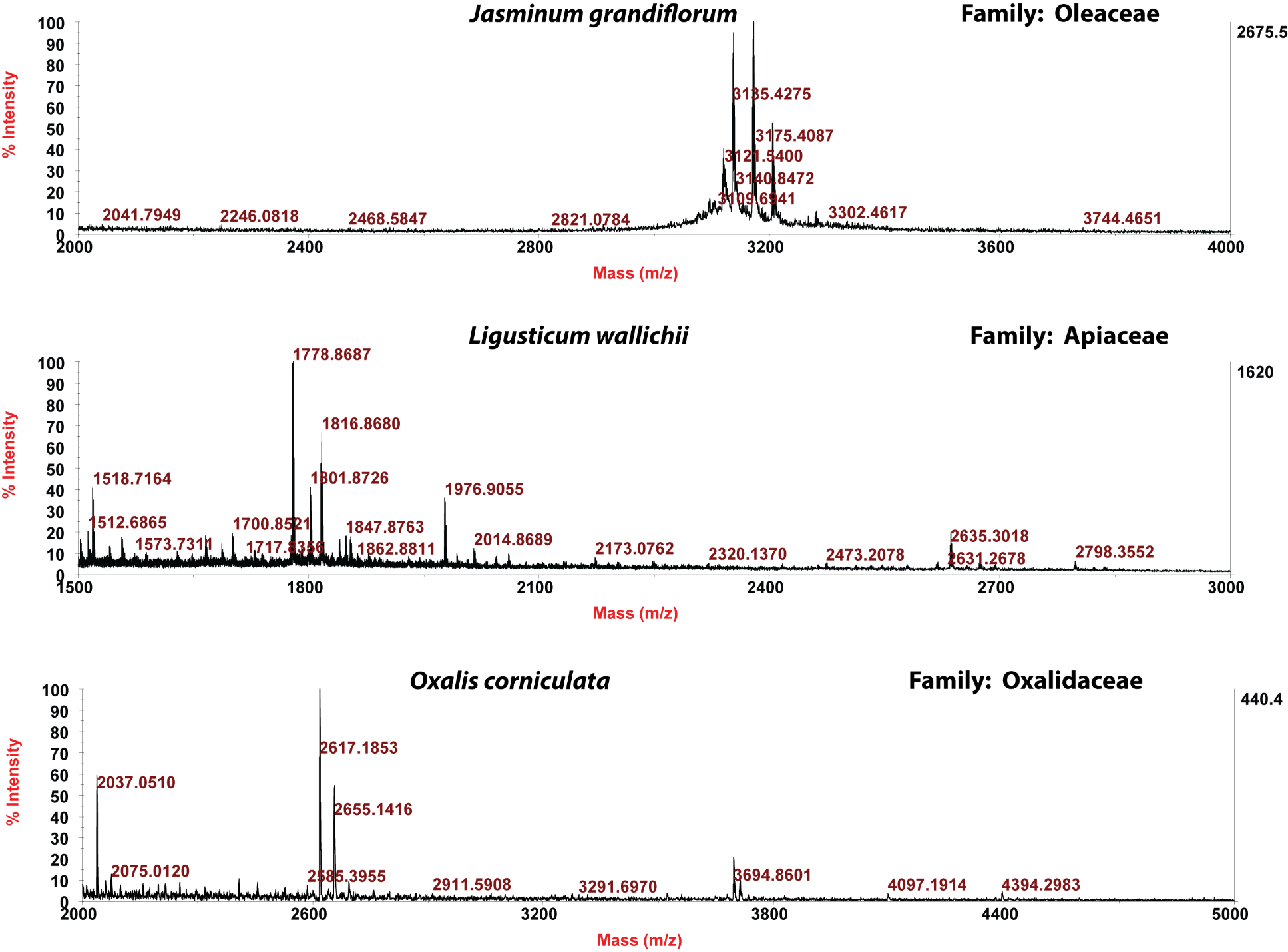

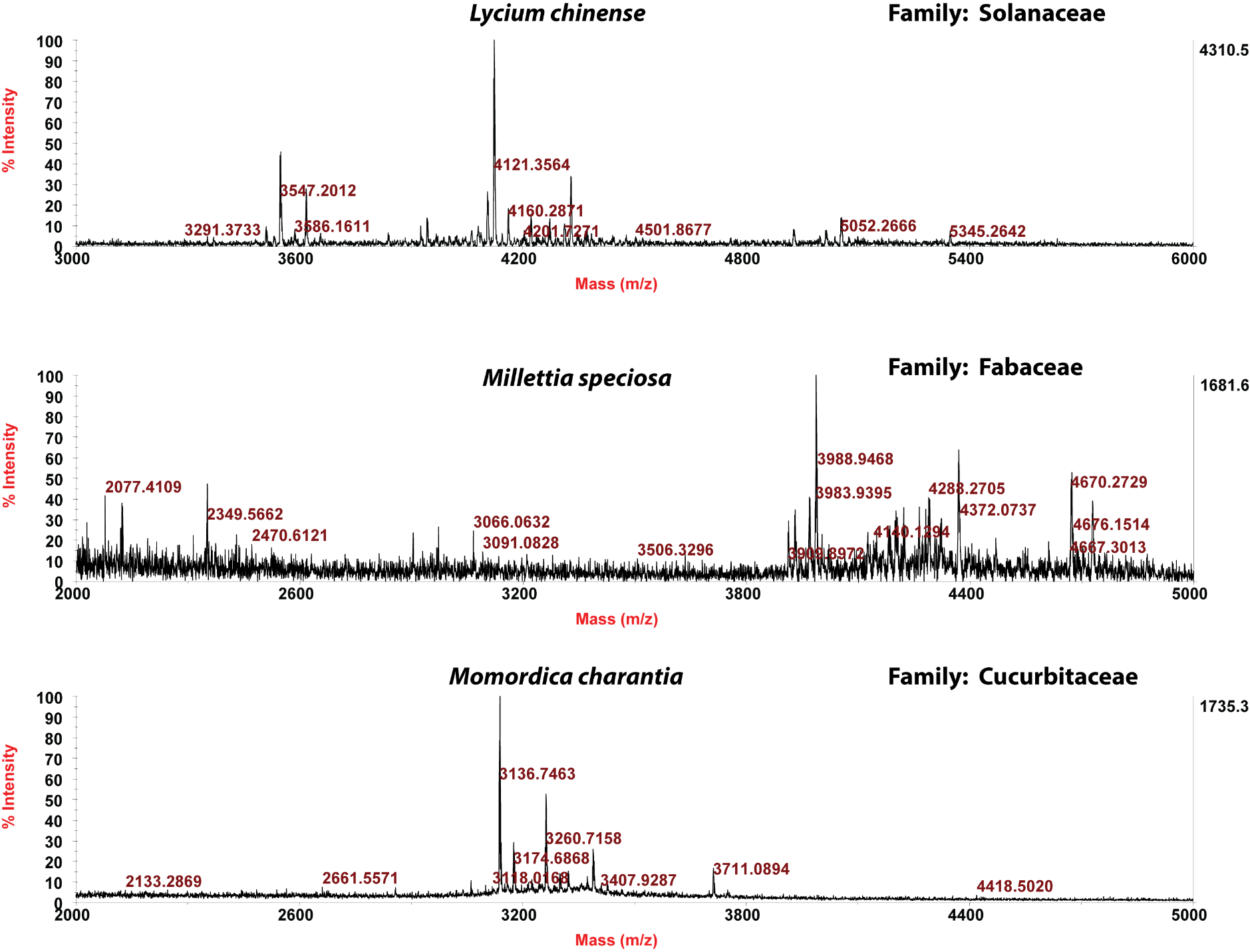

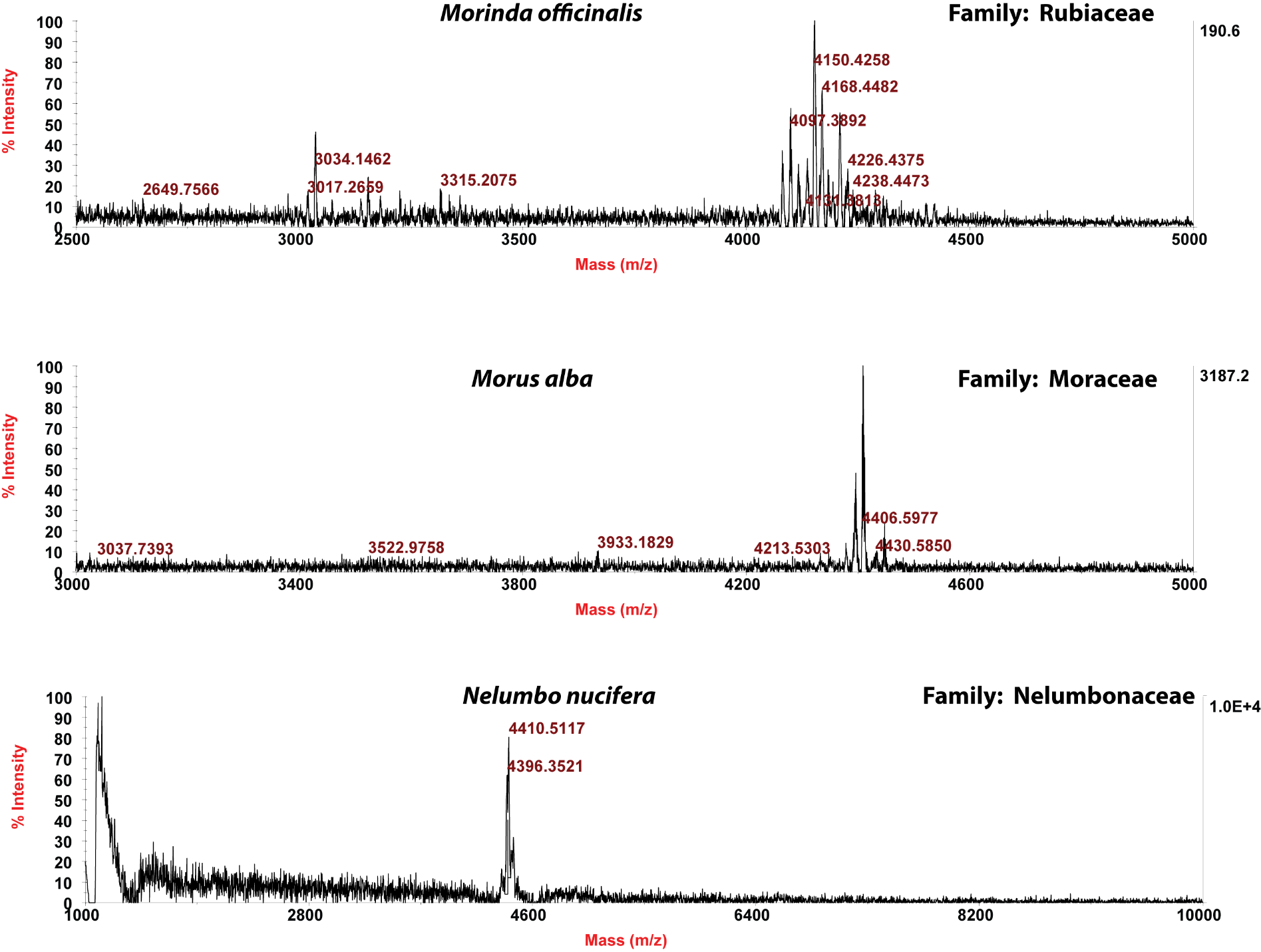

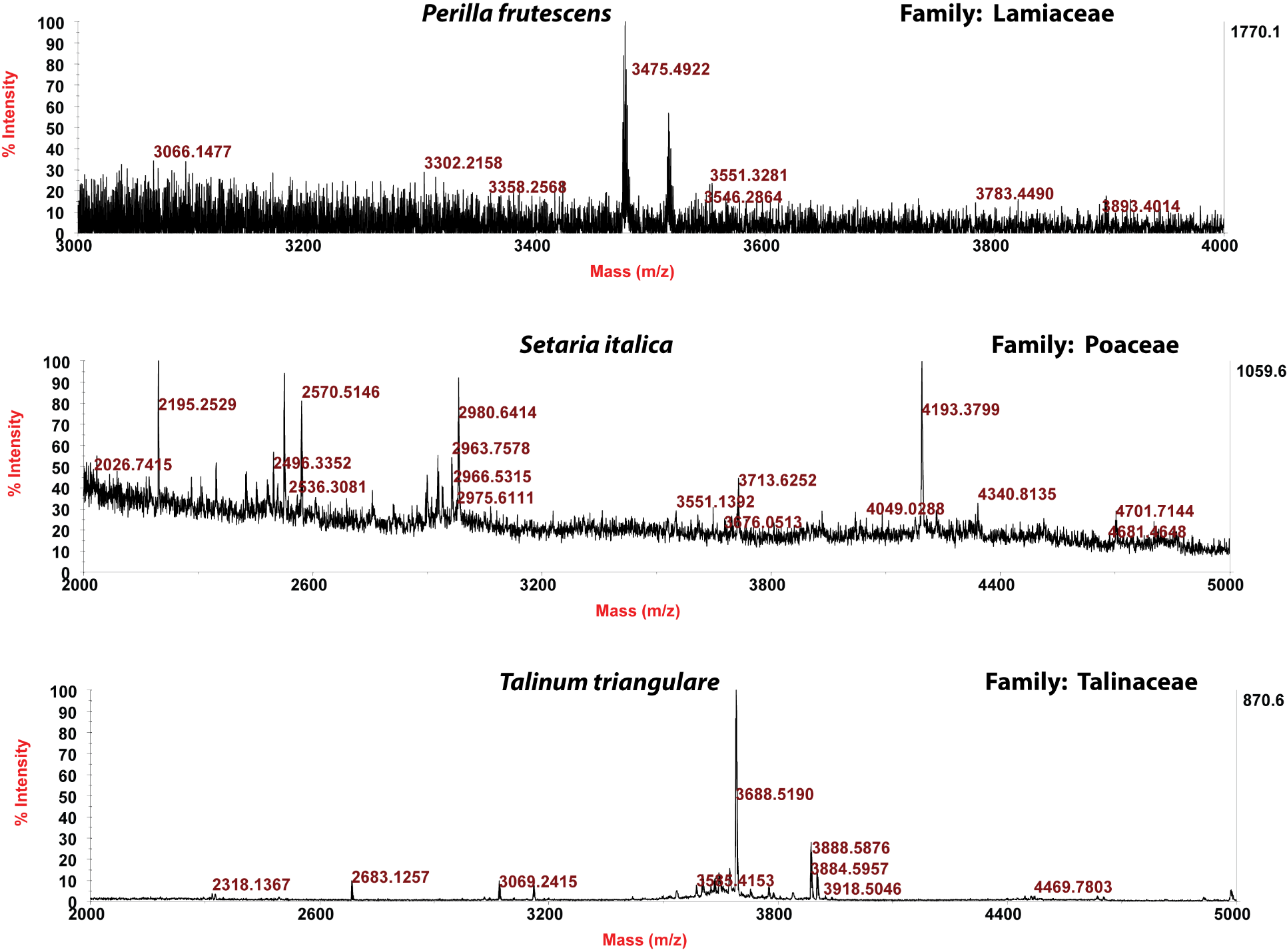

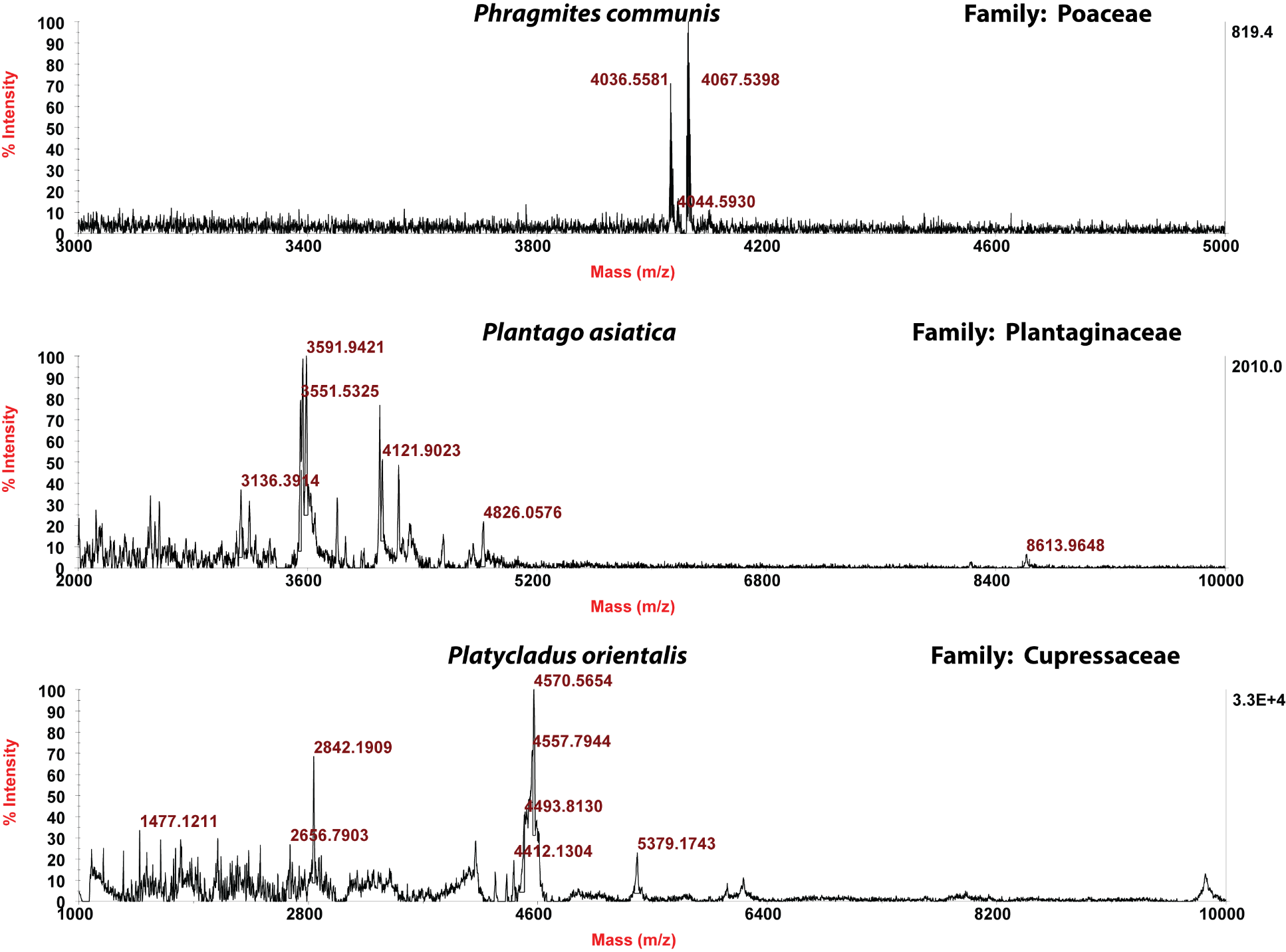

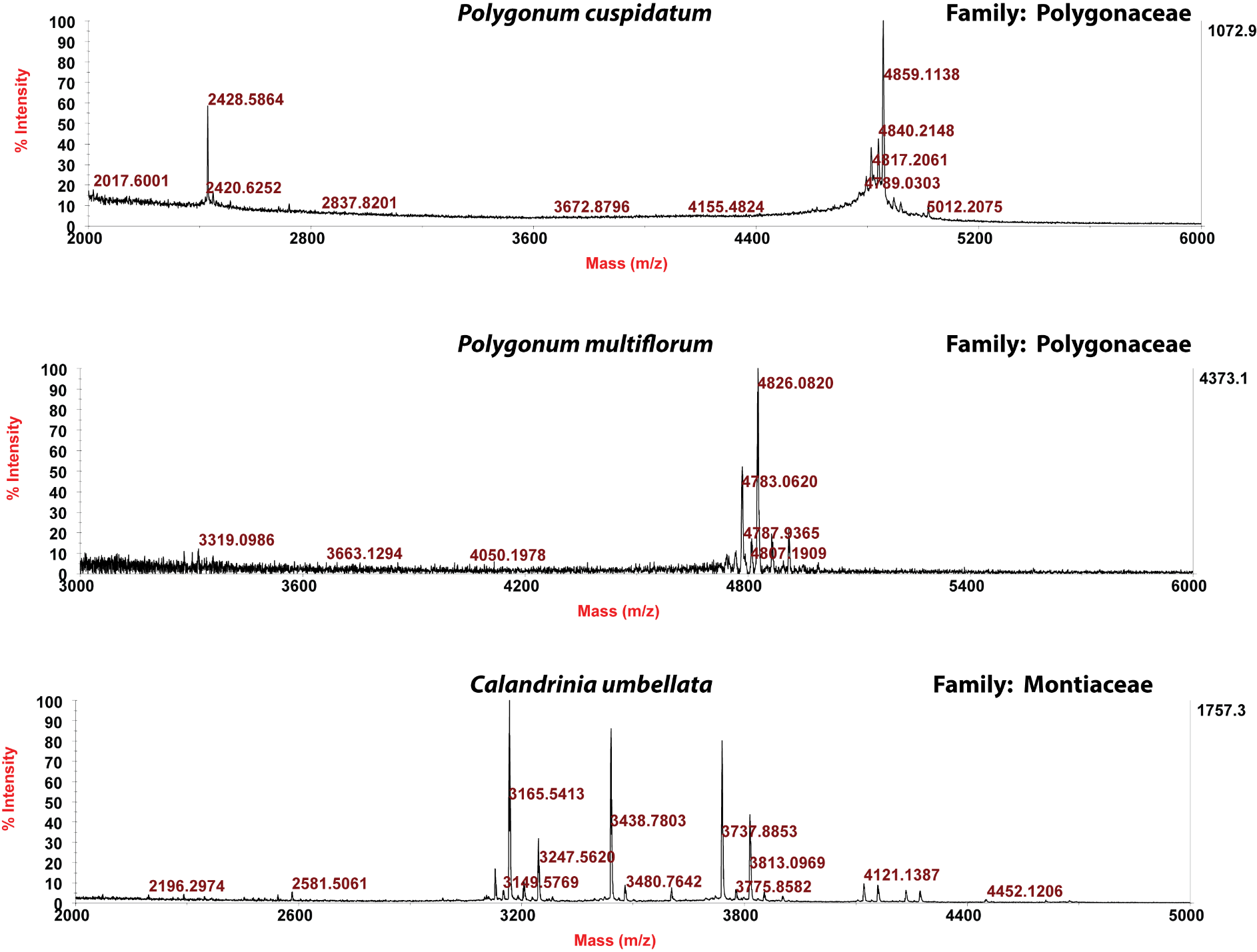

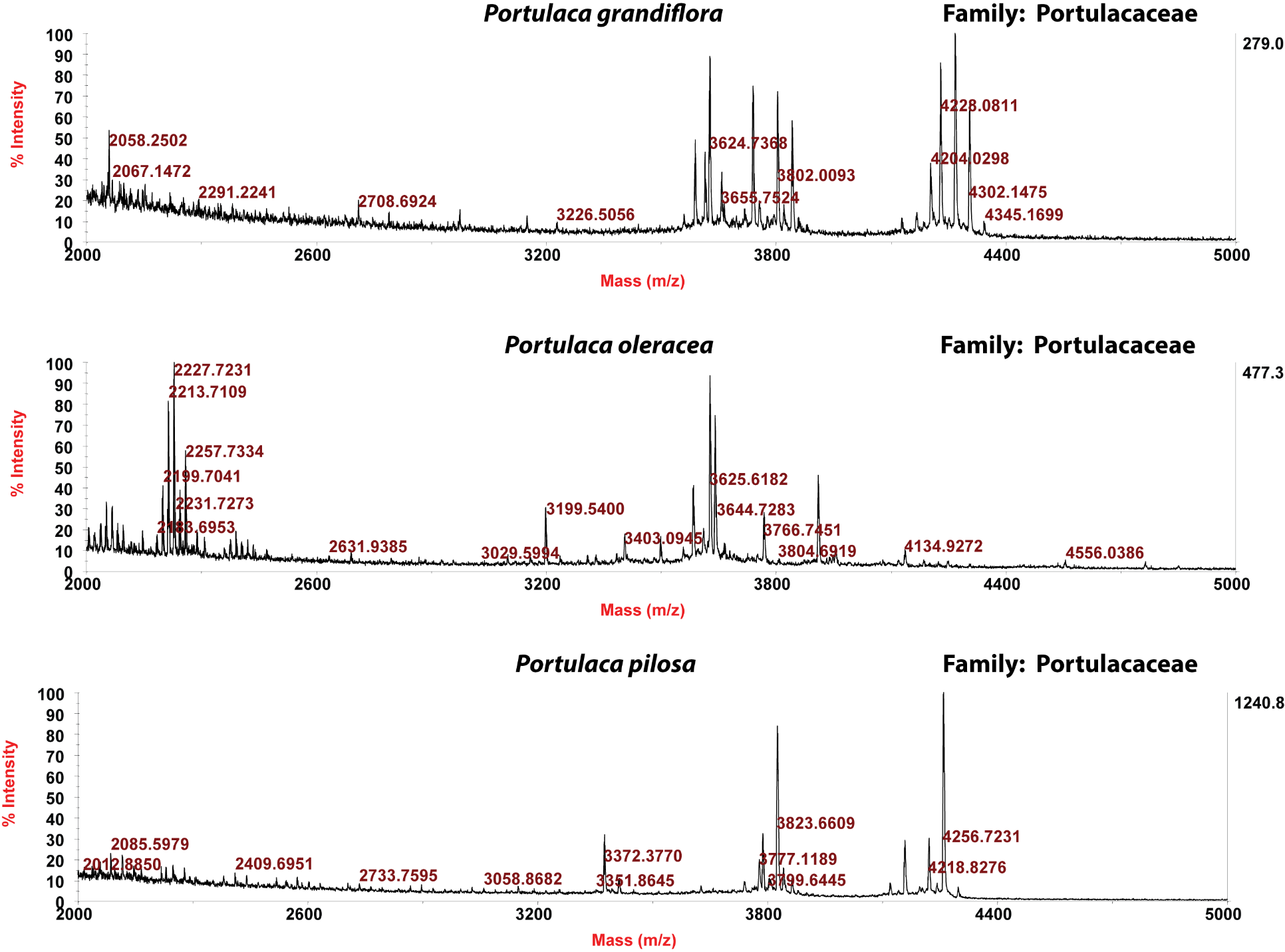

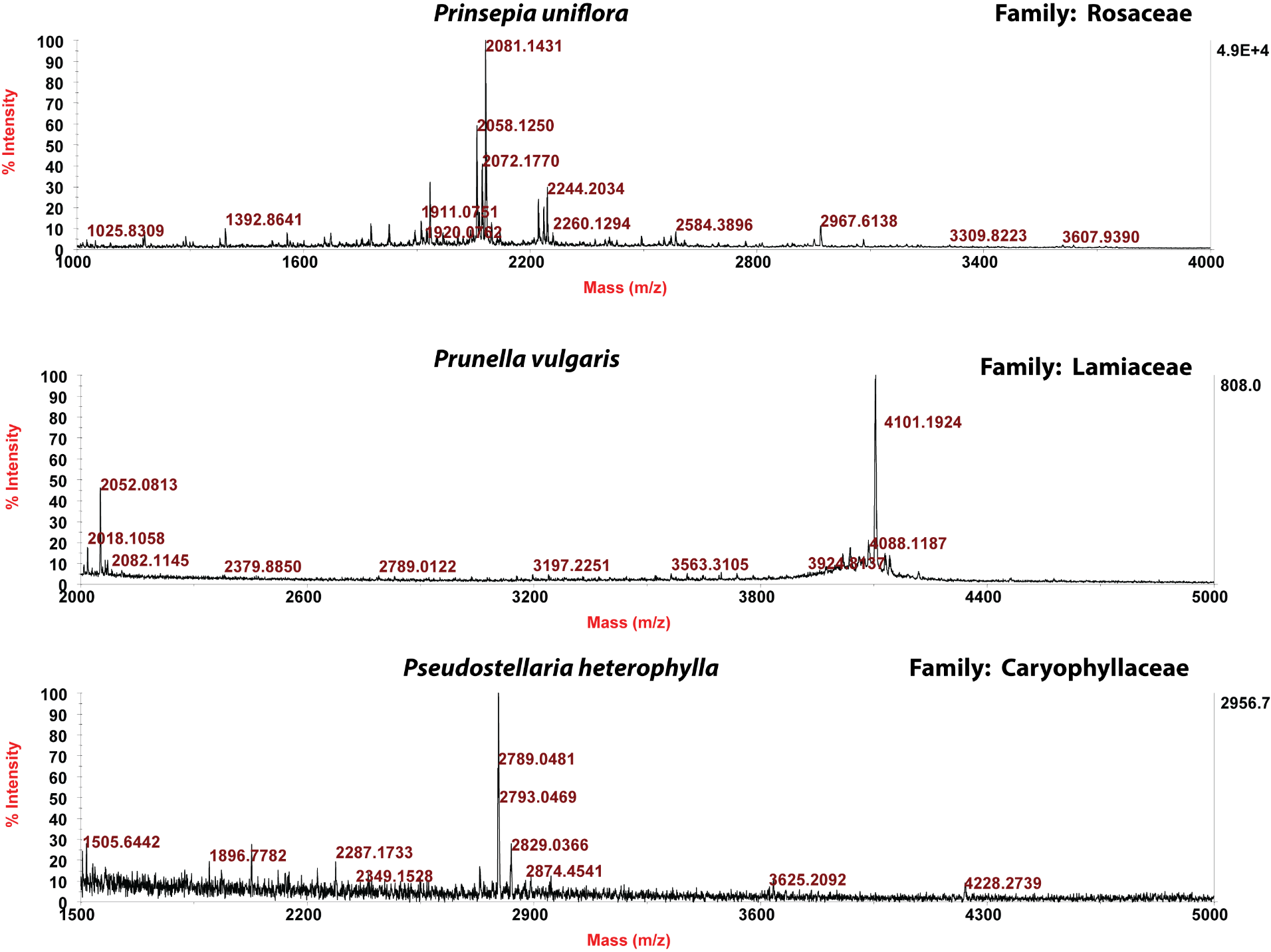

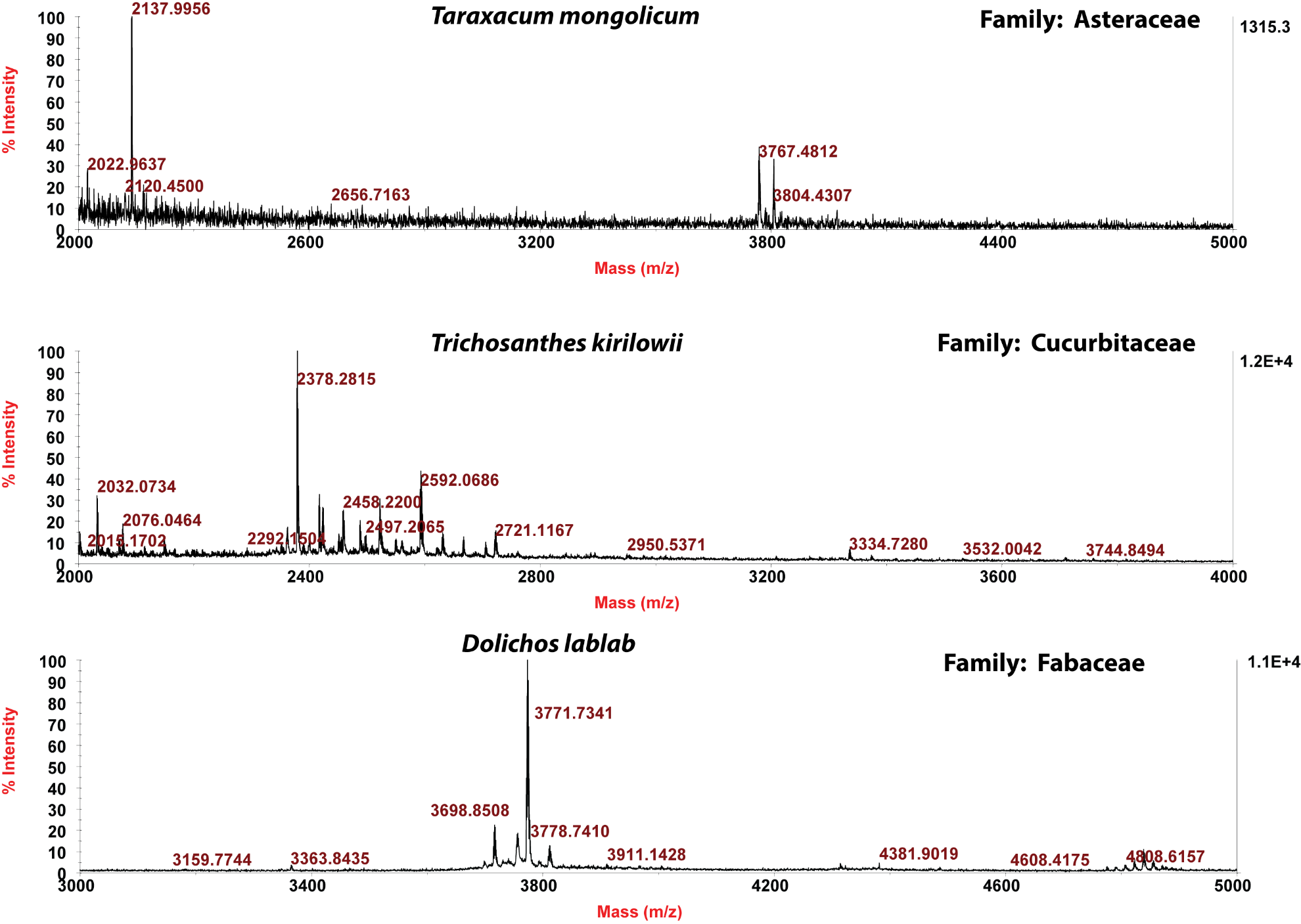

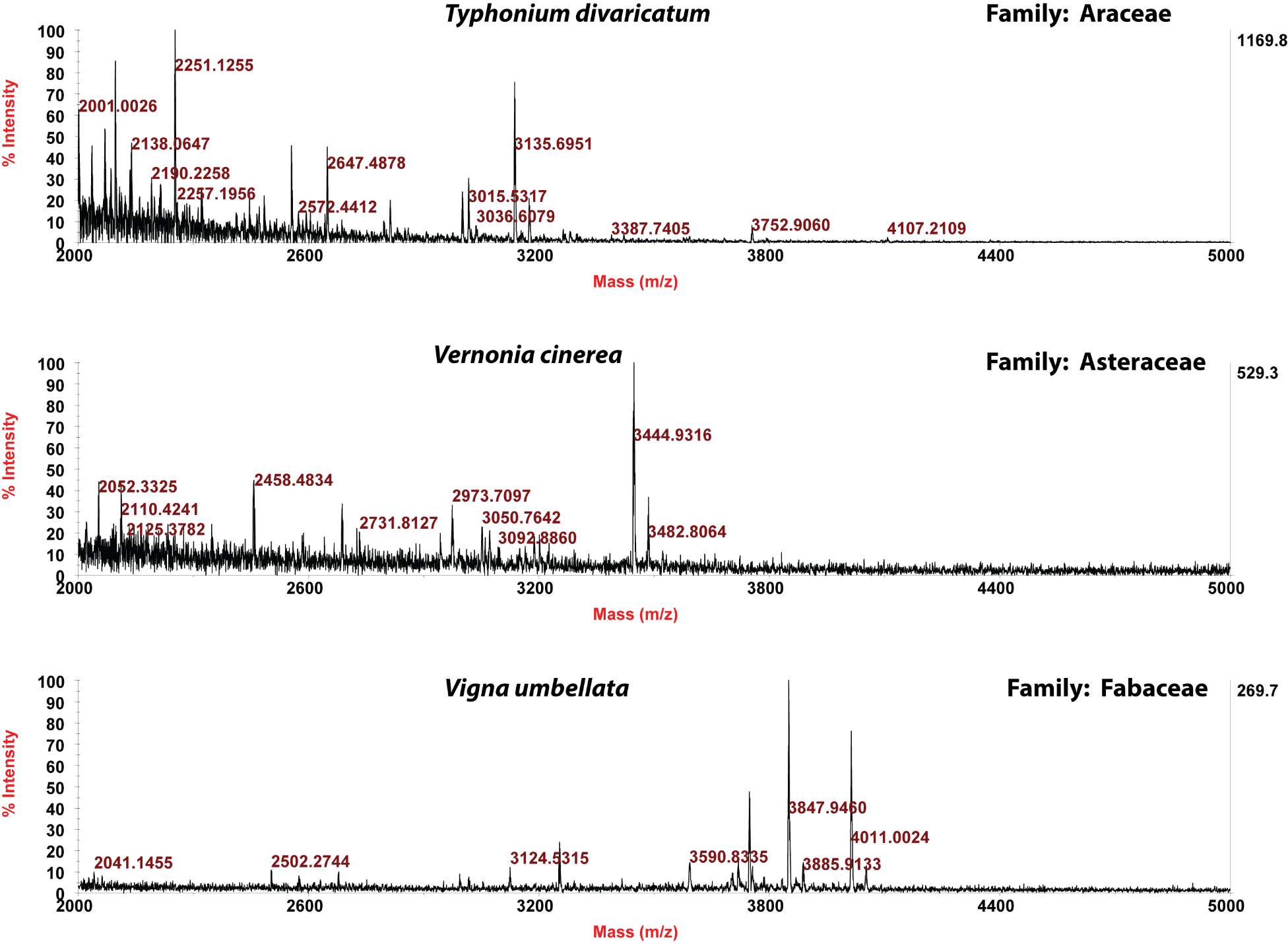

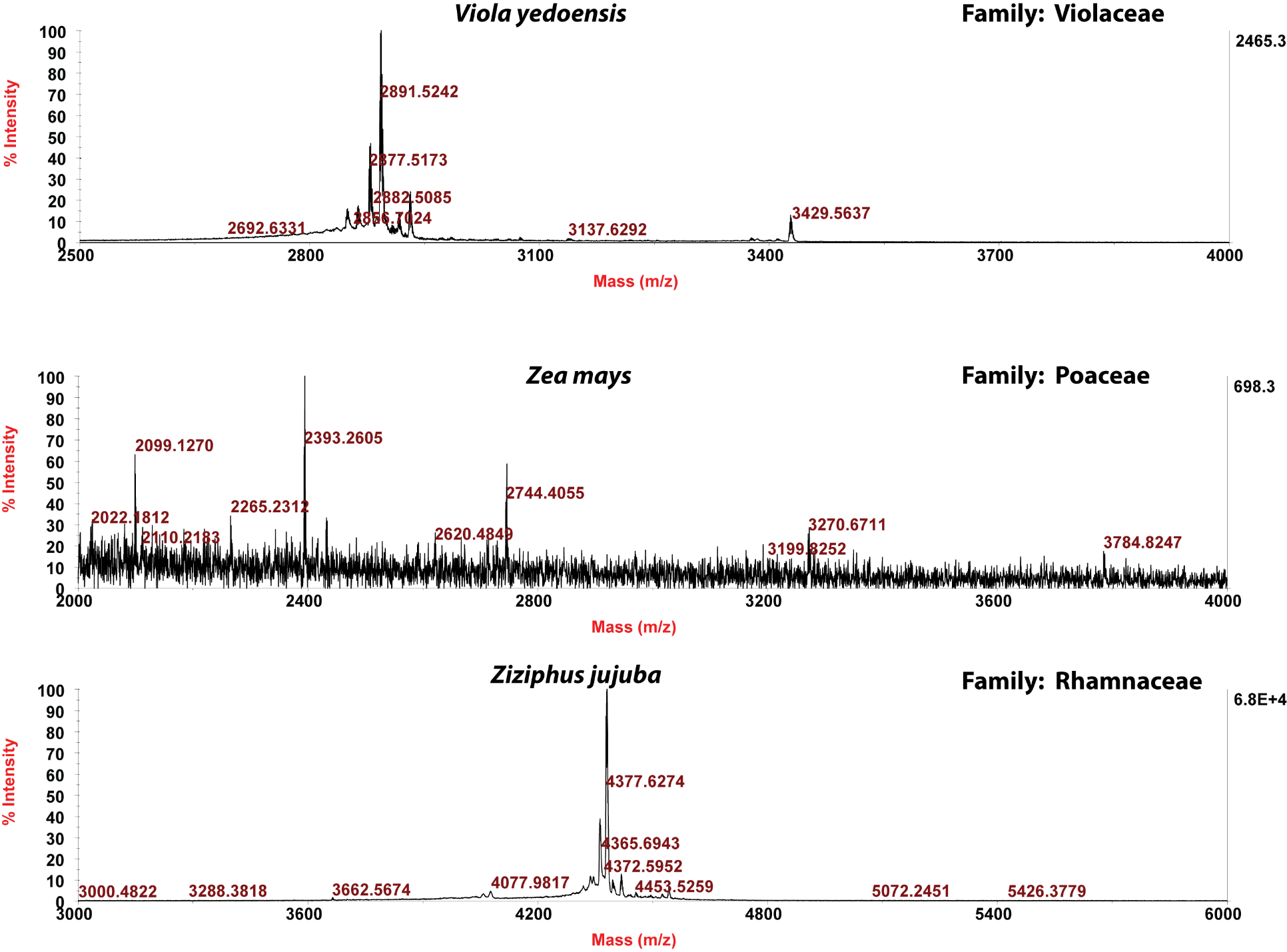

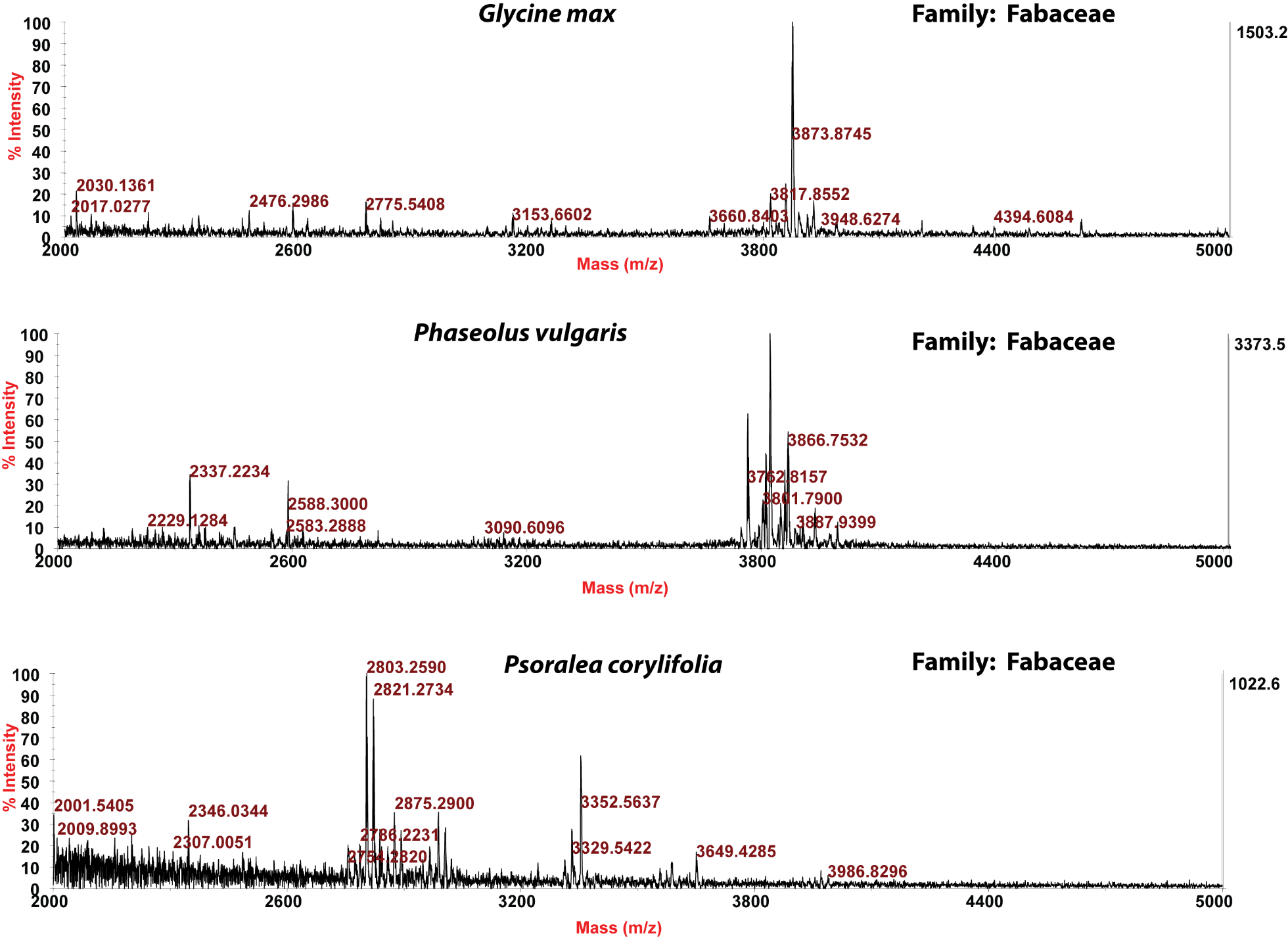

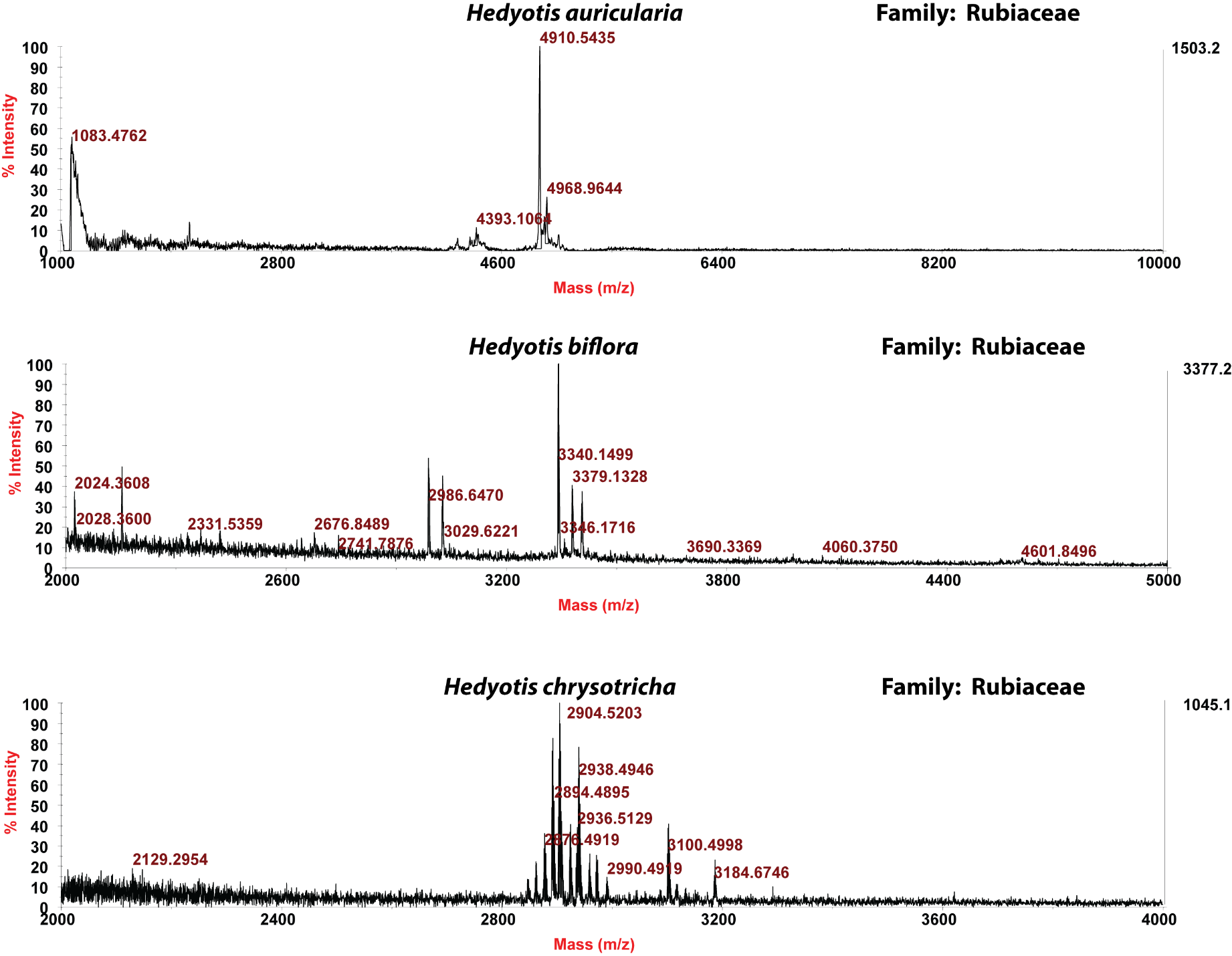

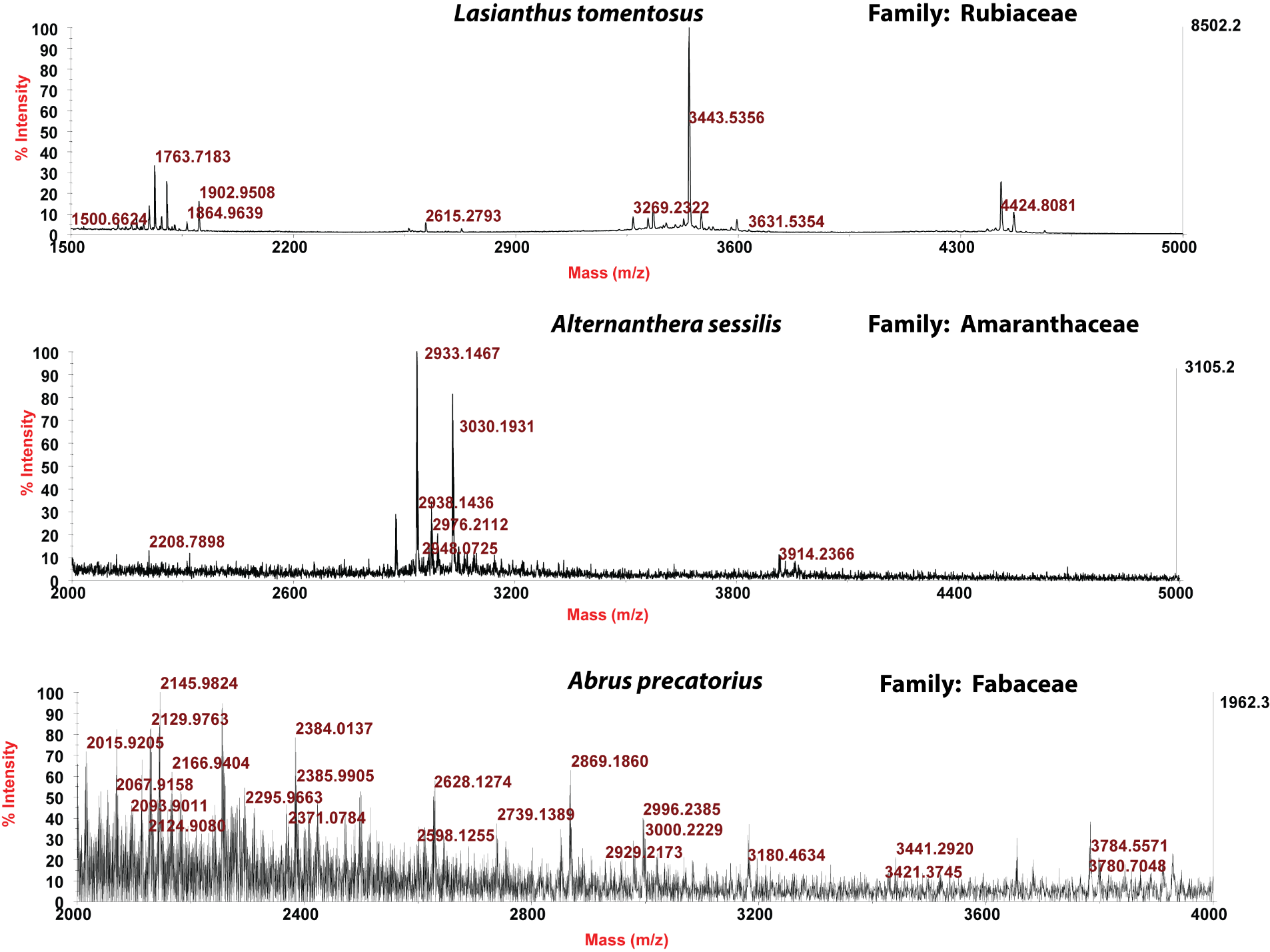

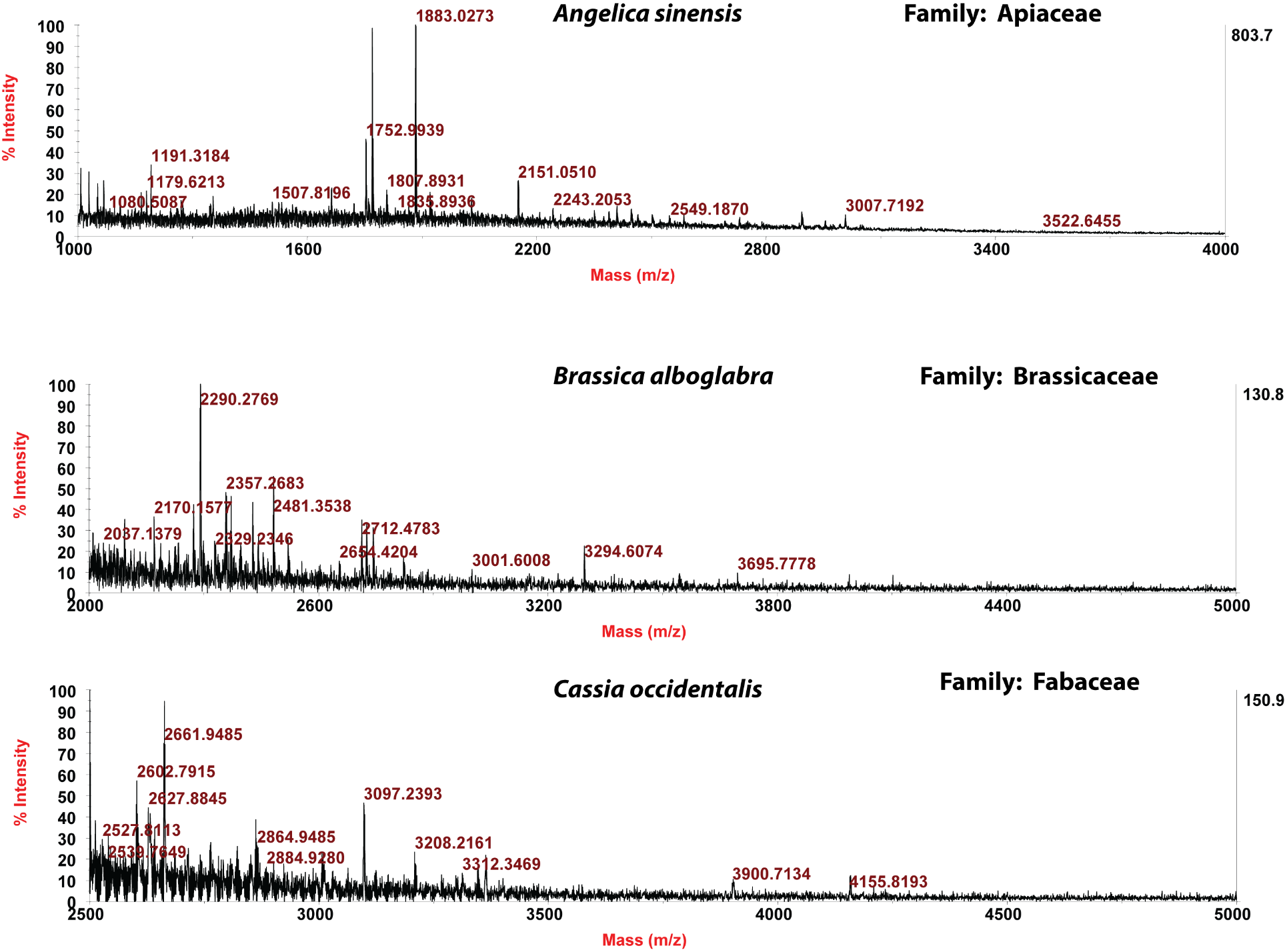

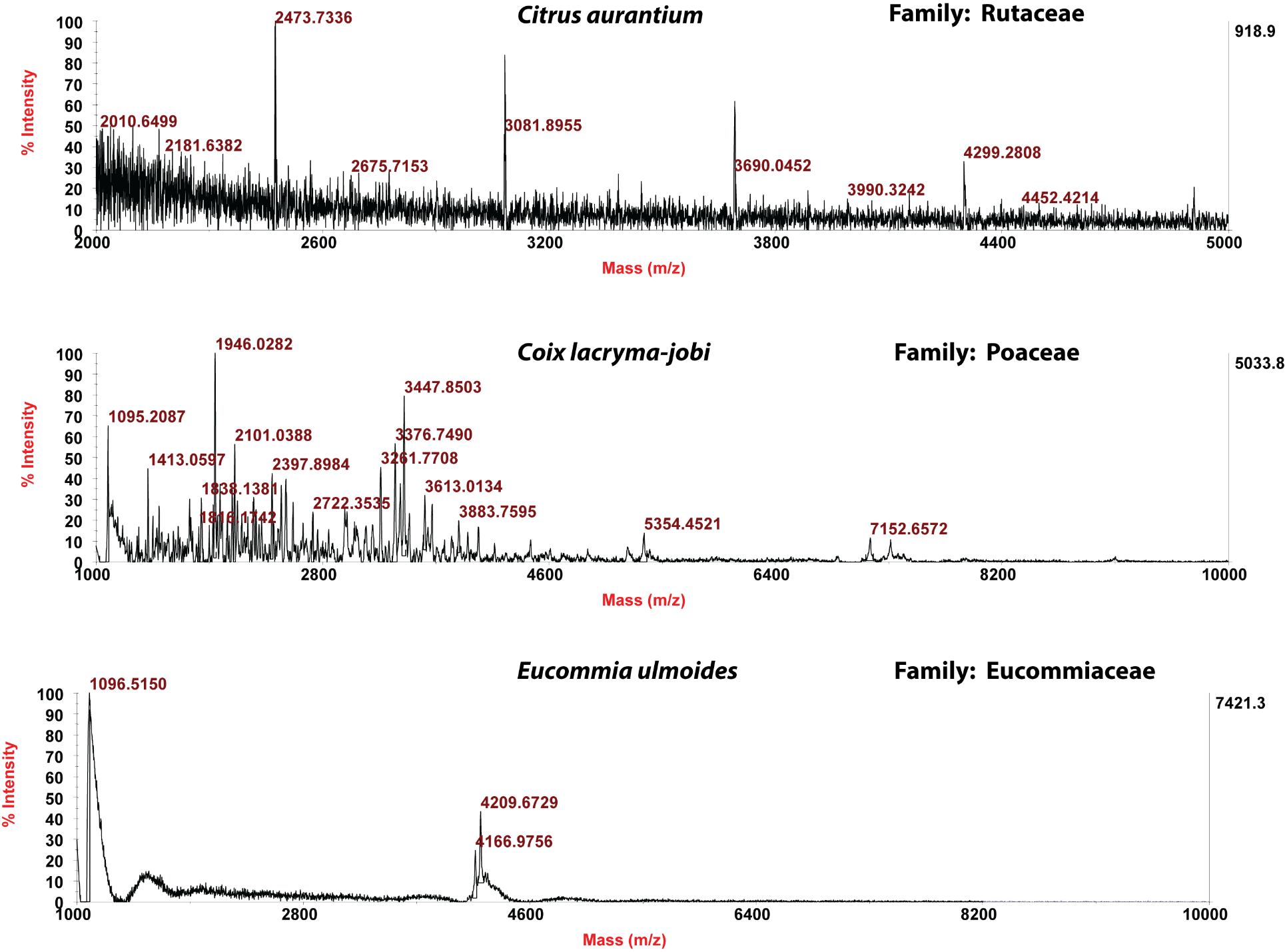

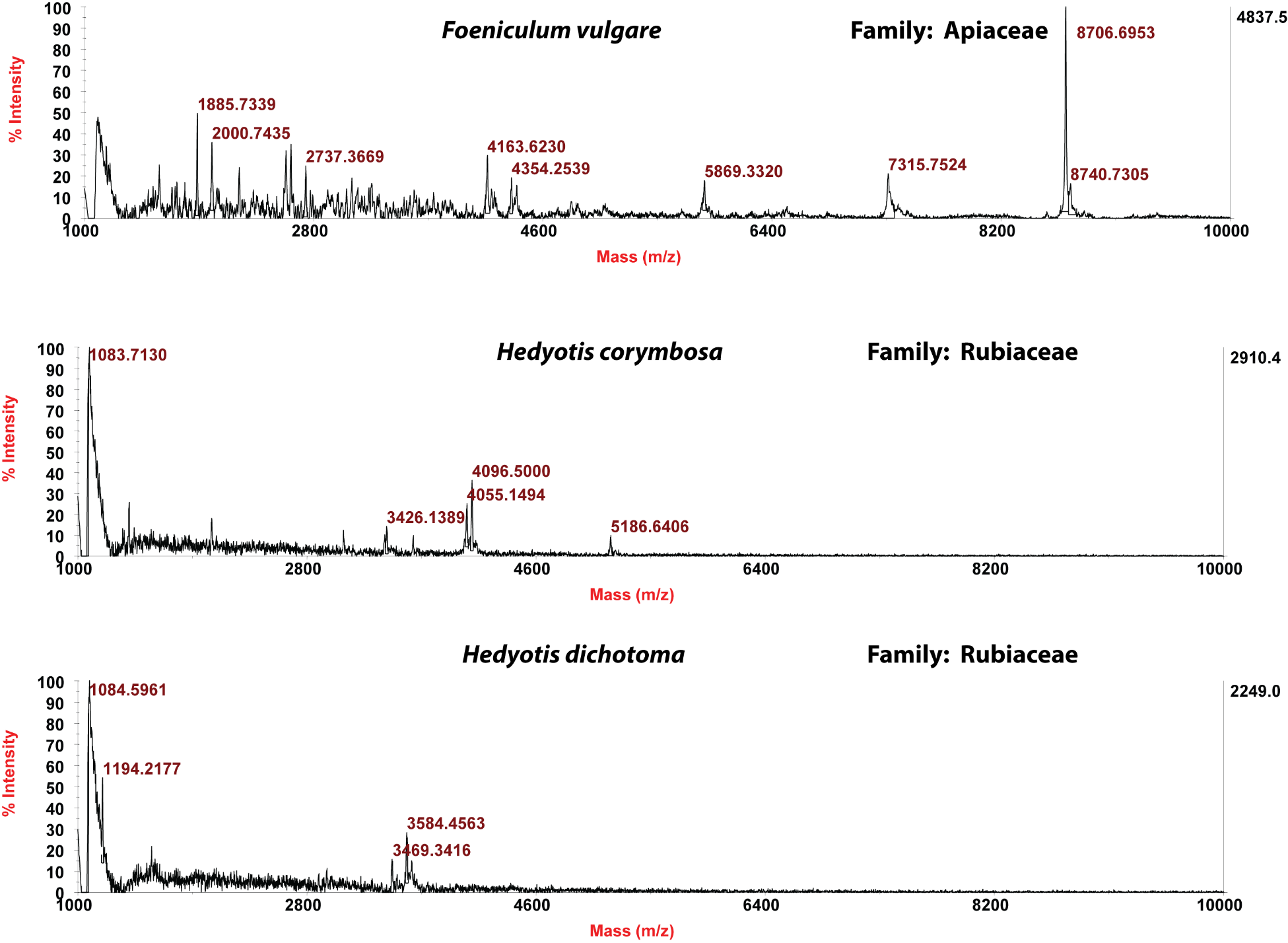

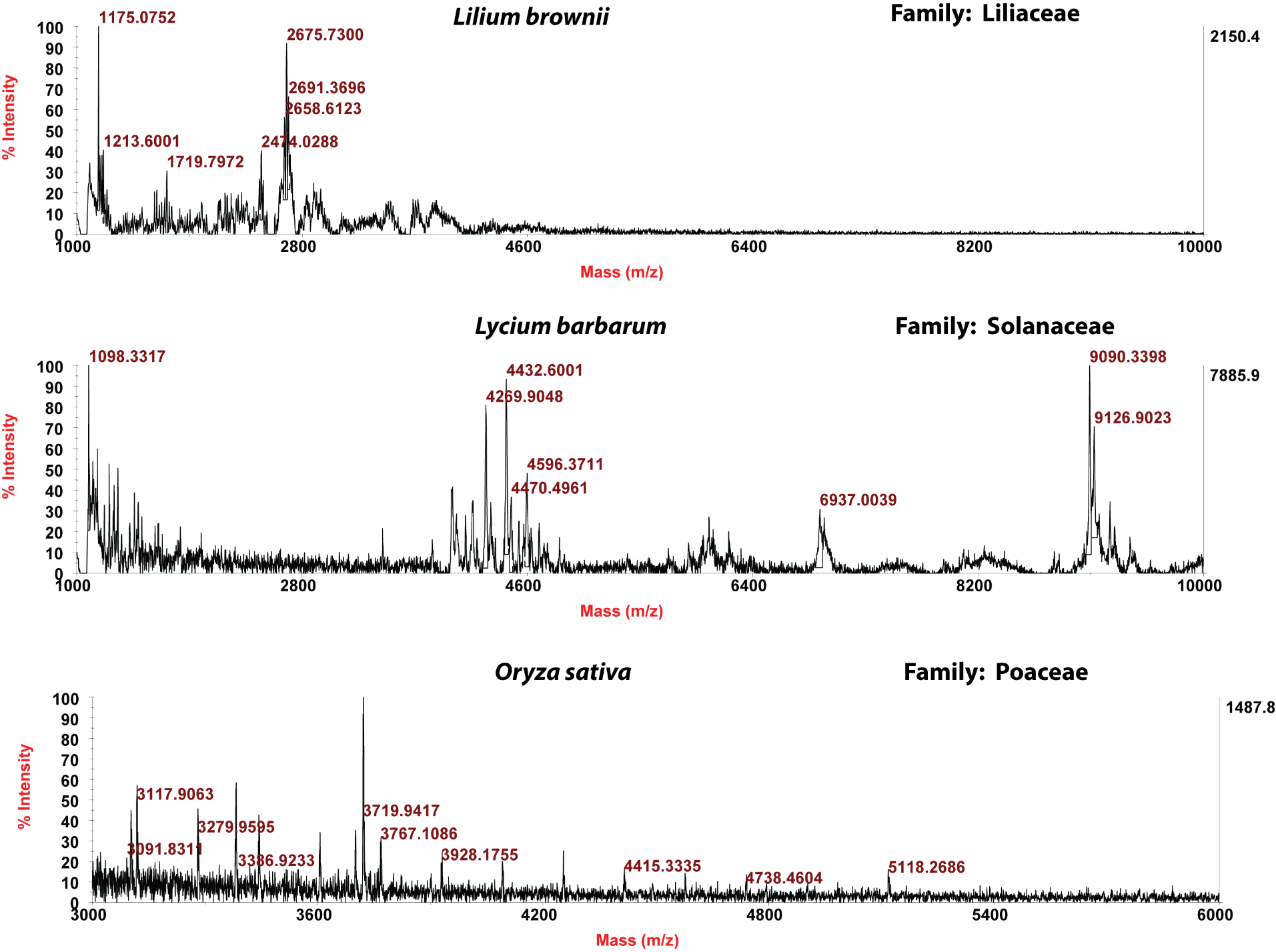

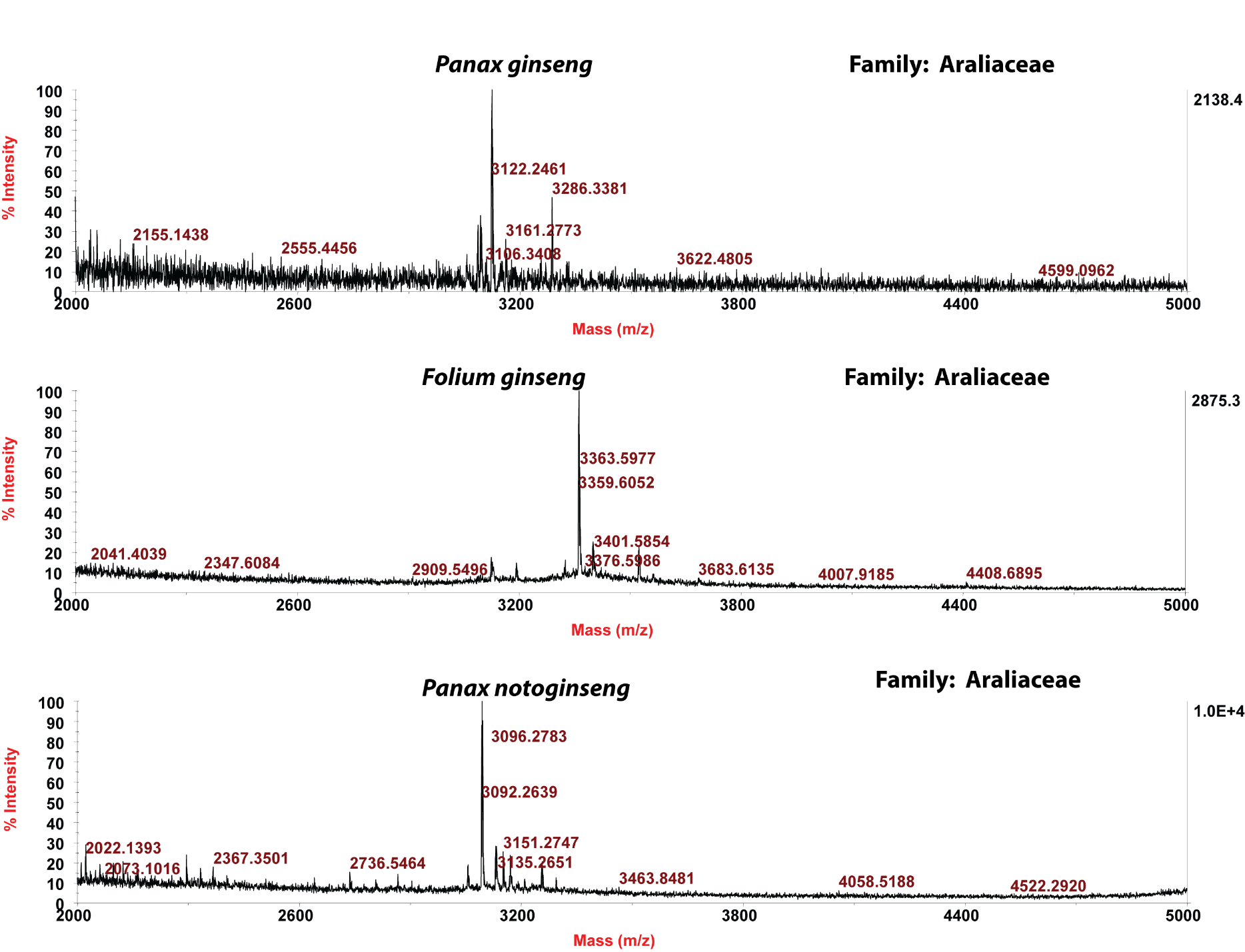

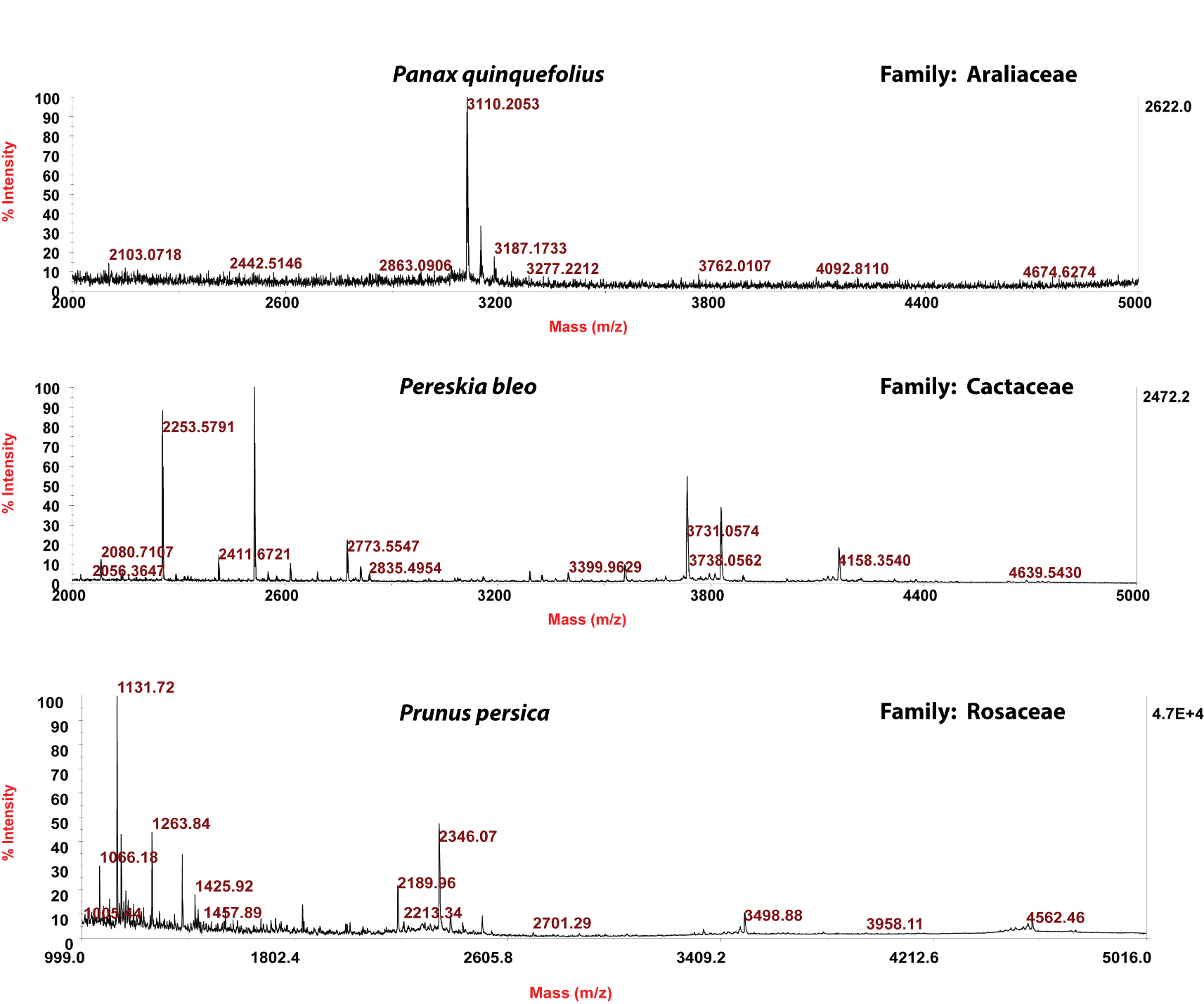

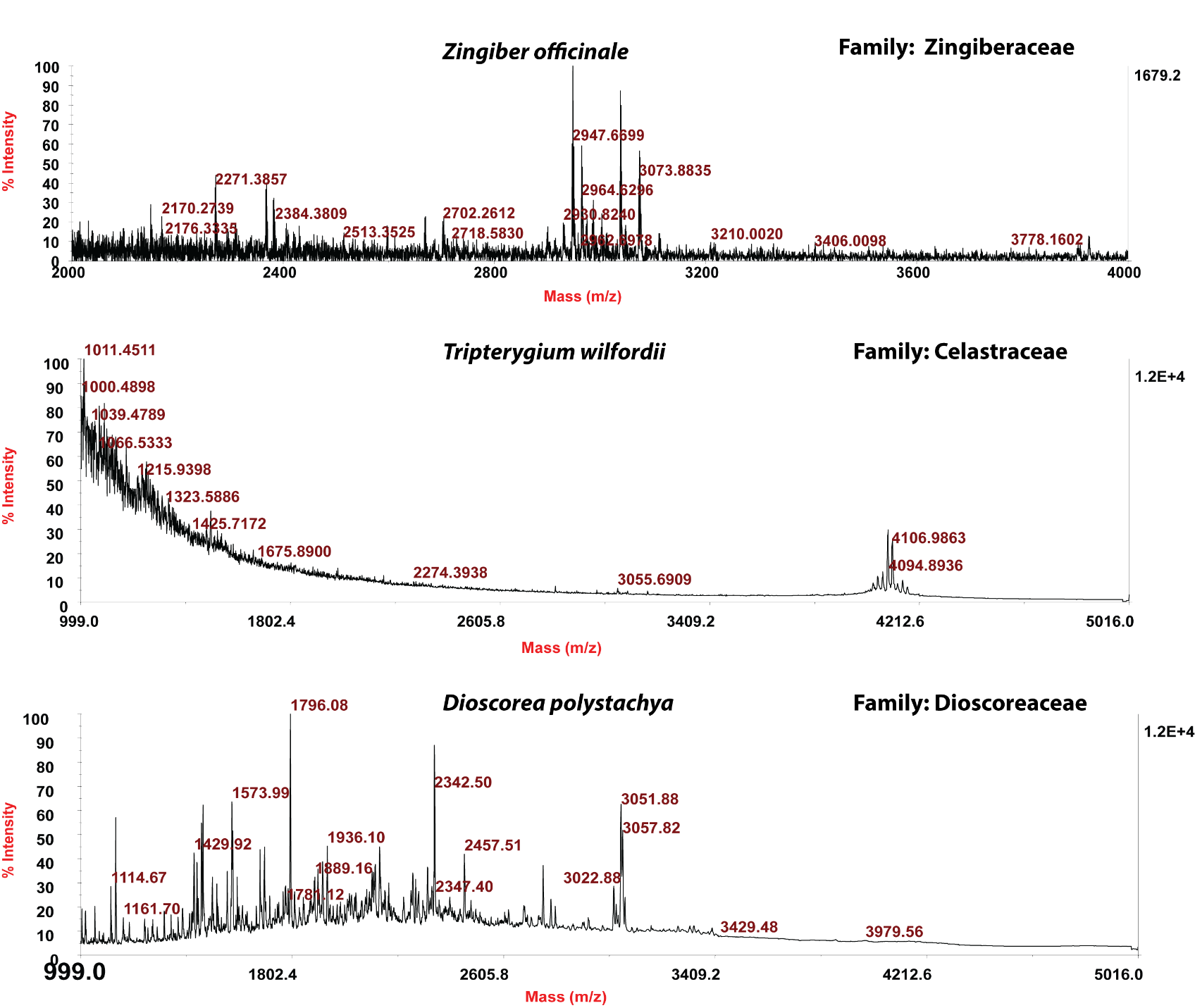

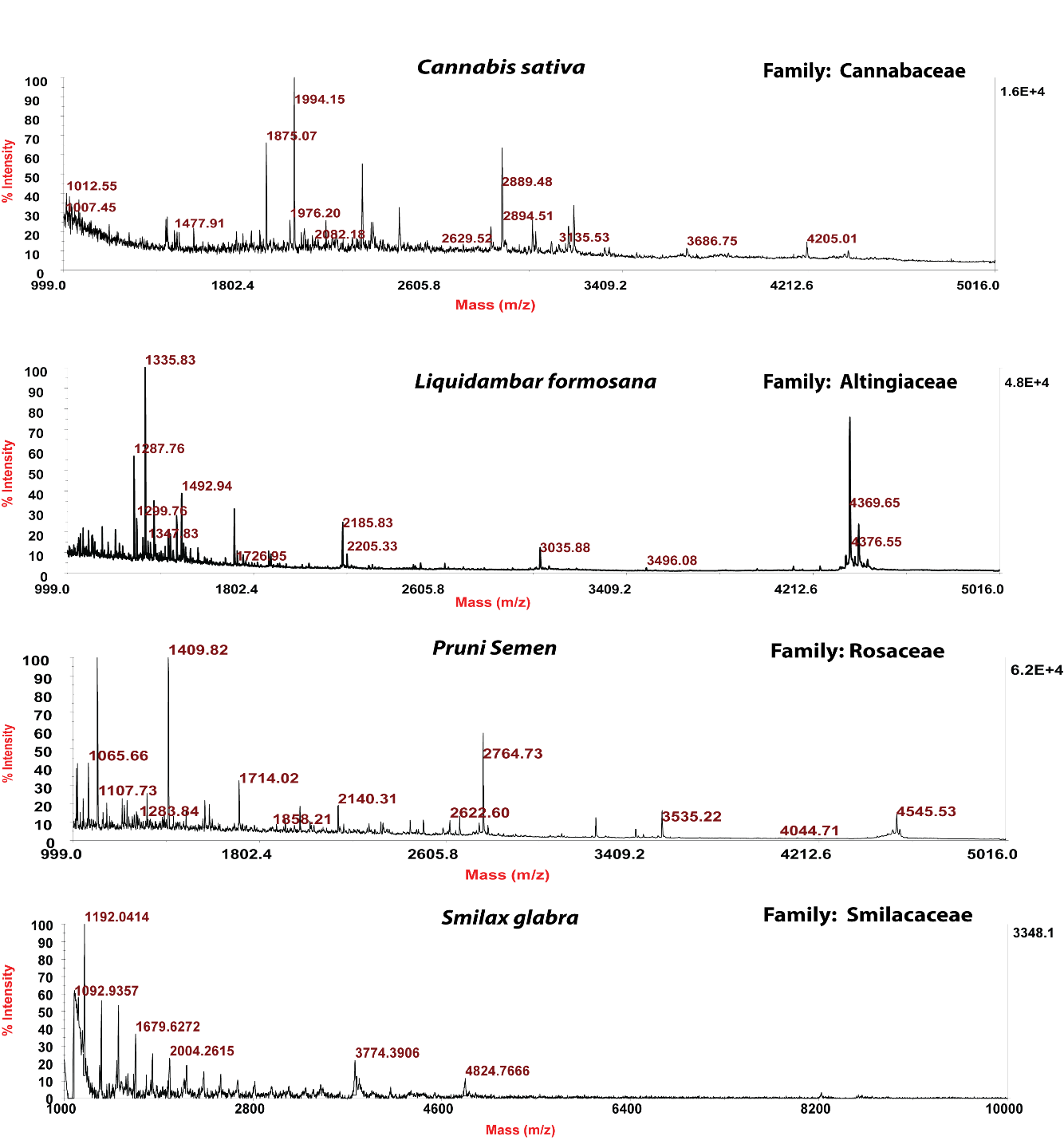
**

**Supplementary Figure S1.** MALDI-TOF mass spectra of 100 herbs and herbal products.


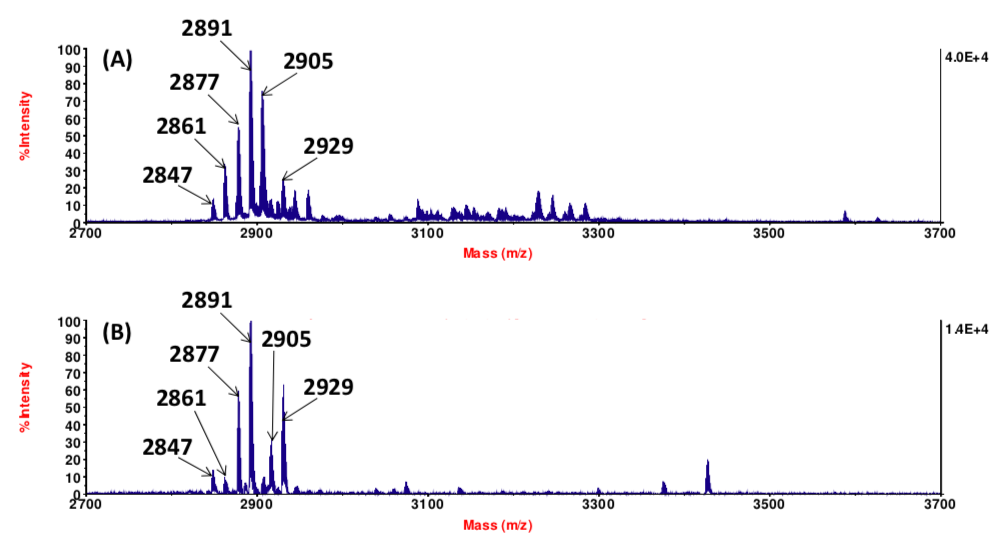


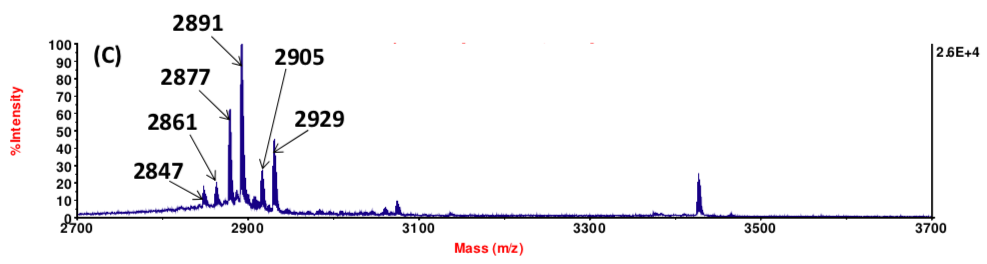


**Supplementary Figure S2.** MALDI-TOF mass spectra of *Viola yedoensis* in three different forms: (A) fresh herb, (B) dried herb and (C) granules.


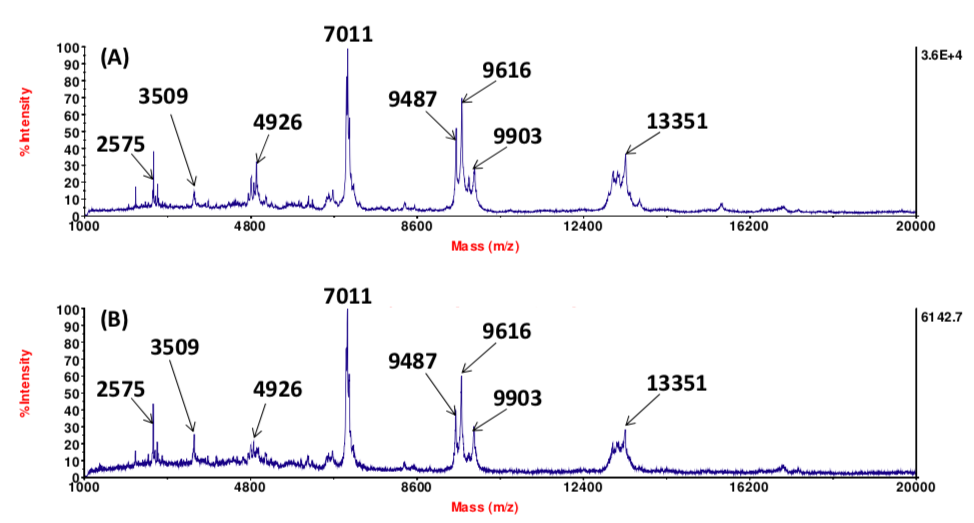


**Supplementary Figure S3.** MALDI-TOF mass spectra of the crude extract of *Triticum aestivum:* (A) before and (B) after heat treatment for 1 h.


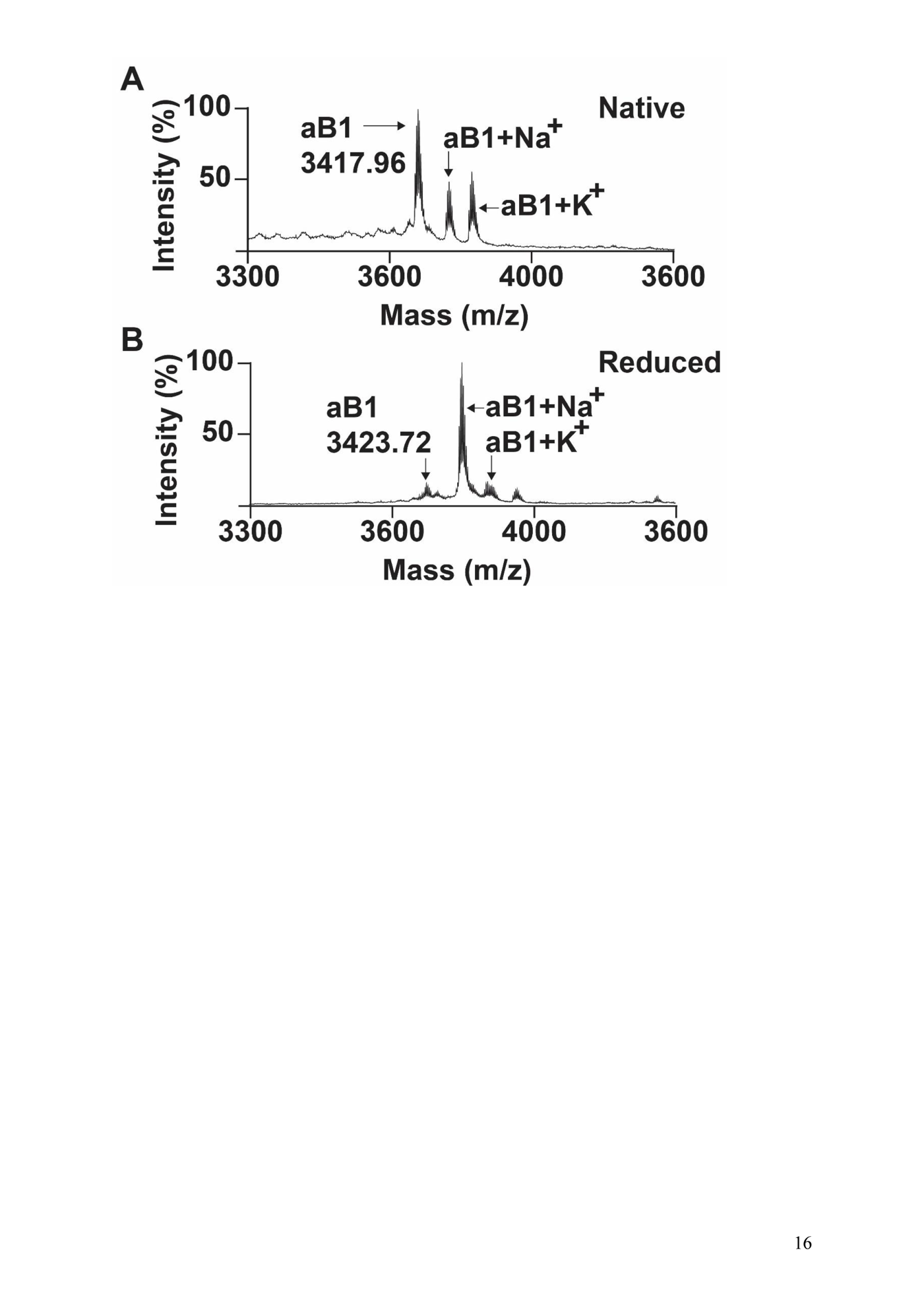


**Supplementary Figure S4.** MS profiles of achyrantide aB1 from *A. bidentate*. The mass spectra of aB1 (A) before and (B) after *S-*reduction, which showed a mass shift of 6 Da.


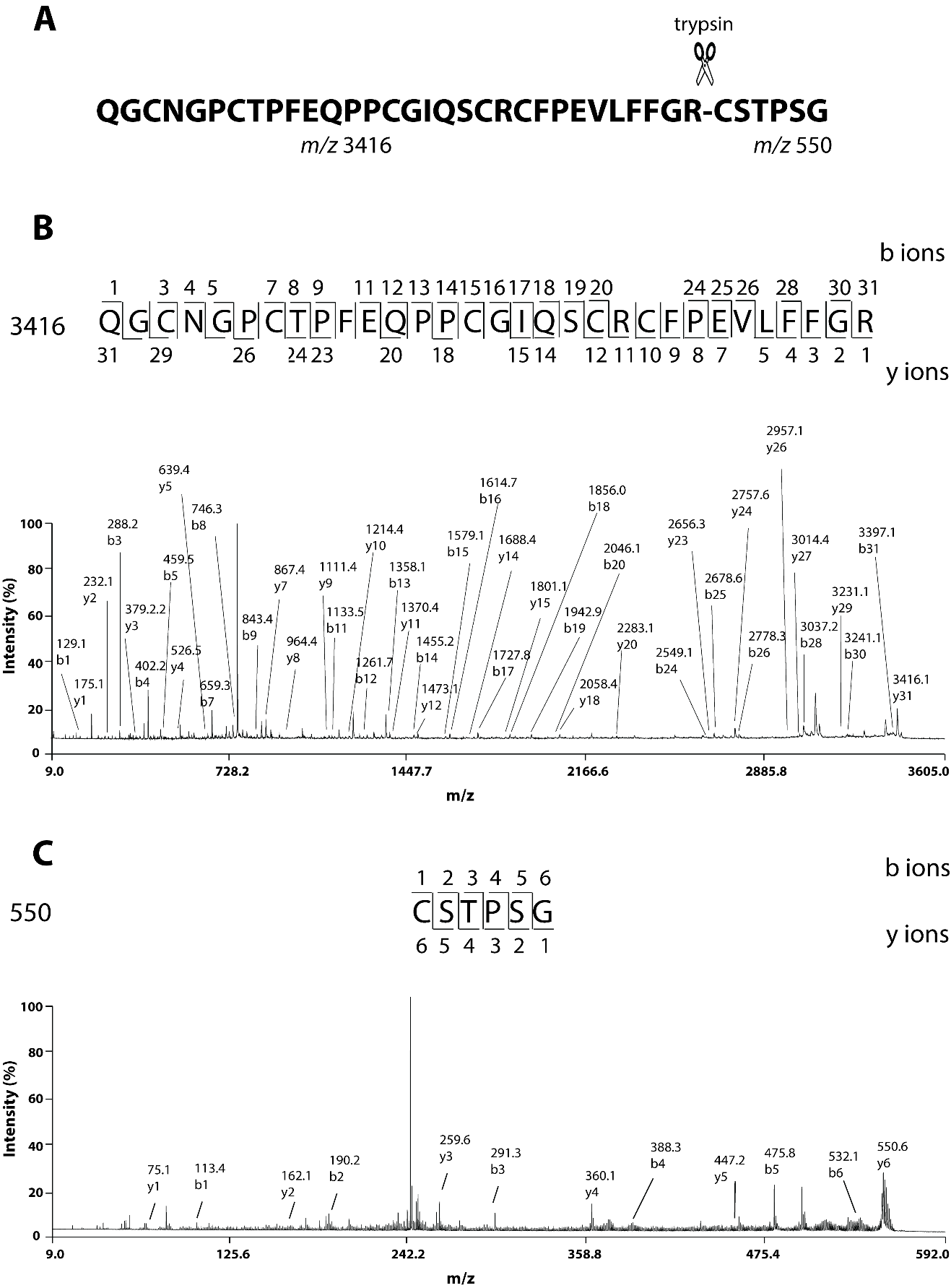


**Supplementary Figure S5.** MS/MS sequencing of hP1. (A) Trypsin digestion of heditide hP1 which yielded three fragments with m/z values 3416 and 550 Da; (B−C) MS/ MS spectra of digested fragments.

**
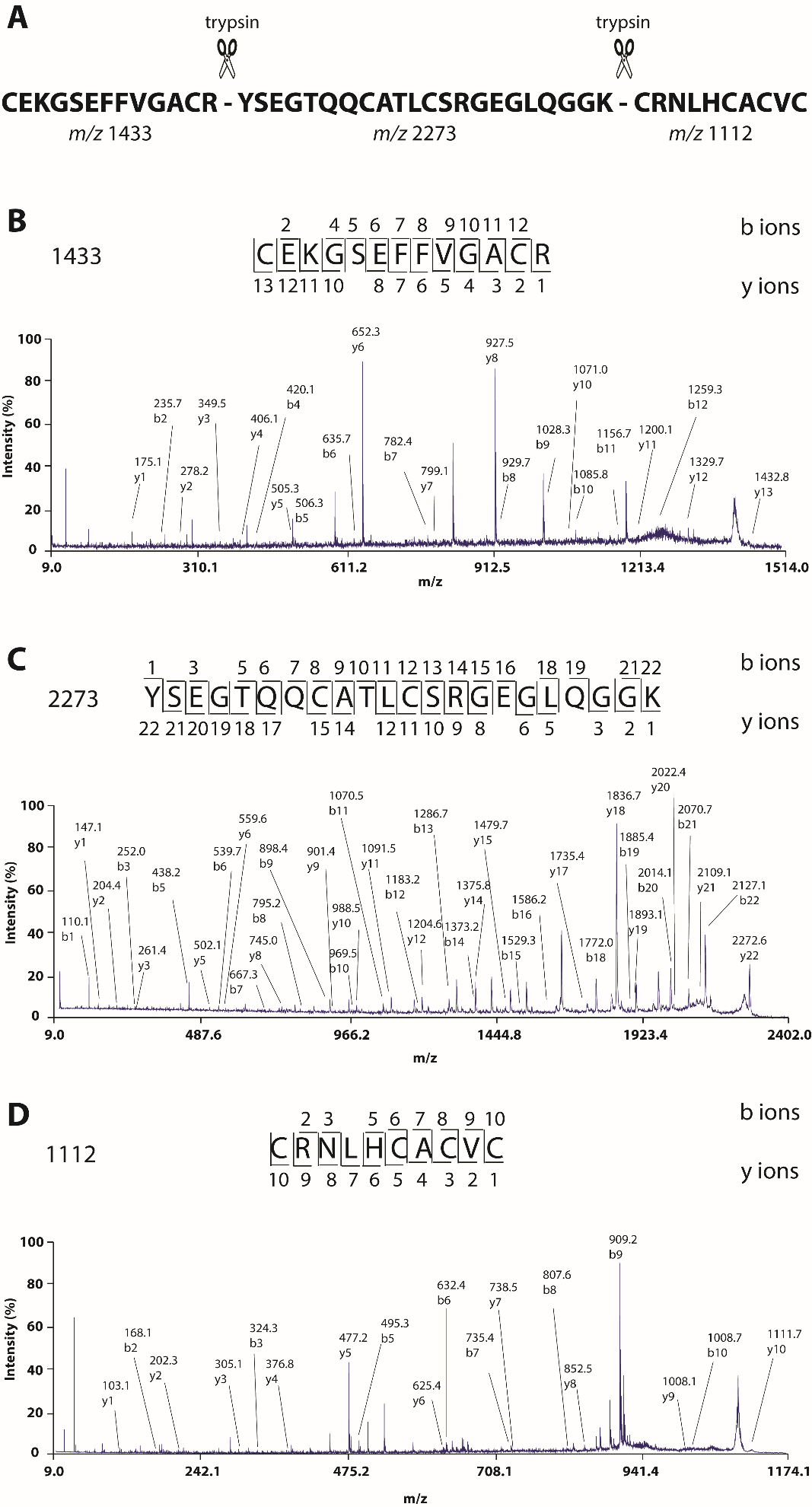
**

**Supplementary Figure S6.** MS/MS sequencing of hP2. (A) Trypsin digestion of heditide hP2 which yielded three fragments with m/z values 1433, 2272, and 1121 Da; (B−D) MS/ MS spectra of digested fragments.
